# Structural and chemical insights into the covalent-allosteric inhibition of the protein kinase Akt†
†Electronic supplementary information (ESI) available. See DOI: 10.1039/c8sc05212c


**DOI:** 10.1039/c8sc05212c

**Published:** 2019-02-13

**Authors:** Niklas Uhlenbrock, Steven Smith, Jörn Weisner, Ina Landel, Marius Lindemann, Thien Anh Le, Julia Hardick, Rajesh Gontla, Rebekka Scheinpflug, Paul Czodrowski, Petra Janning, Laura Depta, Lena Quambusch, Matthias P. Müller, Bernd Engels, Daniel Rauh

**Affiliations:** a Faculty of Chemistry and Chemical Biology , TU Dortmund University , Drug Discovery Hub Dortmund (DDHD) am Zentrum für integrierte Wirkstoffforschung (ZIW) , Otto-Hahn-Strasse 4a , 44227 Dortmund , Germany . Email: daniel.rauh@tu-dortmund.de ; http://www.ddhdortmund.de ; www.twitter.com/DDHDortmund; b Faculty for Chemistry and Pharmacy , Institut für Physikalische und Theoretische Chemie , Universität Würzburg , Emil-Fischer-Strasse 42 , 97074 Würzburg , Germany; c Max-Planck-Institut für Molekulare Physiologie , Abteilung Chemische Biologie , Otto-Hahn-Strasse 11 , 44227 Dortmund , Germany

## Abstract

The Ser/Thr kinase Akt (Protein Kinase B/PKB) is a master switch in cellular signal transduction pathways. Its downstream signaling influences cell proliferation, cell growth, and apoptosis, rendering Akt a prominent drug target. The unique activation mechanism of Akt involves a change of the relative orientation of its N-terminal pleckstrin homology (PH) and the kinase domain and makes this kinase suitable for highly specific allosteric modulation. Here we present a unique set of crystal structures of covalent-allosteric interdomain inhibitors in complex with full-length Akt and report the structure-based design, synthesis, biological and pharmacological evaluation of a focused library of these innovative inhibitors.

## Abstract 2

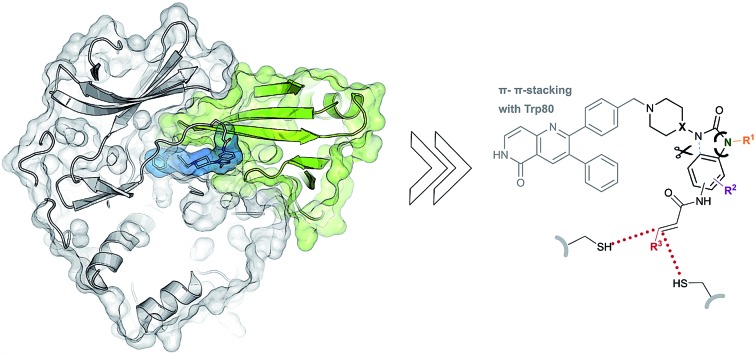
Structure-based driven synthesis and biological evaluation provide innovative novel covalent-allosteric Akt inhibitors.

## Introduction

The modulation of protein kinases with small molecule inhibitors accounts for a large share of the targeted therapy of a wide range of diseases including cancer, inflammatory diseases, diabetes, and neurodegenerative disorders.^1^,^2^ The crucial role of kinases with respect to protein phosphorylation in cellular signaling makes this class of proteins important and attractive targets in drug discovery.^3^ As a key player in the PI3K/Akt/mTOR-pathway, the Ser-/Thr-specific kinase Akt, also known as protein kinase B or PKB, reflects the significance of protein kinases in cellular processes.^4^ Alterations and dysregulation in the PI3K/Akt signaling pathway are related to different types of solid tumors such as lung, prostate, endometrial, cervical cancer, and melanoma.^5^ Furthermore, activating mutations of Akt as well as overexpression have been identified as disease drivers in certain metastatic breast cancers and are often related to resistance against chemo- and radiotherapy.^6^,^7^ These features suggest a promising potential of the targeted modulation of Akt with small molecule inhibitors in disease treatment and have motivated the development of selective Akt inhibitors in recent decades.^8^ A well-established approach in addressing protein kinases has been the development of orthosteric inhibitors that bind to the active site of the kinase domain in an ATP-competitive manner.^9^ A multitude of potent inhibitors such as ipatasertib, based on a cyclopentapyrimidine-scaffold, and the thiophenecarboxamide-derivative afuresertib have been identified and have entered phase I/II studies for mono- or combination therapy for a variety of indications.^10^–^14^ However, the ATP-binding pocket of Akt is highly conserved among all three isoforms of Akt and among several other kinases of the AGC kinase superfamily, making selectivity an issue for this type of inhibitors.^15^ In contrast to orthosteric inhibitors, allosteric kinase inhibitors that bind at remote sites of the protein are capable of inhibiting the kinase by stabilization of inactive conformations, and can lead to great benefits with respect to selectivity.^16^,^17^

**Fig. 1:**
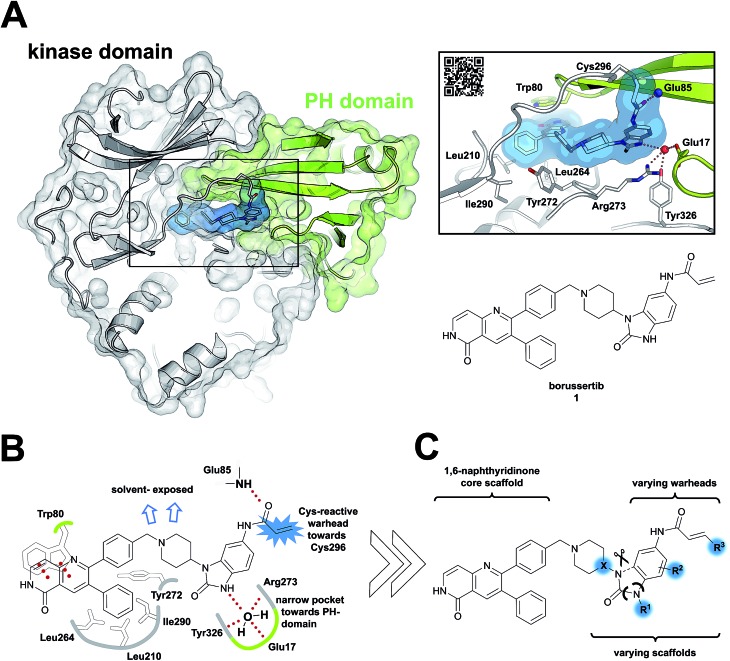
Covalent-allosteric Akt inhibitors irreversibly stabilize the inactive PH-in conformation. (A) Crystal structure of full-length Akt in complex with borussertib (**1**, highlighted in blue, PDB: ; 6HHF) shows covalent-allosteric mode of action while binding at the interdomain region between the kinase-domain (white) and the PH-domain (green). Detailed view of the binding mode of borussertib (right) reveals covalent bond formation to Cys296, H-bond interactions are illustrated with dotted lines. (B) Schematic representation of the key interactions of borussertib to the target protein illustrate crucial π–π-stacking with Trp80 and water-mediated H-bond-interactions. (C) Structural analysis inspired the design of novel derivatives to probe Akt functions.

Due to the pleckstrin homology (PH) domain-mediated regulation mechanism of Akt, targeting the interdomain region between the kinase and the PH domain enables the stabilization of the inactive “PH-in” conformation by allosteric inhibitors.^18^ Initially identified by serendipity, a small number of potent PH domain-dependent inhibitors have been developed to target this interdomain region and have resulted in the clinical lead candidates MK-2206 19–21 and miransertib.^22^–^24^ Besides their benefits in selectivity, it was shown recently that the conformation-dependent, but kinase-independent, functions of Akt are linked to cancer cell survival.^23^ Hence, stabilizers of distinct kinase conformations could contribute not only to a better understanding of this function of Akt beyond catalysis, but also pave the way for allosteric Akt inhibitors in a clinical setting.^25^,^26^ In view of this, we recently combined the characteristics of allosteric Akt modulators with the beneficial properties of irreversible inhibitors to result in covalent-allosteric inhibitors (CAIs).^27^ The first-in-class inhibitor borussertib (**1**) is based on the 1,6-naphthyridinone-scaffold and features a warhead to allow for the formation of a covalent bond to Cys296 *via* Michael addition, resulting in an increased potency and selectivity by maximization of the drug-target residence time.^28^ The evaluation of borussertib in meaningful cellular and xenograft models emphasized the inhibitory potency and *in vivo* efficacy of this novel class of inhibitors.^29^ The crystal structure in complex with full-length Akt provided crucial information about the binding characteristics (Fig. 1A–C). Based on these insights, we now report the structure-based design and synthesis of a focused library of covalent-allosteric inhibitors (Fig. 1C). The characterization of the inhibitory and kinetic properties as well as a series of complex crystal structures resulted in the first structure-activity relationship (SAR) of this innovative class of inhibitors. Furthermore, we demonstrate the potent inhibition of cell proliferation in a series of cellular models. By *in vitro* ADME profiling, we identified novel predestined candidates for further *in vivo* studies.

## Results

### Structure-based design

Structural information about the binding characteristics of covalent-allosteric inhibitors provides not only a detailed understanding of Akt modulation with this novel type of inhibitor, but also defines starting points for further optimization. The recently published co-crystal structure of full-length Akt with borussertib (PDB: 6HHF)^29^ showed that the compound was indeed bound between the PH- and the kinase domain to adopt the proposed allosteric binding mode (Fig. 1A and B). Most importantly, the crystal structure clearly revealed that the acrylamide warhead of borussertib formed a covalent bond with Cys296 of the kinase domain. The electrophilic group was further stabilized by an H-bond to the amide backbone of Glu85. The 1,6-naphthyridinone core scaffold resided at the interface between the activation loop of the kinase domain and the regulatory PH-domain, forming a favorable π–π-stacking interaction with the side chain of Trp80 of the PH-domain, as previously seen for different allosteric inhibitors in complex with Akt.^23^,^30^,^31^ The phenyl rings in the 3- and the 4-position of the naphthyridinone core interact with the kinase domain by sandwiching the side chain of Tyr272 and are located in hydrophobic regions flanked by Leu210, Leu264, and Ile290, thus adding to favorable protein–ligand interactions. Interestingly, the unsubstituted amide nitrogen of the imidazolone pointed into a hydrophilic pocket which was occupied by a conserved water molecule which mediates H-bond interactions to the amino acid side chains of Glu17 of the PH- and Tyr326 and Arg273 of the kinase domain. Structure–activity relationship (SAR) studies regarding this rather narrow pocket will be discussed below. Also, the 4- and 5-positions of the benzo[*d*]imidazolone core were solvent exposed. Thus, the crystal structure of borussertib inspired the structure-based design of novel probe molecules by directed derivatization. Toward this end, we developed a robust synthetic route that enabled the synthetic access to a series of highly diverse 1,6-naphthyridinone-based compounds.

### Structure-based design-driven synthesis

**Scheme 1:**
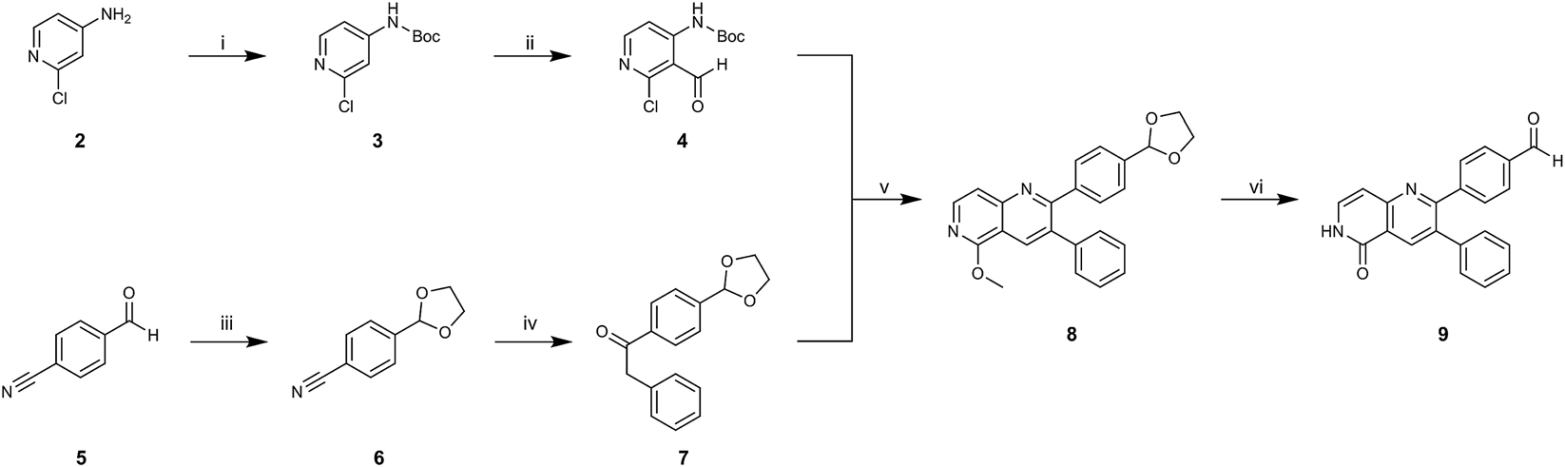
Synthesis of 1,6-naphthyridinone core scaffold by a divergent synthetic route. (i) Boc_2_O, DMAP, TEA, DCM, rt, 2 h. (ii) ^*t*^BuLi, DMF, THF, –76 °C – rt, ovn. (iii) Ethylene glycol, *p*-TsOH, toluene, reflux, ovn. (iv) Benzylmagnesium chloride, THF, 0 °C – rt. (v) Sodium methoxide, MeOH, 65 °C, 4 h. (vi) HCl_aq_, reflux, 3 h.

To acquire insights into the structure–activity relationship of 1,6-naphthyridinone-based covalent-allosteric inhibitors, we developed a robust synthetic route to generate a focused library of Akt inhibitors. Novel inhibitors were designed based on the insights of the structural analysis of the crystal structure of borussertib in complex with Akt (Fig. 1C). More precisely, the importance of the water-mediated hydrogen bond was investigated by modifications at the N1-position of the benzo[*d*]imidazolone moiety. Furthermore, we investigated how altering the flexibility and linker length between the 1,6-naphthyridinone core and the acrylamide warhead impact the preorganization of the inhibitors towards the targeted cysteine residues. We addressed this issue by replacing the benzo[*d*]imidazolone by scaffolds of different length, flexibility and substitution pattern (ESI Fig. S18†). In line with our previous study, the retrosynthetic analysis revealed the convergent division in 1,6-naphthyridinone building block **9** and the benzo[*d*]imidazolone **13** (Scheme 1).^27^,^32^ Synthesis of **9** was dissected into two synthetic pathways starting from pyridine-derivative **2** and *p*-cyanobenzaldehyde (**5**). Compound **2** was Boc-protected and formylated in order to obtain intermediate **4**.^33^ In the second synthetic path, the aldehyde of **5** was initially protected with ethylene glycol and then converted by means of a Grignard reaction to the acetophenone-based intermediate **7**. Friedländer quinoline synthesis, coupled with an *in situ* Boc-deprotection, allowed the subsequent combination of **4** and **7** to the 1,6-naphthyridinone scaffold **8**, which could then be deprotected to obtain the desired building block **9**.^34^

**Scheme 2:**
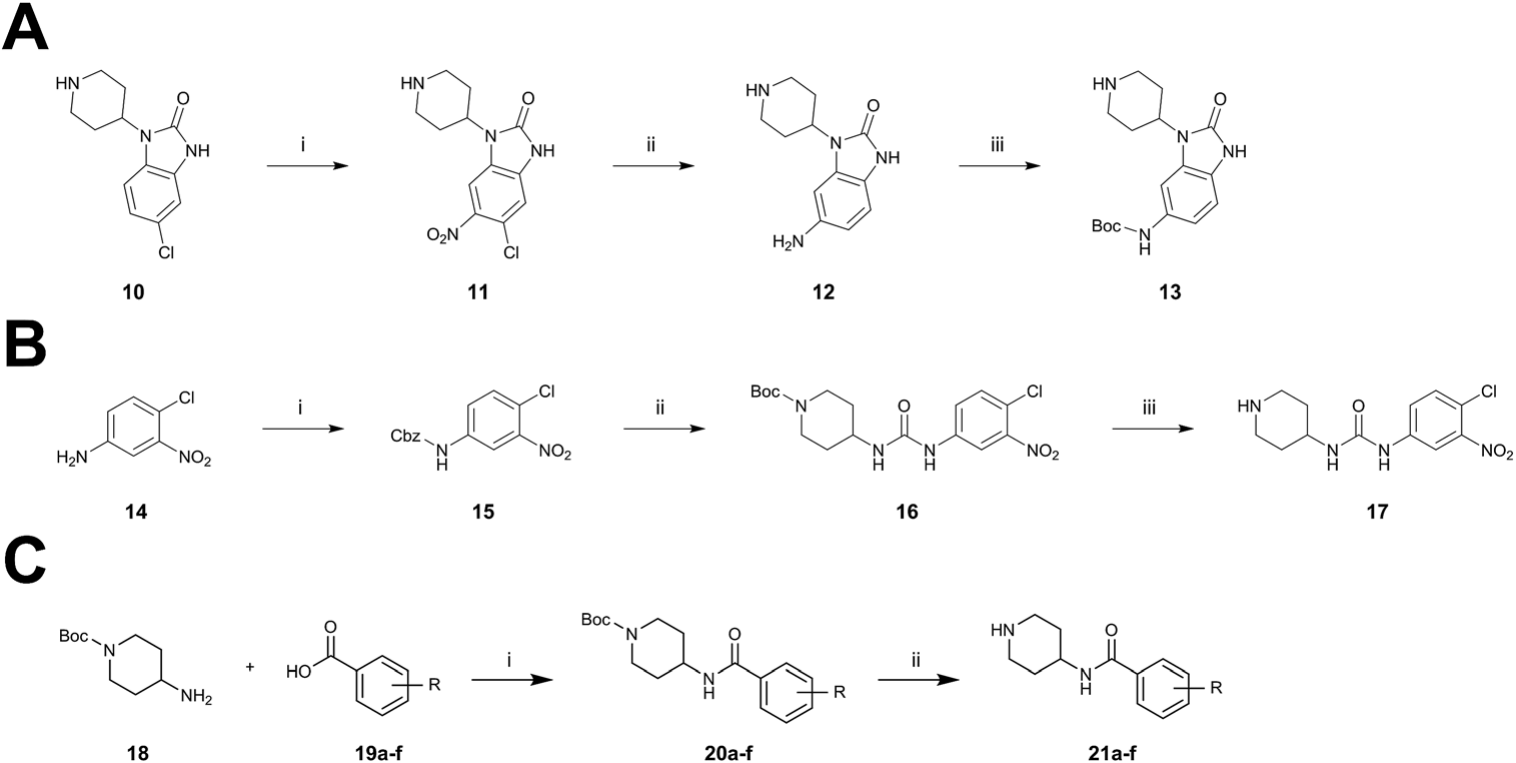
Synthesis of diverse building blocks for the derivatization of covalent-allosteric Akt inhibitors. (A) Synthetic scheme for synthesis of benzo[*d*]imidazolone building block. (i) HNO_3_, *o*-xylene, 60 °C, 2 h. (ii) Pd/C, NH_4_HCOO, MeOH, reflux, ovn. (iii) Boc_2_O, 10% AcOH/H_2_O, 1,4-dioxane, rt, ovn. (B) Synthetic scheme for synthesis of urea-based building block. (i) Cbz-Cl, NaHCO_3_, THF, rt, 5 h. (ii) **18**, K_2_CO_3_, DMF, 130 °C, μW. (iii) 4 N HCl/1,4-dioxane, rt, 2 h. (C) Synthetic scheme for synthesis of phenylamide-based building blocks. (i) HATU, 2,6-lutidine, DMF, rt, ovn. (ii) 4 N HCl/1,4-dioxane, rt, 2 h.

**Scheme 3:**
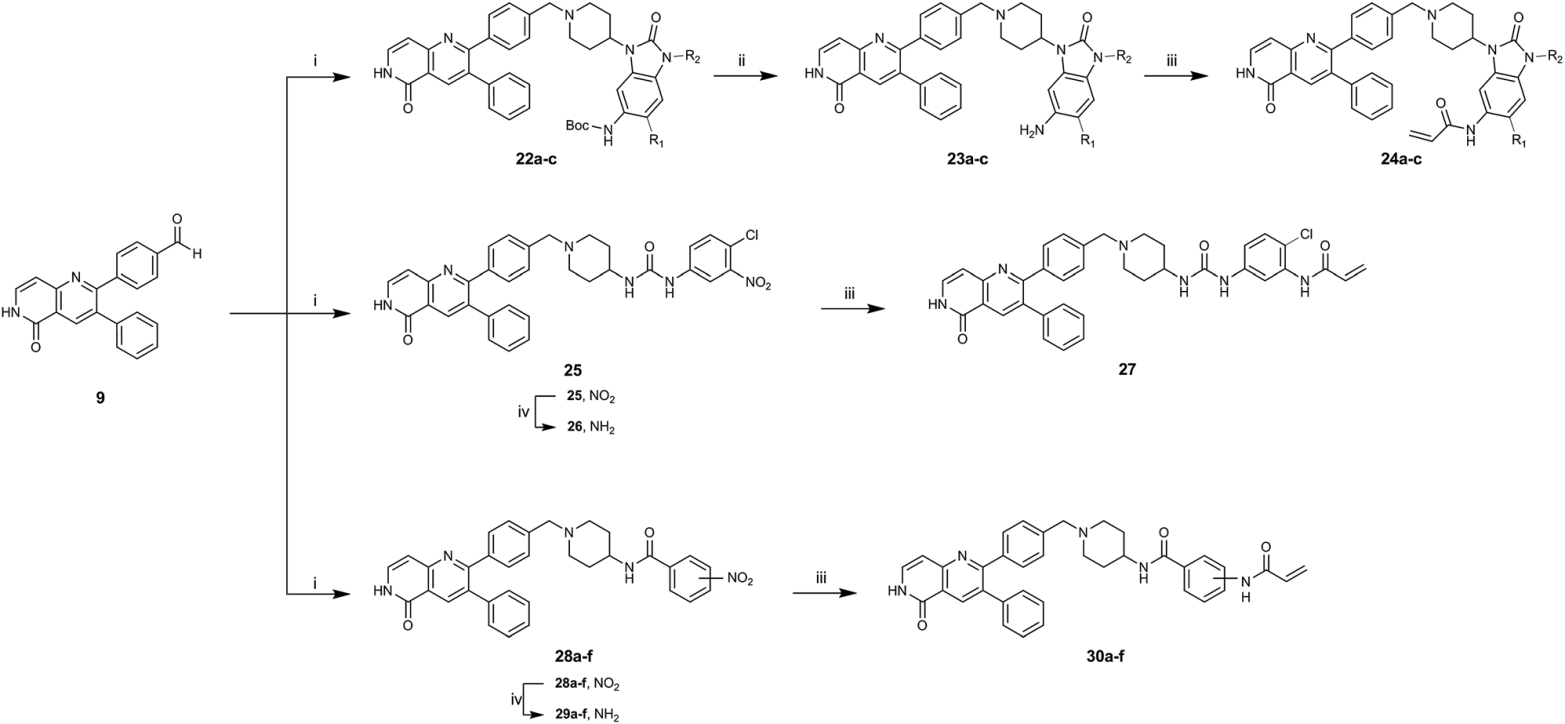
Synthetic scheme for the synthesis of covalent-allosteric inhibitor derivatives by assembling building blocks with Leuckart–Wallach-reaction. (i) Amine, formic acid, MeCN, 80 °C, ovn. (ii) 4 N HCl/1,4-dioxane, rt, 2 h. (iii) Acryloyl chloride, DIPEA, THF, rt, 3 h. (iv) Fe, NH_4_Cl, MeOH/H_2_O, 80 °C, 2 h.

The synthesis of the benzo[*d*]imidazolone scaffold started from a selective nitration of **10** to afford intermediate **11** (Scheme 2A).^35^ Pd-catalyzed hydration of the nitro-group into the amine and reductive dehalogenation succeeded in one step.^36^ The desired product **13** was obtained after selective Boc-protection of the anilinic amine.^37^ Great efforts were made to optimize the reductive amination, combining the two major building blocks **9** and **13**. Ultimately, we found a Leuckart–Wallach amination using formic acid as the reducing agent gave the best results.^38^ Deprotection and attachment of the acrylamide yielded the desired product (Scheme 3, **24a–c**). Besides dehalogenation and simple alkylation reactions at the N1-position (synthesis shown in ESI†), the benzo[*d*]imidazolone core gave a very restricted scope regarding chemical space of possible derivatives. Consequently, we decided to change the scaffold in order to enable access to a greater variety of potential inhibitors. One option was the ring-opening of the imidazolone moiety leading to phenylurea-based derivatives (Scheme 2B). After initial Cbz-protection of 3-nitro-4-chloroaniline (**14**), urea formation (**16**) occurred derived from literature procedure with 4-amino-Boc-piperidine under microwave conditions (**18**).^39^ Acidic hydrolysis of the Boc-protecting group led to another fragment that was capable of attaching to the 1,6-naphthyridinone-scaffold by Leuckart–Wallach reaction.

Hence, the desired compound was obtained by reduction of **25** and attachment of the Michael acceptor. Furthermore, phenylamide-derivatives were considered to be an attractive alternative scaffold for optimization. Based on a variety of benzoic acids (**19a–g**), an amide coupling of these compounds with 4-amino-Boc-piperidine (**18**) and subsequent deprotection yielded novel building block that could be combined with the core scaffold by Leuckart–Wallach amination (Schemes 2C and 3). By reduction and acrylamide attachment, the phenylamide-based probe molecules (**30a–g**) were obtained. In addition, the variation of the acrylamide warhead provided another opportunity for modifications. Instead of the undecorated acrylamide, the introduction of Michael acceptors carrying a dimethylamino solubilizing group were expected to mediate an improved solubility and additionally serve as an intramolecular base to catalyze the Michael addition.^40^ In order to analyze the influence of the piperidine-based linker region, piperazine-based molecule **31** and benzylamine-based compound **32** were synthesized by related synthetic routes (synthesis shown in ESI†). In total, 13 novel inhibitors with different substitution patterns were synthesized. The biochemical and cellular evaluations of these molecules should lead to insights into the SARs of covalent-allosteric Akt inhibitors.

### Biochemical characterization and SAR analysis

For activity-based characterization of the small focused 1,6-naphthyridinon-based library, we measured the inhibitory potency (IC_50_) of all synthesized compounds and important intermediates towards full-length Akt (Table 1). In addition, a representative selection of these molecules was further investigated with respect to their kinetic profiles. In large, the covalent allosteric inhibitors showed superior potency with half-maximal inhibitory activity in the nanomolar to subnanomolar range when compared to the clinical candidate MK-2206. Furthermore, an evaluation of the ratio of *k*_inact_/*K*_i_, a parameter which combines both affinity and covalent bond formation, indicated that these inhibitors had an outstanding kinetic profile. Small substitutions at the 5-position of the benzo[*d*]imidazolone-core as shown for the 5-chlorinated derivative **24a** were tolerated, but showed 2-fold lower IC_50_ values, respectively. Interestingly, the N-methylation of the benzo[*d*]imidazolone in **24b** causes a 3-fold loss in inhibitory activity, suggesting that also methylation at this position is well tolerated. However, such a mild drop in potency let us assume that this water molecule is not energetically unfavorable. In addition, the elongated electrophile of **24c** showed a 20-fold decrease in potency indicating no positive influence of the dimethylamino-group on inhibitor binding. In order to extend access to more diverse compounds occupying a broader chemical space, we varied the benzo[*d*]imidazolone scaffold by opening the imidazolone-ring to obtain phenylurea-derivatives.

**Table 1:** Biochemical evaluation of covalent-allosteric Akt inhibitors

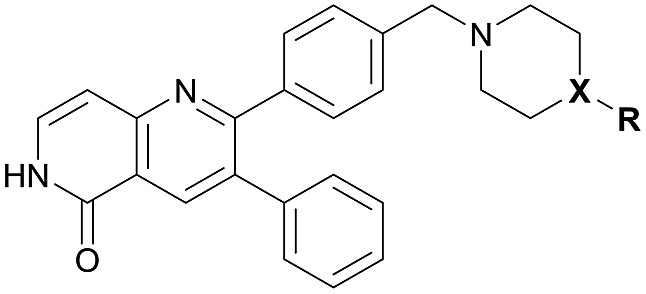						
Cpd	R	X	Akt^wt^			
			IC_50_ [nM]	*K*_i_ [nM]	*k*_inact_ [min^–1^]	*k*_inact_/*K*_i_ [μM^–1^ s^–1^]
**1**	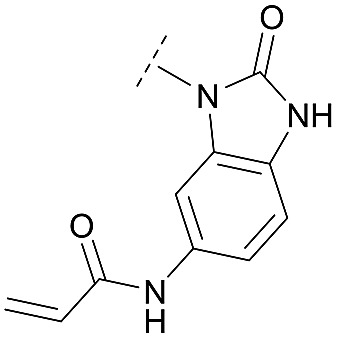	C	0.8 ± 0.3	2.2 ± 0.3	0.111 ± 0.020	0.853 ± 0.038
**24a**	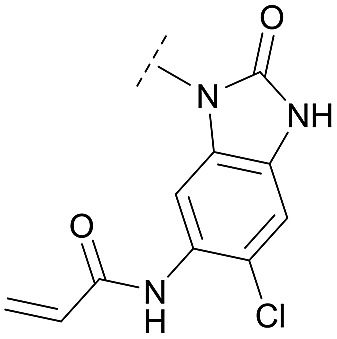	C	1.2 ± 0.3	4.1 ± 0.7	0.110 ± 0.023	0.447 ± 0.074
**24b**	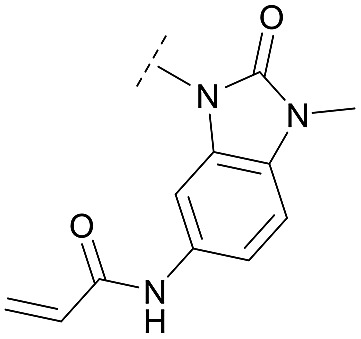	C	3.0 ± 0.3	10.7 ± 0.5	0.121 ± 0.016	0.190 ± 0.025
**24c**	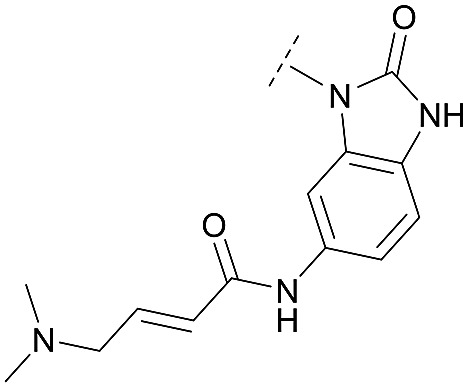	C	18.1 ± 4.9	33.0 ± 2.4	0.050 ± 0.009	0.025 ± 0.005
**27**	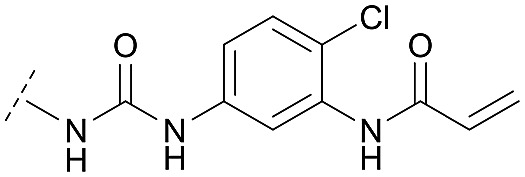	C	9.1 ± 1.5	17.5 ± 3.6	0.081 ± 0.019	0.080 ± 0.008
**30a**	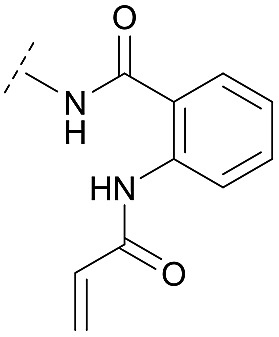	C	10.8 ± 2.5	27.2 ± 4.2	0.138 ± 0.031	0.085 ± 0.018
**30b**	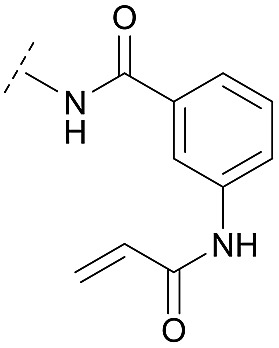	C	3.6 ± 0.8	6.8 ± 0.3	0.083 ± 0.016	0.202 ± 0.035
**30c**	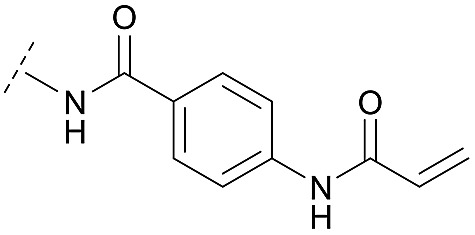	C	285 ± 65	362 ± 74	0.042 ± 0.007	0.002 ± 0.001
**30d**	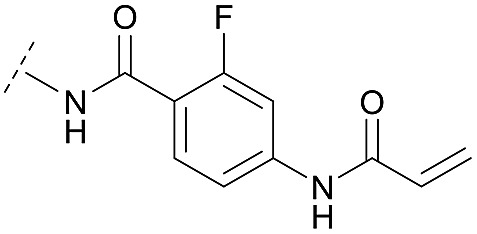	C	52.8 ± 16.3	98.6 ± 10.0	0.085 ± 0.008	0.014 ± 0.002
**30e**	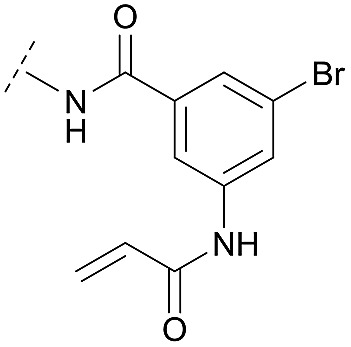	C	3.7 ± 0.7	7.6 ± 1.1	0.078 ± 0.022	0.168 ± 0.025
**30f**	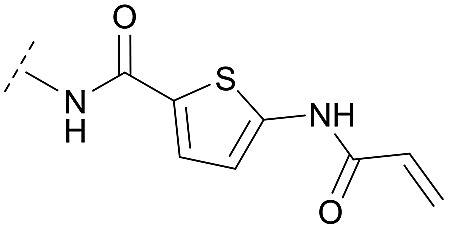	C	14.0 ± 6.1	39.3 ± 6.6	0.088 ± 0.010	0.038 ± 0.007
**31**	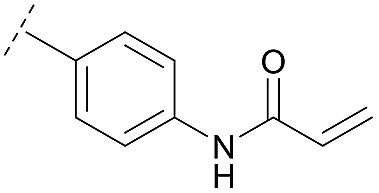	N	126 ± 31	67.1 ± 5.5	0.054 ± 0.006	0.013 ± 0.001
**32**	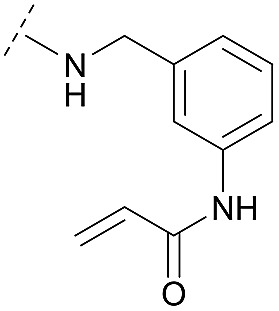	C	192 ± 45	327 ± 27	0.071 ± 0.004	0.004 ± 0.001
	Capivasertib		0.9 ± 0.1	—	—	—
	Ipatasertib		3.5 ± 0.6	—	—	—
	MK-2206		10.0 ± 2.1	—	—	—

**Fig. 2:**
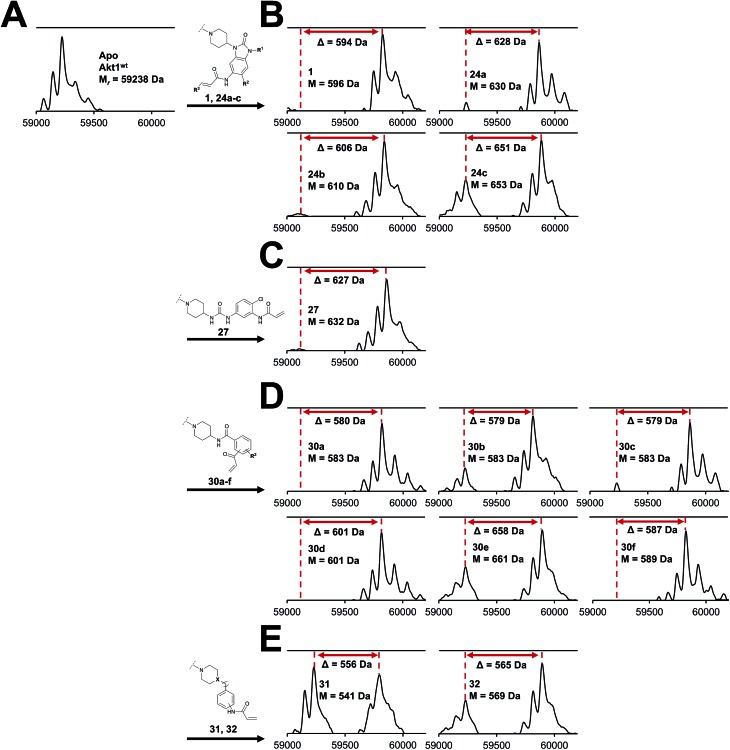
Deconvoluted mass spectra of Akt1^wt^ after incubation with DMSO (Apo, (A)) and a small focused library of covalent-allosteric Akt inhibitors showed a mass shift (displayed as *Δ* values) for all tested inhibitors with mass differences according to a labeling of the protein. (B) Mass spectra of benzo[*d*]imidazolone-based inhibitors. (C) Mass spectrum of urea-based inhibitor. (D) Mass spectra of phenylamide-based inhibitors. (E) Mass spectra of inhibitors **31** and **32**. Mass spectra were recorded using denaturing conditions.

Although a 10-fold loss in activity was observed compared to **1**, an evaluation of compound **27** revealed that phenylurea-based probes represent covalent-allosteric inhibitors with a novel scaffold that inhibited Akt in the nanomolar range. On the other hand, phenylamide-based inhibitors illustrated an alternative scaffold that, in contrast to benzo[*d*]imidazolones, allowed access to other diverse derivatives (**30a–f**, Table 1). In general, our compounds exhibited half-maximal inhibition of Akt at nanomolar concentrations, indicating that these phenylamide derivatives were suitable for covalent allosteric inhibition. The orientation of the Michael acceptor was varied by decoration of the acrylamide function at the *ortho*-, *meta*- and *para*-positions. However, the *ortho*-substituted compound **30a** and the *meta*-substituted compound **30b** were favored over **30c** carrying the acrylamide in the *para*-position, which is consistent with the idea that the orientation of the warhead towards the targeted cysteines plays an important role in irreversible inhibitor binding and occurs preferentially in the *ortho*- and *meta*-positions. On closer consideration of the amide-bridged linker region of phenylamide-based inhibitors, compound **32**, which omitted the carbonyl function, showed a lack of activity. We hypothesize that this is a conformation-driven effect. This suggests that leaving out the amide function led to a loss of structural integrity of the inhibitor. Furthermore, as observed for molecule **31**, these phenyl amides were characterized by a superior potency in comparison to phenylpiperazine-based probes that were distinguished by a shortened molecule length. It is worth noting that the replacement of the phenyl amide by the bioisosteric thiophene amide **30f** was well tolerated.^41^ The kinetic evaluation of these inhibitors already evinced the covalent binding character of these type of molecules. In order to provide an orthogonal proof for covalent bond formation, we co-incubated the covalent-allosteric inhibitors with the protein Akt1 and analyzed the samples by mass spectrometry (Fig. 2). Although the resolution of the deconvoluted mass spectra was low due to differently phosphorylated forms of Akt1, all spectra showed a mass shift in comparison to the apo protein with mass differences according to a mono-labeling of Akt1 with the corresponding inhibitor. While molecules with a high inhibitory activity as shown for **1**, **24b** (Fig. 2B) and **27** (Fig. 2C) showed complete labeling of the protein, inhibitors with a lower potency such as **31** showed incomplete labeling within 1 h (Fig. 2E). In concert with the biochemical analysis, these experiments gave further qualitative evidence for selective covalent modification of the target protein.

To further investigate inhibitor selectivity over other protein kinases, we evaluated the benzo[*d*]imidazolone **24b**, the phenylurea **27** and the phenylamide **30b** in a profiling study encompassing 100 selected kinases with similar characteristics such as AGC kinase family members, PH-domain containing kinases and kinases containing analogous cysteines on the activation loop (SelectScreen Kinase Profiling Service from ThermoFisher Scientific, Tables S2–S4 and Fig. S19 in the ESI†). At a concentration of 1 μM, all compounds show a potent inhibition of all three Akt isoforms with a notable preference of the Akt1 and Akt2 isoforms. For **24b** and **30b**, we observed a selectivity profile similar to borussertib with poor inhibition of the MELK kinase (48% by **24b** and 52% by **30b**, respectively). For **30b**, we detected additional moderate inhibitory activity against the AMP-activated protein kinases A1/B1/G1 (46%) and A2/B1/G1 (40%) and non-receptor tyrosine kinase BMX (44%). Interestingly, we noticed subtle differences in the selectivity profile of phenylurea-based compound **27**, since we observed a more potent inhibition of MELK kinase (83%), AMPK A1/B1/G1 (77%) and A2/B1/G1 (77%) and besides a moderate inhibition PASK (61%) and ROCK1 (54%). Of particular note is that all these kinases contain at least one analogous cysteine on the activation loop. All other kinases showed less than 40% inhibition emphasizing the fact that covalent-allosteric inhibitors are characterized by a promising selectivity profile. The structure-based design and biochemical evaluation of the small-focused library of covalent allosteric inhibitors revealed phenylurea- and phenylamide-derived molecules as Akt probes with novel scaffolds that opened chemical space for optimization. The binding mode of these compound classes was further investigated by X-ray crystallography.

### Structural analysis

**Fig. 3:**
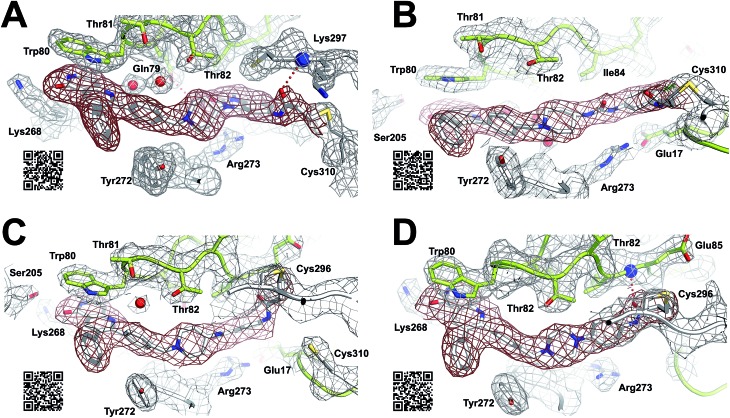
Co-crystal structures of full-length Akt in complex with different covalent-allosteric inhibitors, 2F_o_–F_c_ maps contoured at 0.8*σ*. (A) Co-crystal structure of Akt with **24b** (PDB: ; 6HHJ) and (B) with **27** (PDB: ; 6HHG) revealed novel binding mode while labeling Cys310. (C) Co-crystal structure of Akt with **30b** (PDB: ; 6HHI) and (D) with **31** (PDB: ; 6HHH) exhibited similar binding mode to borussertib while labeling Cys296.

For a deeper understanding of the binding mode of the novel Akt inhibitors, we co-crystallized full-length Akt with benzo[*d*]imidazolone-derivative **24b**, phenylurea-derivative **27**, the phenylamide-derivative **30b** and the phenylpiperazine-based ligand **31** (Fig. 3A–D). A more detailed close-up of the protein–ligand interactions is given in ESI Fig. S20.† All four complex structures confirmed the expected covalent-allosteric mode of action while binding to the interdomain region between kinase and PH-domain. Furthermore, the naphthyridinone-core of these inhibitors adopted similar conformations to the previously described binding mode of borussertib by π–π-stacking interaction with Trp80. Surprisingly, the crystal structures of **24b** and **27** showed that the electrophile warhead did not form a covalent bond to Cys296 as it was initially shown in the crystal structure of borussertib, but covalently labeled the nearby Cys310. This alternative binding mode also caused a different interaction profile of these ligands within the binding pocket (ESI Fig. S20A and B†). For **24b**, the additional N-methylation impeded the polar contact of this amine to the conserved water molecule in the narrow polar pocket as shown in the crystal structure of borussertib. Consequently, the piperidine ring adopted an altered conformation inducing a shift of the benzo[*d*]imidazolone-core and thus enabled the covalent bond formation to Cys310 and additional H-bond interactions to the amide backbone of Lys297 and water-mediated to the side chain of Gln79. In case of **27**, a small shift of ligand conformation caused labeling of Cys310 as well as H-bond formation of the acrylamide amine to the protein backbone of Glu17. The shift seemed to be induced by the extended molecule structure of the urea-based compound in comparison to the benzo[*d*]imidazolone- and phenylamide-based inhibitors. This assumption was further confirmed by the co-crystal structure of Akt with **30b** and **31**, given that both molecules with shortened molecule length revealed a covalent modification of Cys296 and a similar binding mode compared to borussertib. The interaction of the latter inhibitors was additionally stabilized by formation of an H-bond to the amide nitrogen of Glu85 (ESI Fig. S19C and D†). Hence, the crystal structure analysis of these covalent-allosteric inhibitors showed different prioritization in labelling the adjacent residues Cys296 and Cys310. Interestingly, previously described MS/MS analysis of borussertib showed a covalent modification of both cysteines.^27^ We did additional MS/MS studies for inhibitor **27** and obtained similar results (ESI Fig. S21†). Therefore, we further investigated the labeling of the residues of Cys296 and Cys310 by different inhibitors by molecular dynamic (MD) simulations. The p*K*_a_ values of cysteine residues strongly correlate with their reactivity because deprotonated cysteine residues are considerably more reactive.^42^,^43^ Based on the similarities of their enzyme environment Cys296 and Cys310 are expected to possess equal p*K*_a_ values and reactivities.^44^ In such cases the reaction probability of the sulfur of a cysteine moiety with the electrophilic center of the inhibitor is mainly determined by their distance in the non-covalent enzyme inhibitor complex, *i.e.* before the reaction.^45^–^48^ Against this background, we used theoretically prepared non-covalent enzyme–inhibitor complexes and conducted MD simulation in which we evaluated the distances of the acrylamide electrophile to the sidechain sulfur of Cys296 and Cys310 over the simulation time (ESI Fig. S22–S24†). For borussertib (ESI Fig. S19†), our MD simulations predict distances of 3–4 Å between the electrophilic center of the inhibitor and the nucleophilic sulfur of the Cys296 residue which is sufficiently short that a reaction can take place. Additionally, the simulations indicated only small variations in the distances between both centers which is also favorable for a reaction.^45^,^46^ The computed distances between the electrophilic center of the inhibitor and the nucleophilic sulfur of Cys310 are larger for most of the simulation time, although distances which enable a reaction are rarely adopted. Summarizing, our simulations predict reactions to Cys296 and Cys310, but the reaction to Cys296 is clearly preferred. This nicely explains the apparent contradiction between crystallographic and MS/MS experiments. For compound **24b** (ESI Fig. S20†) the computed distances between the electrophilic center and Cys310 become smaller and the corresponding fluctuations decrease in comparison to the situation of borussertib. In contrast, the distances and the fluctuation of the distances to Cys296 increase. This implies that for **24b** the reaction with Cys310 becomes more probable hence is in line with the experimental crystal data which indicate a covalent bonding to Cys310. In case of **27**, an even higher preference to Cys310 was ascertained which was reflected by the fact that during simulation time the distance between the electrophile of **27** and Cys296 never adopted distances that enable covalent bond formation. Although MS/MS analysis showed a Cys296-labeled species which was not detectable during the MD simulations, these results are in good correlation with the crystallography studies and indicate that the covalent bond formation of **27** to Cys310 is clearly favored.

### Cellular evaluation

The biochemical and structural analysis of the small synthesized library revealed probe molecules acting as covalent-allosteric inhibitors of Akt. In order to proof their activity in cellular systems, we tested the inhibitors and we determined the half-maximal effective concentration (EC_50_)-values (Table 2). The tested cell lines AN3CA (endometrium), T47D (breast), ZR-75-1 (breast), MCF-7 (breast), BT-474 (breast) and KU-19-19 (bladder) are described to be sensitive against Akt inhibition.^23^,^49^ All cell lines exhibit genetic alterations in the PI3K/Akt and Ras/MAPK pathways, including in-frame deletions in PIK3R1, loss-of-function or downregulating genetic lesions in PTEN or TP53 or activating point mutations in PI3KCA, Akt1 and NRas. Evidently, the tested cell lines AN3CA, T47D, ZR-75-1, MCF-7, BT-474 and KU-19-19 are dependent on Akt signaling considering that both reference compounds and the majority of the CAI-library were able to inhibit cell proliferation. As figured out in recent studies, the breast cancer cell line ZR-75-1 showed the highest sensitivity against Akt inhibition and, more particularly, the first-in-class covalent-allosteric inhibitor borussertib exhibited excellent cellular activity in the nanomolar range with superior profile against clinical candidate Akt inhibitors as well as the cytostatic drug doxorubicin. Interestingly, the chlorinated derivative of borussertib **24a** and the methylated derivative **24b** evoked a 2-fold higher inhibitory activity with low nanomolar EC_50_-values for ZR-75-1 inhibition and similar tendencies for the endometrium cancer cell line AN3CA and the breast cancer cell lines T47D, MCF-7 and BT-474. The other benzo[*d*]imidazolone derivative **24c** showed lower cellular activity with a 200- and 100-fold reduction of EC_50_, respectively. For the novel urea-based inhibitor **27**, a good cellular inhibition could also be determined, in particular with 39 nM inhibition of ZR-75-1, however, a 20-fold decrease in activity was observed in comparison to **24a**. At a glance, the series of phenylamide-based inhibitors showed an outstanding inhibitory profile on all different cell lines, whereas **30b** was identified to be the most active covalent-allosteric inhibitor in this series. Furthermore, a good correlation between effect on cell viability in different cancer cells that were previously shown to be sensitive towards Akt inhibition and the enzymatic inhibition of Akt was detected. For instance, the *meta*-derivative **30b** exhibited superior inhibitory activity compared to its *ortho*-derivative **30a** and its *para*-derivative **30c**.

**Table 2:** Cellular evaluation of covalent-allosteric Akt inhibitors

	Cell viability assay EC_50_ [nM]					
	AN3CA	T47D	ZR-75-1	MCF-7	BT-474	KU-19-19
PIK3R1^*a*^	R557_K561>Q					
PIK3CA^*a*^		H1047R		E545K	K111N	R1023Q
PTEN^*a*^	R130 fs		L108R			
AKT1^*a*^						E17K/E49K
NRAS^*a*^						Q61R
TP53^*a*^		L194F			E285K	

Capivasertib	869 ± 278	475 ± 92	191 ± 68	555	1605 ± 450	20 866 ± 7603
Ipatasertib	925 ± 457	443 ± 6	219 ± 83	5036 ± 1830	2371 ± 745	24 037 ± 9491
MK-2206	972 ± 322	583 ± 245	63 ± 21	571 ± 111	1682 ± 316	7054 ± 421
Doxorubicin	30 ± 9	127 ± 40	100 ± 26	153 ± 9	623 ± 134	72 ± 8

Borussertib	191 ± 90	48 ± 15	5 ± 1	277 ± 90	373 ± 54	7770 ± 641
**24a**	159 ± 70	25 ± 7	2 ± 0	138 ± 81	259 ± 68	2313 ± 908
**24b**	230 ± 75	11 ± 2	2 ± 0	32 ± 8	109 ± 16	7157 ± 2112
**24c**	15 333 ± 3264	2114 ± 49	427 ± 23	20 267 ± 7039	11 971 ± 1636	> 30 000
**27**	2063 ± 438	336 ± 25	39 ± 3	1111 ± 117	1785 ± 458	1660 ± 46
**30a**	1328 ± 60	41 ± 19	22 ± 7	1024 ± 621	2268 ± 700	5543 ± 1506
**30b**	382 ± 31	95 ± 20	11 ± 3	336 ± 113	464 ± 31	14 942 ± 5541
**30c**	3467 ± 1693	1359 ± 454	151 ± 47	2841 ± 685	5211 ± 1892	11 332 ± 4428
**30d**	1311 ± 158	546 ± 87	73 ± 25	2437 ± 1017	1956 ± 391	6067 ± 1104
**30e**	568 ± 106	179 ± 30	10 ± 3	224 ± 88	621 ± 85	6442 ± 1238
**30f**	879 ± 223	183 ± 52	33 ± 9	1190 ± 518	1104 ± 212	3055 ± 848
**31**	3614 ± 1292	832 ± 81	116 ± 16	1218 ± 58	4617 ± 1059	17 119 ± 6379
**32**	3288 ± 298	1897 ± 578	384 ± 62	2196 ± 125	4019 ± 912	6755 ± 1462

^*a*^Genetic alterations of the evaluated cancer cell lines according to COSMIC database.

In addition, small substitutions revealed by **30e** or bioisosteric scaffolds as shown for **30f** are well tolerated, and correspond well to their biochemical profile. In summary, out of a small focused library, a set of inhibitors was identified having an excellent biochemical and cellular activity. In order to ascertain the best candidates for *in vivo* characterization and lead development, we further investigated a selection of promising inhibitors concerning pharmacokinetic properties.

### Evaluation of phase I metabolic stability

The cellular evaluation demonstrated high inhibitory activity of a series of covalent-allosteric Akt inhibitors on different cancer cell lines, thus making these molecules potential candidates for further preclinical investigation. To understand subtle differences between the best inhibitors and to assess their ability to examine them in subsequent *in vivo* studies, we further analyzed a representative selection of inhibitors with respect to their pharmacokinetic properties (Table 3). For this purpose, we used a microsomal stability assay that is a good predictor for phase I metabolic stability.^50^ As discussed in previous studies, borussertib revealed a good pharmacokinetic profile with reasonable microsomal stability in human and murine microsomes.^29^ Inhibitor **24a**, which exhibited an equivalent activity compared to borussertib in cellular evaluations, showed similar stability properties. Combining the superior cellular activity and comparable stability, **24a** is a promising candidate with optimized potency for further *in vivo* investigation. In contrast, 4-fold lower stability in microsomes occurred for **24b**, thus the methyl group seems to be a metabolically labile position of the inhibitor. A similar loss in stability was observed for phenylurea-derived inhibitor **27**. Therefore, a more detailed analysis of the metabolic hot spots of this inhibitor is required to enable the chemical optimization of its pharmacokinetic properties. The phenylamide-based probe molecules also showed good microsomal stability, whereas **30b** stuck out with a 4-fold longer half-life in murine microsomes. Consequently, with **30b** another covalent-allosteric inhibitor of protein kinase Akt could be identified with optimized stability for *in vivo* mouse studies without a decrease of activity as validated in biochemical and cellular evaluation.

**Table 3:** Evaluation of covalent-allosteric Akt inhibitors in a microsomal stability assay

	Microsomal stability phase I	
	*t*_1/2_ [min] (H^*a*^/M^*b*^)	Cl_int_ [μL min^–1^ mg^–1^] (H^*a*^/M^*b*^)
Capivasertib	33/87	14/16
Ipatasertib	39/231	12/6
MK-2206	347/693	1/2
Borussertib	99/46	5/30
**24a**	87/41	5/34
**24b**	25/23	19/60
**27**	19/20	24/68
**30b**	58/173	8/8
**30e**	39/69	12/20
**30f**	39/58	12/24

^*a*^Human liver microsomes.

^*b*^Murine liver microsomes.

## Discussion

**Fig. 4:**
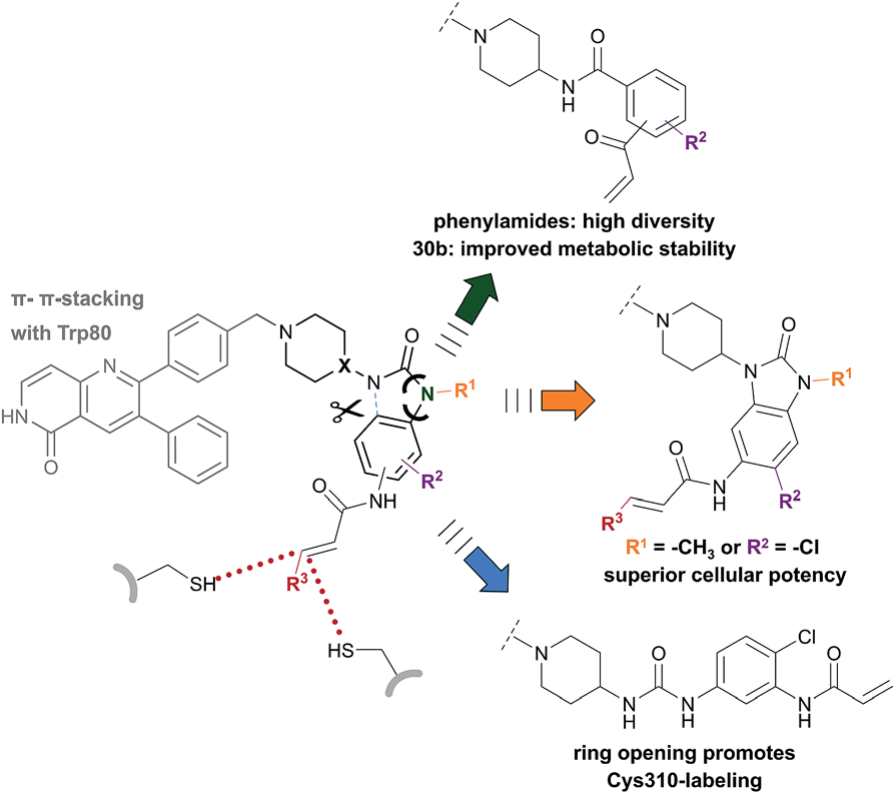
Overview illustrates the synthesized and evaluated library of covalent-allosteric Akt inhibitors. Phenylamide-, benzo[*d*]imidazolone- and urea-based derivates gain insights into the structure–activity-relationships. The diversification of scaffolds and resulted in novel binding modes with different preference in labeling Cys296 and Cys310. Phenylamide-based inhibitors broadened chemical space and showed improved metabolic stability for **30b**. Small substitutions of the benzo[*d*]imidazolone-core allowed a gain of cellular potency. Urea-based inhibitor **27** revealed a novel binding mode by promoting Cys310-modification.

By combining the beneficial properties of allosteric and irreversible inhibitors, we developed covalent-allosteric Akt inhibitors which represent a novel class of probe molecules with outstanding selectivity and potency profiles. The structural information emanating from the recently published first crystal structure of a covalent-allosteric Akt inhibitor borussertib inspired a series of structure-based designed inhibitors that acquire deeper insights into the structure–activity relationships (Fig. 4). The established synthetic route enabled us to access diverse derivatives that could be analyzed concerning their biochemical and cellular characteristics. Through biological and pharmacokinetic evaluations, we were able to evaluate these compounds regarding their inhibitory efficacy and ability to act as eligible candidates for further preclinical investigations. The structural evaluation revealed that π–π-interactions of the 1,6-naphthyridinone scaffold with Trp80 were of great importance for binding affinity, and thus the core scaffold was maintained in the design of the novel compounds. On the other side of the molecule, the benzo[*d*]imidazolone moiety allows an optimal orientation of the acrylamide warhead towards the covalently targeted cysteine residues. Consistent with the complex structure, small substituents in the 5-position of this scaffold were well tolerated, and, subsequently, the chlorine substitution in molecule **24a** had a positive influence on the cellular efficacy with a reasonable pharmacokinetic profile. Furthermore, the unoccupied polar narrow pocket in the direction of the free amide of the imidazolone ring was of high interest in the design of novel probe molecules. N-Methylation at this position led to **24b** with a methyl group pointing toward this area with increased cellular potency in comparison to borussertib. Although the metabolic stability assay indicated a metabolic hot spot at this position, further investigations concerning a presumable cleavage of the methyl group in phase I metabolism should specify a potential role of **24b** as a prodrug concept for borussertib. The complex crystal structure of **24b** in complex with Akt showed a novel alternative binding mode with labeling of the adjacent Cys310 instead of Cys296, while the absence of polar interactions towards this conserved water molecule induced an altered conformation of the piperidine ring. The influence of more polar substituents at this position on the distinct binding mode should contribute to deeper understanding and further optimization. However, the ability to label both cysteine residues could have a beneficial effect regarding resistance mutations of these amino acids that are known to occur for other cysteine-modifying covalent kinase inhibitors in cancer treatment.^51^ In order to allow access to more substituents occupying higher chemical space, the benzo[*d*]imidazolone core was substituted by ring opening to afford phenylurea-based compounds. The closely related structure of both scaffolds suggested that this replacement was well tolerated. The slight increase in half-maximal inhibitory concentration for **27** could be attributed to the higher entropic degrees of freedom induced by the ring opening, leading to a higher entropic penalty upon binding to the kinase. By solving the complex crystal structure of **27**, we also revealed the altered binding mode by labeling Cys310, which is promoted by the elongated structure of **27**. Given the lower microsomal stability, the urea-based inhibitor still has a need for further optimization. However, they represent an interesting novel class of probe molecules. In addition, phenylamide-based derivatives constituted another novel type of covalent allosteric Akt inhibitors. A series of derivatives verified the possibility to introduce different small substituents as shown for the halide-derivatives **30d** and **30e**, and bioisosteric variants as shown for **30f** are well tolerated. The biochemical evaluation of these compounds showed a good correlation to the inhibitory effect on different cancer cell lines. In particular, this aspect became apparent in comparing the *ortho*-, *meta*- and *para*-derivatives **30a–c**. These probe molecules additionally confirmed the importance of an optimal preorganization of the acrylamide warhead toward the targeted cysteine for covalent bond formation, also manifested by the co-crystal structure of Akt in complex with **30b**. The shortened molecule length promoted labeling of Cys296, also illustrated by the complex crystal structure of **31**. Probe molecule **30b** stood out having high nanomolar cellular activity and additionally an improved pharmacokinetic profile in comparison to borussertib due to better microsomal stability.

## Conclusions

In summary, we have validated the concept of covalent-allosteric inhibitors as highly potent modulators of the protein kinase Akt by a structure-guided design of a small focused library of novel probe molecules. Phenylurea- and phenylamide-based inhibitors opened a broader chemical space for further derivatization and optimization and led to a synthesis route that allows access to a variety of novel derivatives. Biochemical characterization, kinetic parameters, and cellular evaluation provided a detailed view of the structure–activity relationships of covalent-allosteric inhibitors. By further analyzing the microsomal stability, the inhibitors **24a** and **30b** were identified as potent and stable novel candidates for *in vivo* studies and lead development.

## Statement of contributions

N. U. and S. S. contributed equally and designed and carried out the synthesis. J. W. performed the activity-based assay and kinetic experiments. I. L., M. P. M. and R. S. solved the crystal structures. M. L., R. G., L. D. and L. Q. contributed to synthesis. J. W. and I. L. conducted the cell viability assay. T. A. L. and B. E. performed the molecular dynamic simulations. J. H. determined the microsomal stability. P. C. carried out the molecular modeling studies. P. J. performed MS/MS studies. N. U., S. S. and M. L. wrote the manuscript and all authors have given approval to the final version. D. R. conceived and designed all experiments.

## Conflicts of interest

There are no conflicts to declare.

## Supplementary Material

Supplementary information
