# Supplementary material for: Structural and chemical insights into the covalent-allosteric inhibition of the protein kinase Akt
Source: Chem Sci. 2019 Feb 13;10(12):3573–85. doi: 10.1039/c8sc05212c (PMC6430017; doi:10.1039/c8sc05212c)
Supplement: Supplementary file 1 [file SC-010-C8SC05212C-s001.pdf]

Electronic Supplementary Material (ESI) for Chemical Science.
This journal is © The Royal Society of Chemistry 2019

Structural and Chemical Insights into the Covalent-allosteric Inhibition of the Protein Kinase Akt

Authors: Niklas Uhlenbrock, Steven Smith, Jörn Weisner, Ina Landel, Marius Lindemann, Thien Anh Le, Julia Hardick, Rajesh Gontla, Rebekka Scheinpflug, Paul Czodrowski, Petra Janning, Laura Depta, Lena Quambusch, Matthias P. Müller, Bernd Engels and Daniel Rauh

Supplementary Information

Table of Contents

Experimental Section **S. 3-9**

Synthetic procedures and compound characterization **S. 10-56**

Figure S1 Synthetic scheme for synthesis of benzo[*d*]imidazolone-based covalent-allosteric inhibitors **S. 18**

Figure S2 Synthetic scheme for the synthesis of **31** **S. 51**

Figure S3 Synthetic scheme for the synthesis of **32** **S. 53**

Figure S4-S16 NMR spectra . **S. 57-69**

Table S1. Data collection and refinement statistics for full-length Akt1 in complex with different covalent-allosteric Akt Inhibitors **S. 70**

Figure S17 mFo-DFc maps **S. 71**

Figure S18 Modeling of novel Akt inhibitors **S. 72**

Table S2-4 Kinase Profiling **S. 73-75**

Figure S18 Kinase Profiling Kinome Dendogram **S. 76**

Figure S19 Protein-Ligand-Interactions **S. 77**

Figure S20 MS-MS analysis of covalent-allosteric Akt inhibitor **27** **S. 78**

Figure S21-23 MD-calculations **S. 79-80**

Figure S24-25 Metabolic stability **S. 81**

References **S. 82-84**

Experimental Section

Chemistry

All reagents and solvents were purchased from Acros, Activate Scientific, Alfa Aesar, Apollo Scientific, Merck, Sigma-Aldrich, TCI Chemicals or VWR and used without further purification. Dry solvents were purchased as anhydrous reagents from commercial suppliers. ^{1}H and ^{13}C NMR spectra were recorded on a Bruker Avance DRX AV400 (400 MHz and 101 MHz), AV500 (500 MHz and 125 MHz), AV600 (600 MHz and 151 MHz) and AV700 (700 MHz and 176 MHz). ^{1}H chemical shifts are reported in δ (ppm) as s (singlet), d (doublet), dd (doublet of doublet), t (triplet), q (quartet), m (multiplet) and b (broad singlet) and are referenced to the residual solvent signal: $CDCl_3$ (7.26), DMSO-d_6 (2.50) or MeOD-d_4 (3.34). ^{13}C spectra are referenced to residual solvent signal: $CDCl_3$ (77.1), DMSO-d_6 (39.52) or MeOD-d_4 (49.86). High-resolution electrospray ionization mass spectra (ESI-FTMS) were recorded on a Thermo LTQ Orbitrap (high-resolution mass spectrometer from Thermo Electron) coupled to an Accela HPLC system supplied with a Hypersil GOLD column (Thermo Electron). LCMS (ESI-MS) analysis was performed using Agilent HPLC system (1100 series) with CC 125/4 Nucleodur C18 gravity column (3 µm) from Macherey Nagel coupled to a Thermo Scientific Finnigan LCQ Advantage Max Ion Trap and ESA Corona detector. Analytical TLC was carried out on Merck 60 F254 aluminium-backed silica gel plates. Compounds were purified by column chromatography using VWR silica gel (40 - 63 µm particle size) or flash chromatography on a Biotage Isolera One using Büchi Reveleris Silica Cartridges (4 - 120 g) monitored by UV at λ = 210 nm and 280 nm. Preparative HPLC was conducted on an Agilent HPLC system (1200 series) with a VP 125/21 Nucleodur C18 column from Macherey-Nagel and monitored by UV at λ = 210 nm and 254 nm. All final compounds were purified to > 95 % purity as determined by high-performance liquid chromatography (HPLC). Purity was measured using Agilent 1200 series HPLC systems with UV detection at λ = 210 nm (system: Agilent Eclipse XDB-C18 4.6 mm x 150 mm, 5 µM, 10 – 100 % MeCN in H_2O, with 0.2 % TFA, for 15 min at 1.0 mL/min).

Activity-based Assay for IC_{50}-Determination and Kinetic Characterization

The biochemical inhibitory activity (IC_{50}) was determined with the HTRF KinEASE assay from Cisbio as previously described.[1] All reagents for HTRF experiments were purchased from Cisbio Bioassays, France. The measurements were analyzed using the Quattro Software Suite for IC_{50}-determination. Time-dependent IC_{50}-measurements were performed as described. Briefly, IC_{50} values were determined for twelve different incubation times and afterwards plotted versus accordingly. Data was analyzed according to literature procedure.[2]

Mass spectrometry

We used Akt1 for MS experiments and incubated 10 µM of the protein with 100 µM of the inhibitor in a buffer for 1 h. We analyzed the samples by mass spectrometry using a Thermo Fisher Scientific Ultimate 3000 HPLC system connected with a Thermo Fisher Scientific Velos Pro (2D ion trap). The sample (5 µL) was injected and separated by a Vydac 214TP C4 5 µm column (150 mm x 2.1 mm) starting at 20 % Acetonitrile/0.1 % FA in water/0.1 % FA for 5 min, followed by a linear gradient over 14 min up to 90 % Acetonitrile/0.1 % FA in water/0.1 % FA. A mass range of 700-2000 m/z was scanned and raw data were deconvoluted and analyzed with ProMass. The deconvoluted mass spectra were smoothed and cropped to a mass range of 59,000 – 60,300 m/z with the software mMass (version 5.5.0).[3]

For nanoESI-MS/MS analysis of **27**, the sample was denaturated, separated via SDS-PAGE standard tryptic in-gel digest protocols. Subsequently, the sample was thawed, dissolved in 20 µL of 0.1 % TFA in water, sonicated at room temperature for 15 min, and centrifuged at 15,000 x g for 1 min shortly before analysis. 3 µL of sample were loaded onto a pre-column cartridge and desalted for 5 min using 0.1 % TFA in water as eluent at a flow rate of 30 µL/min. The sample was back-flushed from the pre-column to the nano-HPLC column during the whole analysis. Elution was performed using a gradient starting from 5 % solvent B increasing to 30 % B after 35 min with a flow rate of 300 nL/min using 0.1 % FA in water as solvent A and 0.1 % FA in MeCN as solvent B and a column temperature of 40 °C. The nano-HPLC column was washed by increasing the percentage of solvent B to 60 % in 5 min and to 95 %

in additional 5 min, washing the columns for further 5 min, flushing back to starting conditions and equilibration of the system for 14 min. During the complete gradient cycle, a typical TOP10 shot-gun proteomics method for the MS and MS/MS analysis was used. For full scan MS experiments a mass range of m/z 300 to 1650 was scanned with a resolution of 70,000. MS/MS scans with a resolution of 17,500 of the most intense at least doubly charged ions. Data evaluation was perfomed using MaxQuant.[4] Spectra were searched against the Akt1-sequence and a contamination database using a false discovery rate of 1 % on peptide and protein level using a decoy database for determination of the false discovery rate. For database search oxidation of methionine and N-terminal acetylation of proteins, carbamidomethylation of cysteines, and artificial modification of cysteines were defined as variable modifications.

Protein Expression, Purification, and Crystallization

A gene encoding for Akt1(2-446, E114/115/116A) including an N-terminal His6-Tag followed by a TEV protease recognition site was synthesized by GeneArt AG (Regensburg, Germany) and cloned into the pIEx/Bac3 expression vector (Merck Millipore) using NcoI and BamHI restriction sites. Transfection, virus generation, and amplification as well as protein expression were carried out in Spodoptera frugiperda (Sf9) cells (ThermoScientific) following the BacMagic protocol (Merck Millipore). Infected insect cells were grown in Erlenmeyer flasks for 72 hours at 27 °C with shaking at 120 rpm, subsequently harvested by centrifugation at 3,000 x g for 15 min and washed once with PBS before being flash frozen in liquid nitrogen. Afterwards, cells were thawed and resuspended in lysis buffer (50 mM Tris, 500 mM NaCl, 1 mM DTT, 10 % glycerol, 0.1 % Triton X-100, pH 8.0, EDTA-free protease inhibitor cocktail (Sigma)). Cells were lysed using a microfluidizer, the lysate was cleared by centrifugation (40,000 x g, 1 h). The supernatant was loaded onto a Ni-NTA Superflow cartridge (Qiagen). Bound protein was eluted in buffer containing 50 mM Tris, 500 mM NaCl, 500 mM imidazole, 1 mM DTT, 10 % glycerol, pH 8.0. For cleavage of the hexahistidine-tag, TEV protease was added to the pooled elution fractions and dialyzed overnight into buffer containing 25 mM Tris, 50 mM NaCl, 1 mM DTT, 5 % glycerol, pH 8.0 at 4 °C. The cleaved protein was further purified by anion-exchange

chromatography using a HiTrap Q HP column (GE Healthcare) followed by size-exclusion chromatography on a HiLoad 16/60 Superdex 75 pg column (GE Healthcare) using buffer containing 50 mM HEPES, 200 mM NaCl, 1 mM DTT, 10% glycerol, pH 7.3. Afterwards, the protein was transferred into the storage buffer (25 mM Tris, 100 mM NaCl, 1 mM DTT, 10 % glycerol, pH 7.5) using a Superdex 75 10/300 GL column (GE Healthcare), concentrated and stored at -80 °C.

For crystallization, purified protein at a concentration of 3 mg/mL was incubated with 3 eq. of every inhibitor on ice for 60 min. The samples were centrifuged at 20,000 x g for 10 min before hanging drops were prepared in 15-well crystallization plates (EasyXtal Tool, Qiagen) by mixing protein-ligand complex with reservoir solution (1:1) containing 1.25 mM sodium acetate pH 5.2, 3.75 mM sodium citrate pH 5.2, 15 % PEG MME 2000 at 20 °C. Diffraction-grade crystals grew within 3 days and were cryoprotected using 20 % ethylene glycol before they were flash cooled in liquid nitrogen. X-ray diffraction data were collected at the PXII X10SA beam line of the Swiss Light Source (PSI, Villingen, Switzerland) with wavelengths close to 1 Å. The diffraction data were integrated with XDS and scaled using the program XSCALE.[5] The crystal structure was solved by molecular replacement with PHASER using an unpublished co-crystal structure of Akt1 in complex with another covalent-allosteric inhibitor as template.[6] The manual modification of the molecule of the asymmetric unit was performed using the program COOT[7] and with the help of the Dundee PRODRG server the inhibitor topology files were generated.[8] For multiple cycles of refinement PHENIX.refine[9] was employed and the final structure was evaluated by Ramachandran plot analysis using MolProbity.[10] Final validation of the model was performed with help of the PDB_REDOserver[11] and crystal structures were visualized using PyMOL (See also Supplement Fig. 17).[12]

Molecular Dynamic simulations

For the MD simulations, the missing amino acids of all analyzed complex crystal structures were added by the program package Modeller 9.18. For each crystal structure we obtained three completed models which we used in the subsequent simulations. Due to a similar enzyme environment it can be expected that the reactivity of both cysteine residues are very similar. Hence, their relative reaction

probabilities correlate with the distance of the respective sulfur center with the electrophilic carbon center of the warhead in the non-covalent enzyme-inhibitor complex, i.e. before the reaction. To estimate theses distances, we started from the experimental crystal structures reflecting the covalent enzyme-inhibitor complex (after the reaction) and prepared the situation within the non-covalent complex (before the reaction), i.e. we changed the geometrical parameters of the inhibitor to those of the unreacted inhibitor and restored the respective Cys residue. In the following we performed preparatory MD simulation to adapt the enzyme environment taken from the experimental crystal structure to the new situation of the non-covalent enzyme inhibitor complex.All subsequent MD simulations for the non-covalent complex have been performed using the AMBER14 program package.[13] In these simulations the protein has been described by the AMBER force field ff14SB[14] while the inhibitors **1, 24b** and **27** were parameterized in noncovalent complex by utilizing gaff (generalized AMBER forcefield).[15] Electro-static potential (ESP) charges of inhibitors **1**, **24b** and **27** were calculated with the Gaussian03 program package[16] on Hartree Fock level employing the 6-31G(d) basis set.[17-18] Each protein-inhibitor complex has been solvated with the TIP3P water model in an octahedral shell.[19] To adapt the enzyme environment to the situation before the reaction we used the following procedure. After a minimization of 1000 cycles for the solvent with restraints on the protein-ligand complex, further 2500 cycles of minimization have been performed for the entire system without any restraints. Using these optimized structures, MD simulations with varying temperatures were performed. After a gradual heating to 300 K with a simulation duration of 100 ps keeping restraints on the non-aqueous part, the simulation was continued for 50 ns at constant temperature (300 K) and pressure (1 atm). A time step of 1 fs was chosen. Bonds involving hydrogen were constrained with the SHAKE algorithm and simulations were conducted with periodic boundary conditions.[20-21] After the preparation of the system we performed a 50 ns production run, and plotted the variation of relevant distances (e.g. distance between the sulfur centers of Cys296 and Cys310 and the attacked carbon atom) as a function of simulation time. The data are summarized in Figure S19-21.

Cell Culture and Viability Assay

Source and cultivation of all tested cell lines was described in detail elsewere.[22] For cell viability analysis, cells were plated on day 0 into white 384-well cell culture plates (Greiner Bio-One) using a Multidrop reagent dispenser (Thermo) at cell numbers that ensure linear and optimal luminescent signal intensity (AN3-CA: 800 cells/well; BT-474: 400 cells/well; Dan-G: 400 cells/well; HPAF-II: 400 cells/well; KU-19-19: 400 cells/well; MCF-7: 200 cells/well; T-47D: 800 cells/well; ZR-75-1: 400 cells/well). Then the cells were incubated for 24 h in a humidified atmosphere at 37 °C/5 % CO_2. The cells were treated with inhibitors in serial dilutions ranging from 30 µM down to 0.1 nM using an Echo 520 acoustic liquid handler (Labcyte Inc.). Cell viability was analysed on day 5 using the CellTiter-Glo assay (Promega) as per manufacturer's instructions. Luminescence signal was recorded by an EnVision Multilabel 2104 Plate Reader (PerkinElmer) with 500 ms integration time. All data were normalized to the plate positive control (30 µM staurosporine) and negative control (DMSO). Finally, the data was processed with the Quattro Software Suite using a four parameter logistic model. As a quality control, the Z'-factor was calculated from 16 positive and negative control values. Only assay results showing a Z'-factor ≥ 0.5 were used for further analysis. All experimental points were measured in duplicates for each plate and were replicated in at least three times.

Phase I metabolic stability assay

Metabolic stability under oxidative conditions was determined by LCMS-based measuring of degradation of compounds at 3 µM in human and murine liver microsomes over 60 minutes at 37 °C (Supplement Fig. S22-23). Calculating the compound half-life $t_{1/2}$, the *in vitro* intrinsic clearance CL_{int} is given.

Data Availability

The structures of Akt1 in complex with the inhibitors **24b**, **27**, **30b** and **31** have been deposited in the Protein Data Bank under PDB-ID 6HHJ for **24b**, PDB-ID 6HHG for **27**, PDB-ID 6HHI for **30b** and PDB-ID 6HHH for **31**. 3D structural models for Augment are also available via QR-codes within the corresponding figures.[23]

Synthetic procedures and compound characterization

Common Procedure A: Nitration of benzo[d]imidazolone derivatives

1 eq. Benzo[*d*]imidazolone was stirred at 60 °C in 100 mL o-xylene. Then, 6 eq. of concentrated nitric acid (69 %) were added dropwise and the reaction mixture was heated for 2 h. The solvent was decanted and the residual crude product was washed with MeOH. Afterwards, the pure product was filtered off.

Common Procedure B: Selective Boc-Protection of anilinic amine

1 eq. amine was stirred in 60 mL 10 % acetic acid in H_2O. 2 eq. Boc_2O dissolved in 10 mL 1,4-dioxane was added dropwise and the reaction mixture was stirred overnight at room temperature. The reaction mixture was basified with 10 M NaOH and extracted with 10 % MeOH in DCM. The combined organic fractions were dried over Na_2SO_4 and evaporated *in vacuo*. Silica gel column chromatography (7 - 10 % MeOH/DCM + 1 % NH_3) yielded the final product.

Common Procedure C: Amide coupling of benzoic acid derivatives

To a solution of 1 eq. carboxylic acid derivatives in DMF (0.2 M) 1 eq. HATU and 3 eq. 2,6-lutidine were added. Subsequently, 1 eq. *N*-Boc-4-aminopiperidine was added and the reaction mixture was stirred at room temperature overnight under argon atmosphere. Following, water was added until the product precipitated. The suspension was stirred for 30 min at room temperature and the precipitate was filtered off and washed with water. The desired product was dried *in vacuo* and used without further purification.

Common Procedure D: Nitro-reduction with iron powder

To a solution of 1 eq. nitro derivatives in MeOH/water (9:1), 6 eq. iron powder, and 9 eq. NH_4Cl were added and the reaction mixture was stirred for 2 h at 80 °C. The suspension was filtered over Celite,

the solvent was evaporated *in vacuo* and the crude product was purified via silica gel column chromatography (7 - 10 % MeOH/DCM + 1 % NH_3).

Common Procedure E: Leuckart-Wallach reaction

To a solution of 1.0 eq. aldehyde and 1.1 eq secondary amine in 10 mL MeCN 4.0 eq. formic acid was added and the reaction mixture was stirred at 80 °C overnight. Subsequently, the reaction mixture was cooled to room temperature, the solvent was evaporated *in vacuo* and silica gel column chromatography yielded the desired product (1 - 10 % MeOH/DCM + 1 % NH_3).

Common Procedure F: Boc deprotection

A solution of 1.0 eq. boc-protected amine was stirred in 10 mL HCl in 1,4-dioxane (4 N) for 2 h at room temperature. After completion, the reaction mixture was basified with 10 M NaOH solution. The resulting precipitate was separated and extracted with dichloromethane. The combined organic fractions were dried over Na_2SO_4 and the solvent was evaporated *in vacuo*. The pure product was obtained after silica gel column chromatography (7 - 10 % MeOH/DCM + 1 % NH_3).

Common Procedure G: Acrylamide coupling

1.0 eq. secondary amine was dissolved in 4 mL THF and 10 eq. DIPEA and was stirred for 15 min at 0 °C under argon atmosphere. Subsequently, a solution of 1.1 eq acryloyl chloride in 1 mL THF was added dropwise. The reaction mixture was stirred at room temperature until completion. The reaction was quenched a saturated solution of $NaHCO_3$. The aqueous layer was extracted with ethyl acetate and the combined organic layers were washed with distilled water and saturated NaCl solution and dried over Na_2SO_4. The solvent was evaporated *in vacuo* and silica gel column chromatography yielded the desired product (7 - 10 % MeOH/DCM + 1 % NH_3).

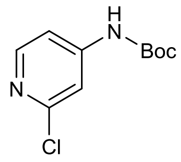

3

Synthesis of tert-butyl (2-chloropyridin-4-yl)carbamate (**3**)**.** To a solution of 2-chloropyridin-4-amine (**2**, 25 g, 194.46 mmol, 1 eq.), DMAP (7.1 g, 58.12 mmol, 0.30 eq.) and triethylamine (67 mL, 486.15 mmol, 2.5 eq) in 200 mL DCM di-*tert*-butyl dicarbonate (44.7 mL, 194.46 mmol, 1 eq.) was added. The reaction mixture was stirred at room temperature for 2 h and after completion quenched with a sat. NH_4Cl solution and extracted with ethyl acetate. The combined organic fractions were washed with water and a sat. NaCl solution and dried over Na_2SO_4. The solvent was evaporated *in vacuo* and silica gel column chromatograhphy (20 % EtOAc/PE) yielded 23.7 g (103.4 mmol, 53 %) of the desired product. **^{1}H-NMR (500 MHz, DMSO-*d*$_6$)** δ 10.12 (s, 1H), 8.17 (d, *J* = 5.7 Hz, 1H), 7.55 (d, *J* = 1.5 Hz, 1H), 7.36 (dd, *J* = 5.7 Hz, 1.6 Hz, 1H), 1.48 (s, 9H). **^{13}C-NMR (126 MHz, DMSO-*d*$_6$)** δ 152.63, 151.37, 150.55, 149.52, 112.16, 111.69, 81.22, 28.33. **LCMS (*m/z*)** calcd.: for $C_{10}H_{13}ClN_2O_2$ $[M+H]^+$, 229.07; found: 228.80.

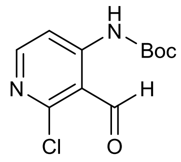

4

Synthesis of tert-butyl (2-chloro-3-formylpyridin-4-yl)carbamate (**4**)**.** *Tert*-butyl (2-chloropyridin-4-yl)carbamate (**3**, 4 g, 17.5 mmol, 1 eq.) was dissolved in THF (60 mL) and cooled down to -78 °C. Under argon atmosphere, *tert*-butyl lithium (29.8 mL, 50.7 mmol, 2.9 eq.) was added dropwise and the reaction mixture was stirred for 2 h at -78 °C. After 2 h DMF (8.1 mL, 105 mmol, 6 eq.) was added dropwise and the reaction mixture was allowed to warm up to room temperature overnight. The reaction mixture was subsequently quenched with a sat. NH_4Cl solution and extracted with ethyl acetate. The combined organic fractions were washed with water and sat. NaCl solution and dried over Na_2SO_4. The solvent was evaporated *in vacuo* and silica gel column chromatography (25 % EtOAc/PE)

yielded 3.9 g (15.1 mmol, 86 %) of the desired product. **^{1}H-NMR (400 MHz, DMSO-*d*$_6$)** δ 10.93 (s, 1H), 10.33 (s, 1H), 8.42 (d, *J* = 6 Hz, 1H), 8.23 (d, *J* = 6 Hz, 1H), 1.51 (s, 9H). **^{13}C-NMR (101 MHz, DMSO-*d*$_6$)** δ 194.78, 155.75, 154.43, 151.59, 113.65, 112.35, 82.85, 28.11. **LCMS (*m/z*)** calcd.: for $C_{11}H_{13}ClN_2O_3$ $[M+H]^+$, 257.06; found: 256.82.

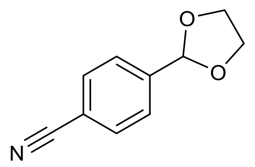

6

Synthesis of 4-(1,3-dioxolan-2-yl)benzonitrile (**6**)**.** A mixture of 4-formylbenzonitrile (**5**, 30 g, 228.8 mmol, 1 eq.), ethylene glycol (51.6 mL, 915.1 mmol, 4 eq.) and *p*-toluenesulfonic acid (1.3 g, 6.9 mmol, 0.03 eq.) in 300 mL was refluxed with a dean-stark trap overnight. The reaction mixture was quenched with sat. NaCl solution and extracted with ethyl acetate. The combined organic phases were washed with water and sat. NaCl solution and dried over Na_2SO_4. The solvent was evaporated *in vacuo* and the resulting oily liquid was stored at 4 °C for crystallization. The excess solvent was removed in the high vacuum and 30.4 g (173.5 mmol, 76 %) of the desired product was received. **^{1}H NMR (500 MHz, $CDCl_3$)** δ 7.68 (d, *J* = 8.4 Hz, 2H), 7.59 (d, *J* = 8.2 Hz, 2H), 5.85 (s, 1H), 4.13 – 4.02 (m, 4H). **^{13}C NMR (126 MHz, $CDCl_3$)** δ 143.26, 132.37, 127.32, 118.73, 113.10, 102.61, 65.60. **LCMS (*m/z*)** calcd.: for $C_{10}H_9NO_2$ $[M+H]^+$, 176.07; not ionisable.

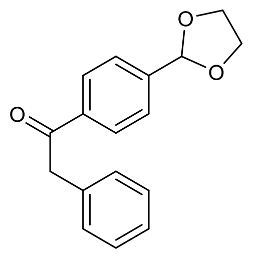

7

Synthesis of 1-(4-(1,3-dioxolan-2-yl)phenyl)-2-phenylethan-1-one (**7**)**.** Under argon atmosphere a solution *of* 4-(1,3-dioxolan-2-yl)benzonitrile (**6**, 4 g, 22.8 mmol, 1 eq.) in dTHF was cooled to -4 °C and benzylmagnesium chloride (40 mL, 79.9 mmol, 3.5 eq.) was added dropwise. After stirring for 1 h the

reaction mixture was warmed up to room temperature and stirred for 4 h. The reaction mixture was quenched with sat. NH_4Cl solution and the resulting salt was filtered off and the solvent was removed *in vacuo*. Silica gel column chromatography (30 % EtOAc/PE) yielded 3.9 g (14.5 mmol, 64 %) of the desired product. **^{1}H-NMR (400 MHz, DMSO-*d*$_6$)** δ 8.07 (d, *J* = 8.3 Hz, 2H), 7.58 (d, *J* = 8.3 Hz, 2H), 7.21-7.33 (m, 5H), 5.82 (s, 1H), 4.40 (s, 2H), 4.01-4.07 (m, 2H), 3.95-4.01 (m, 2H). **^{13}C-NMR (101 MHz, DMSO-*d*$_6$)** δ 197.88, 143.52, 137.34, 135.5, 130.18, 128.86, 127.31, 126.98, 102.52, 65.42, 45.28. **LCMS (*m/z*)** calcd.: for $C_{17}H_{16}O_3$ [M+H]$^+$, 269.12; found: 269.05.

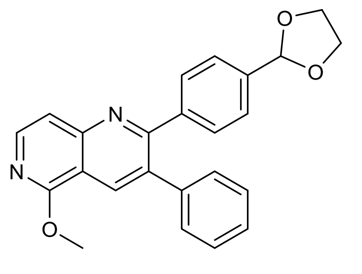

8

Synthesis of 2-(4-(1,3-dioxolan-2-yl)phenyl)-5-methoxy-3-phenyl-1,6-naphthyridine (**8**)**.** To a solution of *tert*-butyl-(2-chloro-3-formylpyridine-4-yl)carbamate (**4**, 4 g, 15.6 mmol, 1 eq.) and 1-(4-(1,3-dioxolane-2-yl)phenyl)-2-phenylethane-1-one (**7**, 4.3 g, 16.1 mmol, 1.03 eq.) in 60 mL dMeOH sodium methoxide (30 % in MeOH, 4.7 mL, 23.4 mmol, 1.5 eq.) was added dropwise. The reaction was stirred for 4 h at 65 °C and subsequently the solvent was evaporated *in vacuo*. The resulting residue was dissolved in ethyl acetate and extracted with sat. $NaHCO_3$ solution. The organic phases were washed with water and sat. NaCl solution and dried over Na_2SO_4. The solvent was evaporated *in vacuo* and silica gel column chromatography (10 % MeOH/DCM) yielded 3.8 g (9.9 mmol, 63 %) of the desired product. **^{1}H NMR (500 MHz, DMSO-*d*$_6$)** δ 8.41 (s, 1H), 8.27 (t, *J* = 5.4 Hz, 1H), 7.51 (t, *J* = 5.1 Hz, 1H), 7.43 – 7.38 (m, 2H), 7.37 – 7.31 (m, 5H), 7.26 (dd, *J* = 6.9 Hz, 2.5 Hz, 2H), 5.71 (s, 1H), 4.10 (s, 3H), 4.04 (dd, *J* = 8.7 Hz, 5.1 Hz, 2H), 3.95 – 3.90 (m, 2H). **^{13}C NMR (126 MHz, DMSO-*d*$_6$)** δ 161.72, 161.10, 151.51, 144.42, 140.65, 139.35, 138.77, 135.33, 134.18, 130.22, 129.94, 128.96, 128.11, 126.60, 116.28, 113.53, 102.91, 65.33, 54.60. **LCMS (*m/z*)** calcd.: for $C_{24}H_{20}N_2O_3$ [M+H]$^+$, 385.15; found: 385.24.

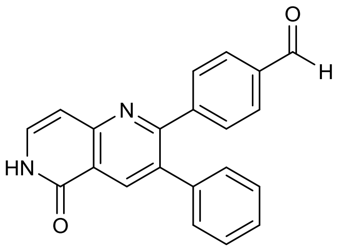

9

Synthesis of 4-(5-oxo-3-phenyl-5,6-dihydro-1,6-naphthyridin-2-yl)benzaldehyde (**9**)**.** 3 g of 2-(4-(1,3-dioxolan-2-yl)phenyl)-5-methoxy-3-phenyl-1,6-naphthyridine (**8**, 7.8 mmol, 1 eq.) in 200 mL aqueous HCl (37 %) were heated to 90 °C for 3 h. Subsequently, the reaction mixture was cooled down to room temperature and adjusted to pH > 7 with 10 M NaOH solution. The resulting precipitate was filtered off and washed with cool water. 2.3 g yellow-orange colored product (7.1 mmol, 91 %) were received. **^{1}H NMR (600 MHz, DMSO-*d*$_6$)** δ 11.66 (s, 1H), 10.00 (s, 1H), 8.44 (s, 1H), 7.83 (d, *J* = 8.2 Hz, 2H), 7.57 (d, *J* = 8.2 Hz, 2H), 7.52 (d, *J* = 7.3 Hz, 1H), 7.35 – 7.31 (m, 3H), 7.27 – 7.23 (m, 2H), 6.71 (d, *J* = 7.3 Hz, 1H). **^{13}C NMR (151 MHz, DMSO-*d*$_6$)** δ 192.86, 161.91, 160.11, 152.96, 145.23, 138.43, 137.04, 135.58, 133.93, 133.82, 130.52, 129.48, 128.96, 128.57, 127.69, 120.72, 105.79. **HRMS (*m/z*)** calcd.: for $C_{21}H_{14}N_2O_2$ [M+H]$^+$, 327.1128; found: 327.1129.

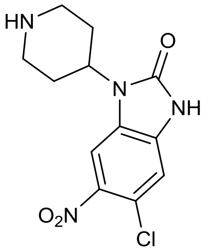

11

Synthesis of 5-chloro-6-nitro-1-(piperidin-4-yl)-1,3-dihydro-2H-benzo[d]imidazol-2-one (**11**)**.** 5-chloro-1-(piperidin-4-yl)-1,3-dihydro-2*H*benzo[*d*]imidazol-2-one (**10**, 2.5 g, 9.9 mmol) was used following Common Procedure A and yielded the title compound as pale yellow solid (2.80 g, 9.4 mmol, 95 %). **^{1}H NMR (400 MHz, DMSO-*d*$_6$)** δ 7.99 (s, 1H), 7.19 (s, 1H), 4.33 (dd, *J* = 16.4, 8.2 Hz, 1H), 3.14 (d, *J* = 12.5 Hz, 2H), 2.68 (t, *J* = 11.9 Hz, 2H), 2.24 (tt, *J* = 12.3, 6.2 Hz, 2H), 1.66 (d, *J* = 11.0 Hz, 2H). **^{13}C NMR (100 MHz,**

DMSO-*d*$_6$) δ 156.9, 140.2, 137.2, 129.5, 119.6, 111.3, 106.2, 51.0, 46.0, 29.7. **HRMS (*m/z*)** calcd.: for $C_{12}H_{14}ClN_4O_3$ [M+H]$^+$, 297.0748; found: 297.0756.

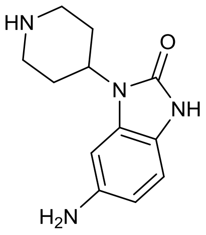

12

Synthesis of 6-amino-1-(piperidin-4-yl)-1,3-dihydro-2H-benzo[d]imidazol-2-one (**12**). 5-chloro-1-(piperidin-4-yl)-1,3-dihydro-2*H*benzo[*d*]imidazol-2-one (**4**) (2 g, 6.7 mmol), 5 % Pd/C moistened with water (0.2 g) and ammonium formate (10 eq., 4.0 g, 67 mmol) were dissolved in methanol (60 mL) and allowed to stir overnight at 80 °C. The reaction mixture was filtered over Celite, evaporated and the crude product was used without further purification. **^{1}H NMR (500 MHz, MeOD-*d*$_4$)** δ 6.86 (s, 1H), 6.84 (d, *J* = 2.2 Hz, 1H), 6.53 (dd, *J* = 8.3, 2.0 Hz, 1H), 4.43 (tt, *J* = 12.3, 4.1 Hz, 1H), 3.45 – 3.38 (m, 2H), 3.00 (td, *J* = 12.9, 2.5 Hz, 2H), 2.59 (qd, *J* = 13.1, 4.2 Hz, 2H), 1.90 (dd, *J* = 12.5, 1.8 Hz, 2H). **^{13}C NMR (125 MHz, MeOD-*d*$_4$)** δ 155.4, 142.4, 130.0, 121.1, 110.0, 109.8, 97.7, 49.5, 44.9, 27.7. **HRMS (m/z)** calcd.: for $C_{12}H_{17}N_4O$ [M+H]$^+$, 233.1396; found: 233.1396.

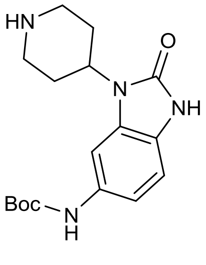

13

Synthesis of tert-butyl(2-oxo-3-(piperidin-4-yl)-2,3-dihydro-1H-benzo[d]imidazol-5-yl)-carbamate (**13**). 6-amino-1-(piperidin-4-yl)-1,3-dihydro-2*H*-benzo[*d*]imidazol-2-one (**12**, 1.5 g, 6.46 mmol) was used following Common Procedure B and yielded the title compound as a white solid (1.1 g, 3.3 mmol, 51 % over 2 steps). **^{1}H NMR (500 MHz, MeOD-*d*$_4$)** δ 7.52 (s, 1H), 7.02 (dd, *J* = 8.4, 1.8 Hz, 1H), 6.97 (d, *J* =

8.4 Hz, 1H), 4.39 (tt, *J* = 12.4, 4.1 Hz, 1H), 3.26 (d, *J* = 12.5 Hz, 2H), 2.81 (td, *J* = 12.3, 1.7 Hz, 2H), 2.41 (qd, *J* = 12.7, 4.1 Hz, 2H), 1.82 (dd, *J* = 12.1, 1.8 Hz, 2H), 1.55 (s, 9H). **^{13}C NMR (125 MHz, MeOD-d_4)** δ 155.6, 154.7, 133.4, 129.6, 124.3, 109.4, 79.8, 50.8, 45.5, 29.3, 27.4. **HRMS (*m/z*)** calcd.: for $C_{17}H_{25}N_4O_3$ [M+H]$^+$, 333.1921; found: 333.1923.

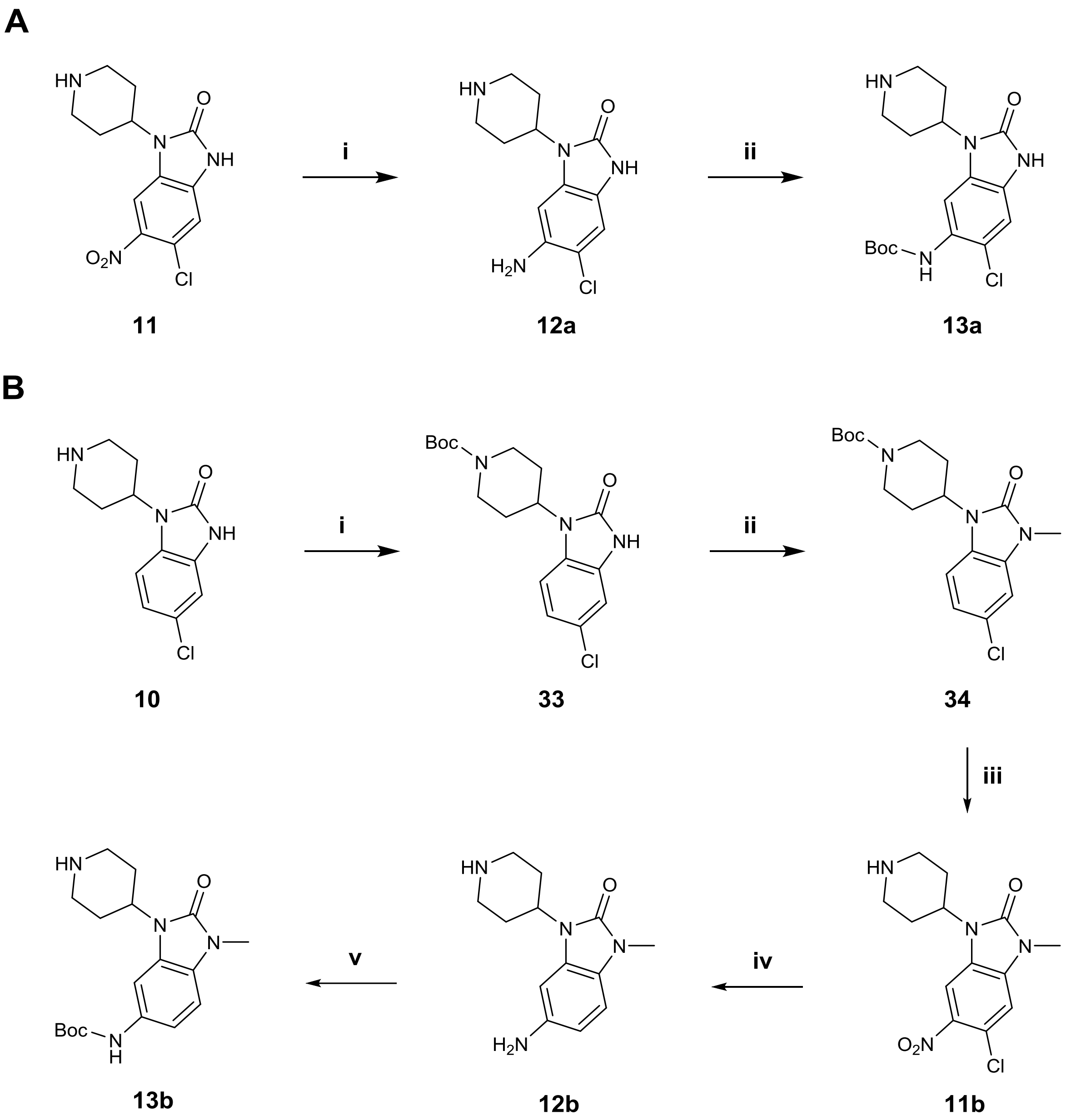

Fig. S1 Synthetic scheme for synthesis of benzo[*d*]imidazolone-based covalent-allosteric inhibitors. **A** Synthesis of chlorinated derivative. i Fe, NH_4Cl, $MeOH/H_2O$, reflux, 2h. ii Boc_2O, 10 % $AcOH/H_2O$, 1,4-dioxane, rt, ovn. **B** Synthesis of methylated derivative. i Boc_2O, DIPEA, DCM, rt, 2 h. ii MeI, NaH, DMF, 0°C - rt, 4 h. iii HNO_3, *o*-xylene, 60 °C, 2 h. iv Pd/C, NH_4HCOO, MeOH, 80 °C, ovn. v Boc_2O, 10 % $AcOH/H_2O$, 1,4-dioxane, rt, ovn.

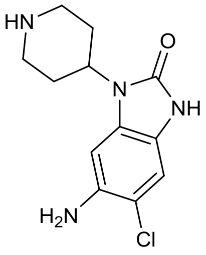

12a

Synthesis of 6-amino-5-chloro-1-(piperidin-4-yl)-1,3-dihydro-2H-benzo[d]imidazol-2-one (**12a**)**.** 5-chloro-6-nitro-1-(piperidin-4-yl)-1,3-dihydro-2*H*-benzo[*d*]imidazol-2-one (**11**, 1.5 g, 5.06 mmol) was used following Common Procedure D. The crude product was used without further purification. **^{1}H NMR (500 MHz, MeOD-*d*$_4$)** δ 6.96 (s, 1H), 6.92 (s, 1H), 4.39 (tt, *J* = 12.4, 4.2 Hz, 1H), 3.36 (dd, *J* = 14.4, 8.1 Hz, 2H), 2.95 (td, *J* = 12.8, 2.3 Hz, 2H), 2.51 (qd, *J* = 13.0, 4.2 Hz, 2H), 1.87 (dd, *J* = 12.4, 1.7 Hz, 2H). **^{13}C NMR (126 MHz, MeOD-*d*$_4$)** δ 155.35, 138.86, 129.39, 121.19, 112.69, 109.79, 97.67, 49.73, 44.90, 27.68. **HRMS (*m/z*)** calcd. for $C_{12}H_{16}N_4OCl$ [M+H]$^+$, 267.1007; found, 267.1009.

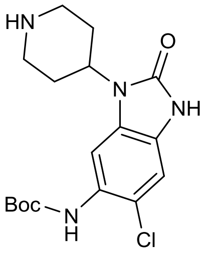

13a

Synthesis of tert-butyl (6-chloro-2-oxo-3-(piperidin-4-yl)-2,3-dihydro-1H-benzo[d]-imidazol-5-yl)carbamate (**13a**)**.** 6-amino-5-chloro-1-(piperidin-4-yl)-1,3-dihydro-2*H*-benzo[*d*]imidazol-2-one (**12a**, 899 mg, 3.37 mmol) was used following Common Procedure A and yielded the title product as a pale yellow solid (415 mg, 1.1 mmol 34 % over 2 steps). **^{1}H NMR (600 MHz, DMSO-*d*$_6$)** δ 8.54 (s, 1H), 7.32 (s, 1H), 7.01 (s, 1H), 4.22 – 4.15 (m, 1H), 3.04 (d, *J* = 11.7 Hz, 1H), 2.56 (t, *J* = 11.8 Hz, 1H), 2.13 (qt, *J* = 12.1, 5.8 Hz, 1H), 1.57 (t, *J* = 11.0 Hz, 1H), 1.44 (s, 9H). **^{13}C NMR (151 MHz, DMSO-*d*$_6$)** δ 153.86, 129.58, 128.23, 128.10, 127.87, 126.89, 121.43, 108.85, 78.88, 50.57, 45.69, 29.98, 28.14. **LCMS (*m/z*)** calcd.: for $C_{17}H_{23}ClN_4O_3$ [M+H]$^+$, 367.15; found: 367.08.

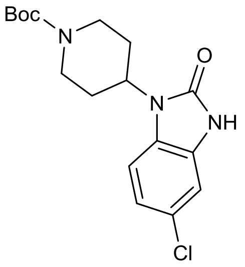

33

Synthesis of tert-butyl 4-(5-chloro-2-oxo-2,3-dihydro-1H-benzo[d]imidazol-1-yl)-piperidine-1-carboxylate (**33**). 5-chloro-1-(piperidin-4-yl)-1,3-dihydro-2*H*-benzo[*d*]-imidazol-2-one (10, 2.0 g, 7.95 mmol) was suspended in DCM. DIPEA (4 eq., 4.1 g, 31.78 mmol) and Boc_2O (2 eq., 3.5 g, 15.89 mmol) were added and the reaction mixture was allowed to stir for 2 h at rt. The mixture was washed with sat. NH_4Cl solution and extracted with dichloromethane. The combined organic fractions were evaporated and silica column chromatography (25 % EtOAc/PE) yielded the pure product as a white solid (2.32 g,13.2 mmol, 83 %). **^{1}H NMR (500 MHz, DMSO-*d*$_6$)** δ 11.06 (s, 1H), 7.22 (d, *J* = 8.4 Hz, 1H), 7.05 – 6.96 (m, 2H), 4.31 (tt, *J* = 12.1, 3.7 Hz, 1H), 4.08 (s, 2H), 2.87 (s, 2H), 2.15 (qd, *J* = 12.5, 4.3 Hz, 2H), 1.67 (d, *J* = 10.8 Hz, 2H), 1.42 (s, 9H). **^{13}C NMR (126 MHz, DMSO-*d*$_6$)** δ 154.00, 153.83, 129.69, 128.42, 125.04, 120.24, 109.86, 108.86, 79.01, 50.31, 43.57, 42.69, 28.68, 28.29. **LCMS (*m/z*)** calcd.: for $C_{17}H_{22}ClN_3O_3$ [M+H]$^+$, 352.13; not ionisable.

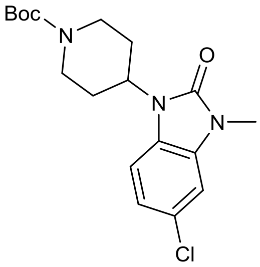

34

Synthesis of tert-butyl 4-(5-chloro-3-methyl-2-oxo-2,3-dihydro-1H-benzo[d]imidazol-1-yl)piperidine-1-carboxylate (**34**). A solution of *tert*-butyl 4-(5-chloro-2-oxo-2,3-dihydro-1*H*-benzo[*d*]imidazol-1-yl)-piperidine-1-carboxylate (**33**, 1.0 g, 2.84 mmol) in 5 mL DMF was cooled to 0 °C by means of an ice bath. After addition of NaH (2 eq., 136.4 mg, 5.68 mmol) the suspension was allowed to stir for 30 min. Then, methyl iodide (2 eq., 806.8 mg, 5.68 mmol) was added dropwise, the ice bath was removed and

the reaction mixture was allowed to stir for 2 h at rt. The mixture was quenched with sat. NH_4Cl solution and extracted with dichloromethane. The combined organic fractions were evaporated and silica column chromatography (25 % EtOAc/PE) yielded the pure product as a white solid (882 mg, 2.4 mmol, 85 %). ^{1}H NMR (500 MHz, DMSO-d_6) δ 7.29 (dd, *J* = 14.5, 5.2 Hz, 1H), 7.06 (dd, *J* = 8.4, 2.0 Hz, 2H), 4.37 (tt, *J* = 12.1, 3.8 Hz, 1H), 4.08 (s, 3H), 3.35 (s, 2H), 2.86 (d, *J* = 26.5 Hz, 2H), 2.16 (qd, *J* = 12.5, 4.3 Hz, 2H), 1.68 (d, *J* = 10.6 Hz, 2H), 1.42 (s, 9H). **^{13}C NMR (126 MHz, DMSO-*d_6*)** δ 153.86, 153.05, 130.93, 126.94, 125.20, 120.35, 109.65, 108.19, 78.87, 50.66, 28.13, 27.04. **LCMS (*m/z*)** calcd.: for $C_{18}H_{24}ClN_3O_3$ $[M+H]^+$, 366.15; not ionisable.

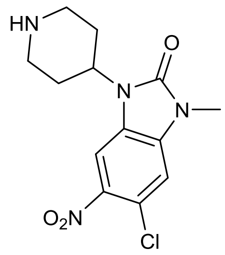

11b

Synthesis of 5-chloro-3-methyl-6-nitro-1-(piperidin-4-yl)-1,3-dihydro-2H-benzo[d]-imidazol-2-one (**11b**). *tert*-butyl 4-(5-chloro-3-methyl-2-oxo-2,3-dihydro-1*H*-benzo[*d*]-imidazol-1-yl)piperidine-1-carboxylate (**34**, 1.0 g, 2.7 mmol) was used following Common Procedure A and yielded the title product as a pale yellow solid (768.0 mg, 2.4 mmol, 90 %). **^{1}H NMR (500 MHz, DMSO-*d_6*)** δ 8.11 (s, 1H), 7.65 (s, 1H), 4.63 (tt, *J* = 12.2, 3.9 Hz, 1H), 3.46 (d, *J* = 12.3 Hz, 2H), 3.37 (s, 3H), 3.08 (q, *J* = 12.3 Hz, 2H), 1.92 (d, *J* = 12.5 Hz, 2H). **^{13}C NMR (126 MHz, DMSO-*d_6*)** δ 153.72, 141.13, 134.58, 127.20, 120.01, 110.64, 106.57, 48.64, 43.48, 28.01, 25.74. **LCMS (*m/z*)** calcd.: for $C_{13}H_{15}ClN_4O_3$ $[M+H]^+$, 311.08; found, 311.35.

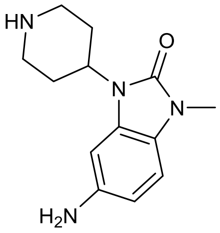

12b

Synthesis of 5-amino-1-methyl-3-(piperidin-4-yl)-1,3-dihydro-2H-benzo[d]imidazol-2-one (**12b**). 5-chloro-3-methyl-6-nitro-1-(piperidin-4-yl)-1,3-dihydro-2*H*-benzo[*d*]-imidazol-2-one (**11b**, 747 mg, 2.4 mmol), 5 % Pd/C moistened with water (0.1 g) and ammonium formate (10 eq., 1.5 g, 24.0 mmol) were dissolved in methanol (40 mL) and allowed to stir overnight at 80 °C. The reaction mixture was filtered over Celite, evaporated and the crude product was used without further purification. **LCMS (*m/z*)** calcd.: for $C_{13}H_{18}N_4O$ $[M+H]^+$, 247.15; found, 247.29.

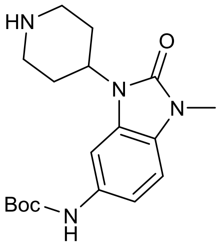

13b

Synthesis of tert-butyl (1-methyl-2-oxo-3-(piperidin-4-yl)-2,3-dihydro-1H-benzo[d]-imidazol-5-yl)carbamate (**13b**). 5-amino-1-methyl-3-(piperidin-4-yl)-1,3-dihydro-2*H*-benzo[*d*]imidazol-2-one (**12b**, 591 mg, 2.4 mmol) was used following Common Procedure B and yielded the title product as a white solid (363 mg, 1.1 mmol, 44 % over 2 steps). **^{1}H NMR (500 MHz, DMSO-*d*$_6$)** δ 9.23 (s, 1H), 7.57 (s, 1H), 7.04 (s, 1H), 7.02 – 6.97 (m, *J* = 8.4 Hz, 1H), 4.20 – 4.11 (m, 1H), 3.38 (b, 1H), 3.26 (s, 3H), 3.06 (d, *J* = 11.9 Hz, 2H), 2.57 (t, *J* = 11.5 Hz, 2H), 2.14 (qd, *J* = 12.2, 3.8 Hz, 2H), 1.59 (d, *J* = 9.5 Hz, 2H), 1.47 (s, 9H). **^{13}C NMR (126 MHz, DMSO-*d*$_6$)** δ 158.41, 158.18, 138.67, 133.02, 130.20, 116.34, 112.78, 105.29, 83.94, 56.36, 51.05, 35.29, 33.41, 32.02. **LCMS (*m/z*)** calcd.: for $C_{18}H_{26}N_4O_3$ $[M+H]^+$, 347.20; found, 347.23.

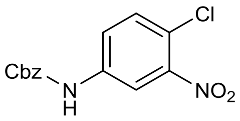

15

Synthesis of benzyl (4-chloro-3-nitrophenyl)carbamate (**15**). 4-chloro-3-nitroaniline (**14**, 2 g, 11.5 mmol) and 1 g $NaHCO_3$ (1.1 eq., 12.7 mmol) were dissolved in 20 mL THF. 1.8 mL benzyl chloroformate (1.1 eq., 12.7 mmol) were added dropwise and the reaction mixture was stirred for 5 h at room temperature. Subsequently, the reaction mixture was extracted with ethyl acetate and washed with water and sat. $NaHCO_3$ solution. The organic phases were dried over Na_2SO_4 and the solvent were removed *in vacuo*. 2.5 g of the orange product (8.0 mmol, 70 %) were received by silica gel column chromatography (20 % EtOAc/PE). **^{1}H-NMR (500 MHz, DMSO-*d*$_6$)** δ 10.40 (s, 1H), 8.23 (s, 1H), 7.71-7.65 (m, 2H), 7.29-7.46 (m, 6H), 5.19 (s, 1H). **^{13}C-NMR (126 MHz, DMSO-*d*$_6$)** δ 153.7, 147.86,139.71, 136.54, 132.56, 129.32, 129.05, 128.76, 128.5, 126.87, 123.48. **LCMS (*m/z*)** calcd.: for $C_{14}H_{11}ClN_2O_4$ $[M+H]^+$, 347.20; not ionisable.

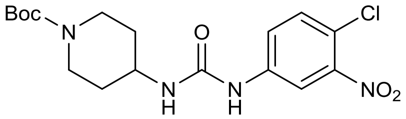

16

Synthesis of tert-butyl 4-(3-(4-chloro-3-nitrophenyl)ureido)piperidine-1-carboxylate (**16**). 1.4 g benzyl (4-chloro-3-nitrophenyl)carbamate (**15**, 4.5 mmol, 1 eq.), 1.1 g *tert*-butyl 4-aminopiperidine-1-carboxylate (5.5 mmol, 1.2 eq.) and 943 mg K_2CO_3 (6.8 mmol, 1.5 eq.) were dissolved in 15 mL DMF and stirred for 90 min at 130 °C in an autoclave vessel. Subsequently, the reaction mixture was extracted with ethyl acetate and washed with water and sat. $NaHCO_3$ solution. 737 mg of the brownish product (1.8 mmol, 40 %) were received by silica gel column chromatography (1 % MeOH/DCM). **^{1}H-NMR (400 MHz, DMSO-*d*$_6$)** δ 8.95 (s, 1H), 8.25 (d, *J* = 2.4 Hz, 1H), 7.6 – 7.56 (m, 1H), 7.56 – 7.52 (m, 1H), 6.44 (s, 1H), 3.82 (d, *J* = 13.2, 2H), 3.69-3.59 (m, 1H), 3.32 (s, 2H), 1.78 (dd, *J* = 12.7 Hz, 3.3 Hz, 2H), 1.39 (s, 9H), 1.28 (dd, *J* = 14.8 Hz,12.3 Hz, 2H). **^{13}C-NMR (101 MHz, DMSO-*d*$_6$)** δ 154.40, 147.93, 141.04,

132.13, 122.95, 116.25, 114.07, 79.12, 55.38, 46.83, 32.16, 28.55. **HRMS (*m/z*)** calcd.: for $C_{17}H_{24}N_4O_5Cl$ $[M+H]^+$, 399.1428; found: 399.1429.

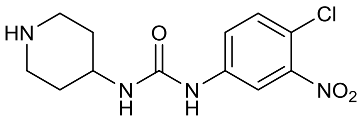

17

Synthesis of 1-(4-chloro-3-nitrophenyl)-3-(piperidin-4-yl)urea (**17**)**.** 1.2 g *tert*-butyl 4-(3-(4-chloro-3-nitrophenyl)ureido)piperidine-1-carboxylate (**16**, 2.9 mmol) were dissolved in 15 mL HCl/1,4-dioxane (4 N) and stirred for 2 h at room temperature. The reaction mixture was extracted between a sat. NH_4Cl solution and ethyl acetate and subsequently extracted between a sat. $NaHCO_3$ solution and ethyl acetate. The combined organic phases were dried over Na_2SO_4 and the solvent was evaporated *in vacuo*. 595 mg (2.0 mmol, 68 %) of the brownish product were received. **^{1}H-NMR (500 MHz, DMSO-*d*$_6$)** δ 9.93 (s, 1H), 8.28 (s, 1H), 7.56 (s, 1H), 7.46 (s, 1H), 3.78-3.37 (m, 2H), 3.23-3.15 (m, 2H), 2.91 (dd, *J* = 17.1, 6.6 Hz, 2H), 1.96- 1.87 (m, 2H), 1.85 (s, 1H), 1.61-1.51 (m, 2H). ^{13}C-NMR (126 MHz, DMSO-*d*$_6$): δ 154.84, 147.9,141.37, 132.1, 122.74, 115.94, 113.75, 44.58, 42.6, 29.65. **HRMS (m/z)** calcd.: for $C_{12}H_{16}N_4O_3Cl$ $[M+H]^+$, 299.0909; found, 299.0905.

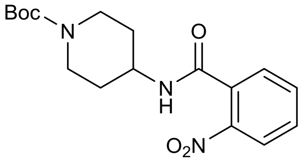

20a

Synthesis of tert-butyl 4-(2-nitrobenzamido)piperidine-1-carboxylate (**20a**)**.** 2-nitrobenzoic acid (3.0 g, 18.0 mmol, 1 eq.) was used following Common Procedure C and yielded the title product as a pale yellow solid (3.7 g, 10.5 mmol, 58 %). **^{1}H NMR (500 MHz, DMSO-*d*$_6$)** δ 8.60 (d, *J* = 7.8 Hz, 1H), 8.05 (dd, *J* = 8.1, 1.0 Hz, 1H), 7.79 (td, *J* = 7.5, 1.2 Hz, 1H), 7.71 – 7.66 (m, 1H), 7.58 (dd, *J* = 7.6, 1.3 Hz, 1H), 3.94 – 3.81 (m, 3H), 2.90 (b, 2H), 1.81 (dd, *J* = 12.8, 3.3 Hz, 2H), 1.40 (s, 9H), 1.37 – 1.28 (m, 2H). **^{13}C NMR**

(126 MHz, DMSO-d_6) δ 164.88, 153.95, 146.79, 133.78, 132.84, 130.60, 129.15, 124.10, 78.69, 46.26, 30.84, 28.10. **LCMS (*m/z*)** calcd.: for $C_{17}H_{23}N_3O_5$ $[M+H]^+$, 350.16; not ionisable.

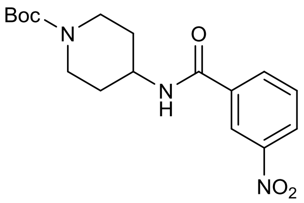

20b

Synthesis of tert-butyl 4-(3-nitrobenzamido)piperidine-1-carboxylate (**20b**). 3-nitro-benzoic acid (331 mg, 2.0 mmol, 1 eq.) was used following Common Procedure C and yielded the title product as a pale yellow solid (528 mg, 1.5 mmol, 76 %). **^{1}H NMR (500 MHz, DMSO-d_6)** δ 8.51 (t, *J* = 1.9 Hz, 1H), 8.27 (ddd, *J* = 8.2, 2.2, 1.0 Hz, 1H), 8.12 – 8.08 (m, 1H), 7.56 (t, *J* = 8.0 Hz, 1H), 6.43 (d, *J* = 7.7 Hz, 1H), 4.24 – 3.90 (m, 3H), 2.82 (dd, *J* = 20.0, 7.7 Hz, 2H), 2.05 – 1.87 (m, 2H), 1.46-1.33 (m, 11H).**^{13}C NMR (126 MHz, DMSO-d_6)** δ 164.36, 154.64, 148.16, 135.92, 133.13, 129.92, 125.83, 121.28, 79.55, 47.89, 42.72, 31.76, 28.29. **LCMS (*m/z*)** calcd.: for $C_{17}H_{23}N_3O_5$ $[M+H]^+$, 350.16; not ionisable.

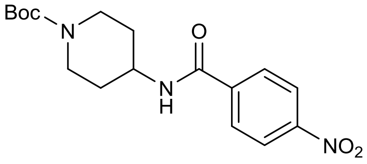

20c

Synthesis of tert-butyl 4-(4-nitrobenzamido)piperidine-1-carboxylate (**20c**). 4-nitro-benzoic acid (100 mg, 2.0 mmol, 1 eq.) was used following Common Procedure C and yielded the title product as a pale yellow solid (140 mg, 0.4 mmol, 67 %). **^{1}H NMR (600 MHz, DMSO-d_6)** δ 8.63 (d, *J* = 7.8 Hz, 1H), 8.33 – 8.28 (m, 2H), 8.09 – 8.04 (m, 2H), 4.03 – 3.88 (m, 3H), 2.95 – 2.76 (m, 2H), 1.82 – 1.77 (m, 2H), 1.44 – 1.37 (m, 11H).**^{13}C NMR (151 MHz, DMSO-d_6)** δ 163.92, 153.94, 148.97, 140.22, 128.83, 123.50, 78.70, 46.85, 43.01, 31.24, 28.10. **LCMS (*m/z*)** calcd.: for $C_{17}H_{23}N_3O_5$ $[M+H]^+$, 350.16; not ionisable.

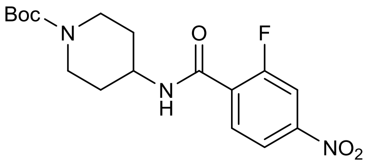

20d

Synthesis of tert-butyl 4-(2-fluoro-4-nitrobenzamido)piperidine-1-carboxylate (**20d**). 2-Fluoro-4-nitrobenzoic acid (1.5 g, 8.1 mmol) was used following Common Procedure C and yielded the title product as a pale yellow solid (1.8 g, 4.9 mmol, 60 %). **^{1}H NMR (500 MHz, DMSO-*d*$_6$)** δ 8.65 (d, *J* = 7.7 Hz, 1H), 8.21 (dd, *J* = 9.6, 2.1 Hz, 1H), 8.13 (dd, *J* = 8.4, 2.1 Hz, 1H), 7.80 (dd, *J* = 8.4, 7.0 Hz, 1H), 4.00 – 3.92 (m, 1H), 3.88 (d, *J* = 10.9 Hz, 2H), 2.89 (b, 2H), 1.81 (dd, *J* = 12.7, 3.1 Hz, 2H), 1.44 – 1.30 (m, 11H). **^{13}C NMR (126 MHz, DMSO-*d*$_6$)** δ 161.74, 159.37, 157.36, 153.94, 148.99 (d, *J* = 8.8 Hz), 131.02 (d, *J* = 3.6 Hz), 130.85, 119.64, 112.15, 111.93, 78.73, 46.56, 43.06 – 42.36, 42.36 – 41.69, 31.06, 28.10. **LCMS (*m/z*)** calcd.: for $C_{17}H_{22}FN_3O_5$ $[M+H]^+$, 368.15; not ionisable.

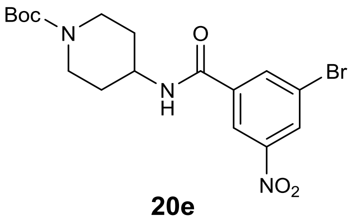

20e

Synthesis of tert-butyl 4-(3-bromo-5-nitrobenzamido)piperidine-1-carboxylate (**20e**). 3-bromo-5-nitrobenzoic acid (100 mg, 0.4 mmol) was used following Common Procedure C and yielded the title product as a yellow solid (130 mg, 0.3 mmol, 77 %). **^{1}H NMR (500 MHz, DMSO-*d*$_6$)** δ 8.74 (d, *J* = 7.7 Hz, 1H), 8.65 – 8.64 (m, 1H), 8.54 (t, *J* = 1.9 Hz, 1H), 8.47 (t, *J* = 1.4 Hz, 1H), 3.99 (tdd, *J* = 26.6, 13.7, 9.5 Hz, 3H), 2.86 (d, *J* = 38.5 Hz, 2H), 1.81 (dd, *J* = 12.5, 2.6 Hz, 2H), 1.41 (s, 11H). **^{13}C NMR (126 MHz, DMSO-*d*$_6$)** δ 161.93, 153.88, 148.59, 137.27, 136.06, 128.49, 122.18, 121.38, 78.71, 47.05, 42.95, 42.58 – 41.81, 31.20, 28.10. **LCMS (*m/z*)** calcd.: for $C_{17}H_{22}BrN_3O_5$ $[M+H]^+$, 428.07; not ionisable.

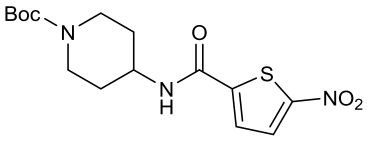

20f

Synthesis of tert-butyl 4-(5-nitrothiophene-2-carboxamido)piperidine-1-carboxylate (**20f**)**.** 5-nitrothiophene-2-carboxylic acid (1.5 g, 8.7 mmol) was used following Common Procedure C and yielded the title product as a white solid (2.0 g, 5.7 mmol, 66 %). **^{1}H NMR (500 MHz, CDCl$_3$)** δ 7.78 (d, *J* = 4.3 Hz, 1H), 7.41 (d, *J* = 4.3 Hz, 1H), 6.49 (d, *J* = 7.8 Hz, 1H), 4.12 – 3.99 (m, 3H), 2.85 – 2.76 (m, 2H), 1.94 (d, *J* = 11.1 Hz, 2H), 1.38 (s, 11H). **^{13}C NMR (126 MHz, CDCl$_3$)** δ 159.49, 154.81, 154.28, 145.18, 128.30, 126.21, 80.15, 47.90, 42.85, 32.07, 28.54. **LCMS (*m/z*)** calcd.: for $C_{15}H_{21}N_3O_5S$ [M+H]$^+$, 356.12; not ionisable.

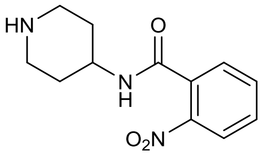

21a

Synthesis of 2-nitro-N-(piperidin-4-yl)benzamide (**21a**)**.** *tert*-butyl 4-(2-nitrobenz-amido)piperidine-1-carboxylate (2.0 g, 5.7 mmol) was used following Common Procedure F and yielded the title product as a pale yellow solid (1.4 g, 5.6 mmol, 98 %). **^{1}H NMR (500 MHz, DMSO-*d*$_6$)** δ 8.67 – 8.56 (m, 1H), 8.05 (dd, *J* = 8.1 Hz, 1.0 Hz, 1H), 7.78 (td, *J* = 7.5 Hz, 1.0 Hz, 1H), 7.68 (td, *J* = 8.1 Hz, 1.3 Hz, 1H), 7.58 (dd, *J* = 7.6 Hz, 1.3 Hz, 1H), 4.36 (s, 1H), 3.87 – 3.77 (m, 1H), 3.02 (d, *J* = 12.3 Hz, 1H), 2.64 (m, 2H), 1.82 (d, *J* = 12.3 Hz, 2H), 1.43 (dd, *J* = 20.5 Hz, 10.2 Hz, 2H). **^{13}C NMR (126 MHz, DMSO-*d*$_6$)** δ 164.84, 146.83, 133.74, 132.96, 130.54, 129.19, 124.06, 66.39, 46.34, 44.02, 31.10. **LCMS (*m/z*)** calcd.: for $C_{12}H_{15}N_3O_3$ [M+H]$^+$, 250.11; found, 250.16.

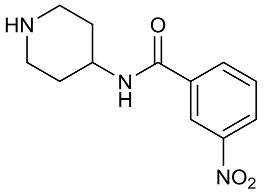

21b

Synthesis of 3-nitro-N-(piperidin-4-yl)benzamide (**21b**). *tert*-butyl 4-(3-nitrobenz-amido)piperidine-1-carboxylate (2.0 g, 5.7 mmol) was used following Common Procedure F and yielded the title product as a pale yellow solid (1.3 g, 5.4 mmol, 94 %). **^{1}H NMR (500 MHz, DMSO-*d*$_6$)** δ 8.72 – 8.64 (m, 2H), 8.40 – 8.35 (m, 1H), 8.30 (d, *J* = 7.8 Hz, 1H), 7.77 (t, *J* = 8.0 Hz, 1H), 3.91 – 3.81 (m, 1H), 3.33 (s, 1H), 2.97 (d, *J* = 12.3 Hz, 2H), 2.59 – 2.51 (m, 2H), 1.76 (d, *J* = 11.6 Hz, 2H), 1.43 (qd, *J* = 12.0, 3.8 Hz, 2H). **^{13}C NMR (126 MHz, DMSO-*d*$_6$)** δ 163.21, 147.73, 136.11, 133.90, 130.04, 125.77, 122.03, 47.75, 45.21, 32.70. **LCMS (*m/z*)** calcd.: for $C_{12}H_{15}N_3O_3$ [M+H]$^+$, 250.11; found, 250.17.

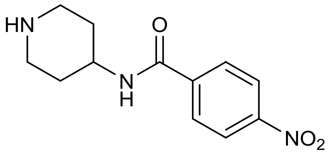

21c

Synthesis of 3-nitro-N-(piperidin-4-yl)benzamide (**21c**). *tert*-butyl 4-(3-nitrobenz-amido)piperidine-1-carboxylate (2.8 g, 8.1 mmol) was used following Common Procedure F and yielded the title product as a pale yellow solid (1.8 g, 7.2 mmol, 89 %). **^{1}H NMR (500 MHz, DMSO-*d*$_6$)** δ 8.62 (d, *J* = 7.7 Hz, 1H), 8.34 – 8.27 (m, 2H), 8.10 – 8.04 (m, 2H), 3.87 – 3.76 (m, 1H), 2.97 (t, *J* = 13.0 Hz, 2H), 2.20 (s, 1H), 1.74 (dd, *J* = 11.8, 2.2 Hz, 2H), 1.41 (tt, *J* = 12.0, 6.0 Hz, 2H). **^{13}C NMR (126 MHz, DMSO-*d*$_6$)** δ 163.74, 148.89, 140.47, 128.85, 123.44, 47.84, 45.29, 32.82. **LCMS (*m/z*)** calcd.: for $C_{12}H_{15}N_3O_3$ [M+H]$^+$, 250.11; found, 250.16.

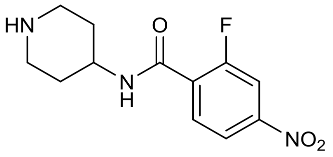

21d

Synthesis of 2-fluoro-4-nitro-N-(piperidin-4-yl)benzamide (**21d**). *tert*-butyl 4-(2-fluoro-4-nitrobenzamido)piperidine-1-carboxylate (20d, 1.0 g, 2.7 mmol) was used following Common Procedure F and yielded the title product as a pale yellow solid (0.6 g, 2.3 mmol, 86 %). **^{1}H NMR (700 MHz, DMSO-*d_6*)** δ 8.58 (d, *J* = 7.7 Hz, 1H), 8.19 (dd, *J* = 9.6, 2.1 Hz, 1H), 8.15 – 8.09 (m, 1H), 7.78 (dd, *J* = 8.2, 7.1 Hz, 1H), 3.85 – 3.74 (m, 1H), 2.93 (dd, *J* = 9.3, 3.2 Hz, 2H), 2.55 – 2.50 (m, 2H), 2.04 (b, 1H), 1.79 – 1.71 (m, 2H), 1.35 (qd, *J* = 11.8, 3.9 Hz, 2H). **^{13}C NMR (176 MHz, DMSO-*d_6*)** δ 161.51, 159.15, 157.48, 148.85 (d, *J* = 8.9 Hz), 131.22 (d, *J* = 16.6 Hz), 130.93 (d, *J* = 3.8 Hz), 119.55 (d, *J* = 3.5 Hz), 111.93 (d, *J* = 27.7 Hz), 47.55, 44.97, 32.71. **LCMS (*m/z*)** calcd.: for $C_{12}H_{14}FN_3O_3$ $[M+H]^+$, 268.10; found, 268.22.

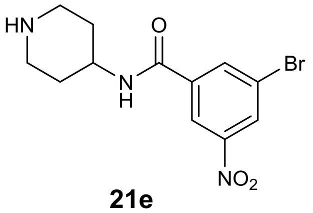

21e

Synthesis of 3-bromo-5-nitro-N-(piperidin-4-yl)benzamide (**21e**). *tert*-butyl 4-(3-bromo-5-nitrobenzamido)piperidine-1-carboxylate (20e, 134 mg, 0.3 mmol) was used following Common Procedure F and yielded the title product as pale yellow solid (101 mg, 0.3 mmol, 99 %). **^{1}H NMR (600 MHz, DMSO-*d_6*)** δ 8.77 (d, *J* = 7.6 Hz, 1H), 8.67 – 8.63 (m, 1H), 8.52 (t, *J* = 1.9 Hz, 1H), 8.49 (t, *J* = 1.5 Hz, 1H), 3.91 – 3.83 (m, 1H), 3.72 (b, 1H), 3.01 (d, *J* = 12.5 Hz, 2H), 2.58 (td, *J* = 12.2, 2.0 Hz, 2H), 1.78 (dd, *J* = 11.9, 2.0 Hz, 2H), 1.47 (qd, *J* = 12.1, 3.9 Hz, 2H). **^{13}C NMR (151 MHz, DMSO-*d_6*)** δ 161.81, 148.56, 137.43, 136.09, 128.34, 122.09, 121.40, 47.57, 44.76, 32.05. **LCMS (*m/z*)** calcd.: for $C_{12}H_{14}BrN_3O_3$ $[M+H]^+$, 328.02; found, 328.11.

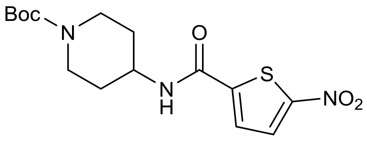

21f

Synthesis of 5-nitro-N-(piperidin-4-yl)thiophene-2-carboxamide (**21f**). *tert*-butyl 4-(5-nitrothiophene-2-carboxamido)piperidine-1-carboxylate (20f, 4.3 g, 12.1 mmol) was used following Common Procedure F and yielded the title product as a white solid (2.0 g, 8.0 mmol, 66 %). **^{1}H NMR (500 MHz, DMSO-*d*$_6$)** δ 8.78 (d, *J* = 7.8 Hz, 1H), 8.14 (d, *J* = 4.4 Hz, 1H), 7.85 (t, *J* = 5.3 Hz, 1H), 3.82 – 3.72 (m, 1H), 2.95 (d, *J* = 12.4 Hz, 2H), 2.47 (dd, *J* = 12.2, 2.0 Hz, 2H), 1.73 (dd, *J* = 11.8, 2.1 Hz, 2H), 1.39 (qd, *J* = 12.1, 4.0 Hz, 2H). **^{13}C NMR (126 MHz, DMSO-*d*$_6$)** δ 158.53, 152.81, 146.96, 130.25, 127.28, 48.03, 45.19, 32.73. **LCMS (*m/z*)** calcd.: for $C_{10}H_{13}N_3O_3S$ [M+H]$^+$, 256.07; found, 256.16.

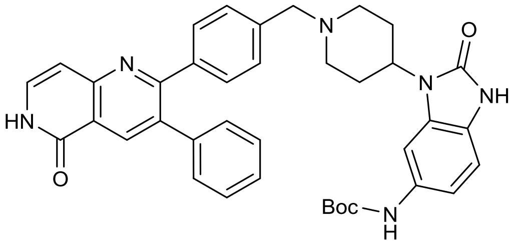

22

Synthesis of tert-butyl (2-oxo-3-(1-(4-(5-oxo-3-phenyl-5,6-dihydro-1,6-naphthyridin-2-yl)-benzyl)-piperidin-4-yl)-2,3-dihydro-1H-benzo[d]imidazol-5-yl)carbamate (**22**). 4-(5-oxo-3-phenyl-5,6-dihydro-1,6-naphthyridin-2-yl)benzaldehyde (**9**, 300 mg, 0.9 mmol) and *ter*t-butyl (2-oxo-3-(piperidin-4-yl)-2,3-dihydro-1*H*benzo[*d*]imidazol-5-yl)carba-mate (**13**, 1.1 eq., 336 mg, 1.0 mmol) were used following Common Procedure E and yielded the title product as a pale yellow solid (318 mg, 0.5 mmol, 54 %). **^{1}H NMR (500 MHz, MeOD-*d*$_4$)** δ 8.64 (s, 1H), 7.43 – 7.38 (m, 3H), 7.35 (d, *J* = 7.4 Hz, 1H), 7.31 (d, *J* = 8.1 Hz, 2H), 7.28 – 7.22 (m, 4H), 7.22 – 7.13 (m, 3H), 6.93 (d, *J* = 8.5 Hz, 1H), 6.89 (d, *J* = 7.4 Hz, 1H), 4.29 (tt, *J* = 16.3, 8.5 Hz, 1H), 3.63 (s, 2H), 3.07 (d, *J* = 11.0 Hz, 2H), 2.46 (dd, *J* = 23.3, 10.9 Hz, 2H), 2.26 (t, *J* = 11.0, 2H), 1.77 (d, *J* = 12.0 Hz, 2H), 1.51 (s, 9H). **^{13}C NMR (125 MHz, MeOD-*d*$_4$)** δ 163.9, 162.7, 155.8, 154.4, 153.5, 139.1, 138.1, 135.5, 132.2, 130.4, 130.3, 129.9, 129.7, 129.66, 129.6, 129.4, 128.6, 127.9,

124.3, 121.1, 109.7, 107.9, 77.7, 62.5, 53.2, 50.6, 28.6. **HRMS (*m/z*)** calcd.: for $C_{38}H_{39}N_6O_4$ [M+H]$^+$, 643.3027; found: 643.3039.

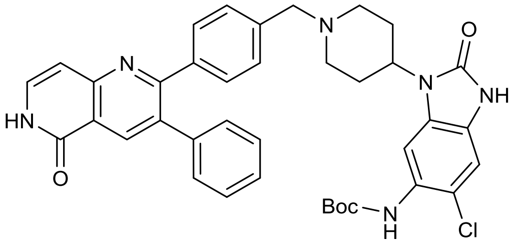

22a

Synthesis of tert-butyl (6-chloro-2-oxo-3-(1-(4-(5-oxo-3-phenyl-5,6-dihydro-1,6-naphthyridin-2-yl)benzyl)piperidin-4-yl)-2,3-dihydro-1H-benzo[d]imidazol-5-yl)carba-mate (**22**). 4-(5-oxo-3-phenyl-5,6-dihydro-1,6-naphthyridin-2-yl)benzaldehyde (**9**, 89 mg, 0.3 mmol) and *tert*-butyl (6-chloro-2-oxo-3-(piperidin-4-yl)-2,3-dihydro-1*H*-benzo[*d*]imidazol-5-yl)carbamate (**13a**, 1.1 eq., 110 mg, 0.3 mmol) were used following Common Procedure E and yielded the title product as a pale yellow solid (99 mg, 0.1 mmol, 54 %). **^{1}H NMR (600 MHz, DMSO-*d*$_6$)** δ 11.58 (s, 1H), 10.97 (s, 1H), 8.52 (s, 1H), 8.38 (s, 1H), 7.49 (d, *J* = 3.2 Hz, 1H), 7.36 – 7.29 (m, 6H), 7.28 – 7.21 (m, 4H), 7.00 (s, 1H), 6.68 (d, *J* = 7.3 Hz, 1H), 4.16 – 4.08 (m, 1H), 3.51 (s, 2H), 2.89 (d, *J* = 11.0 Hz, 2H), 2.30 (td, *J* = 12.0, 8.7 Hz, 2H), 2.09 (t, *J* = 11.2 Hz, 2H), 1.63 (d, *J* = 10.1 Hz, 2H), 1.44 (s, 9H). **^{13}C NMR (151 MHz, DMSO-*d*$_6$)** δ 162.47, 161.64, 154.36, 154.04, 153.39, 139.51, 139.44, 138.60, 137.27, 134.17, 133.94, 130.13, 130.01, 129.84, 128.87, 128.73, 128.64, 128.56, 128.46, 127.91, 127.09, 125.21, 121.19, 120.71, 109.35, 108.06, 106.39, 79.51, 61.79, 52.83, 50.65, 49.07, 28.95, 28.58. **LCMS (*m/z*)** calcd.: for $C_{38}H_{37}ClN_6O_4$ [M+H]$^+$, 677.26; found, 677.28.

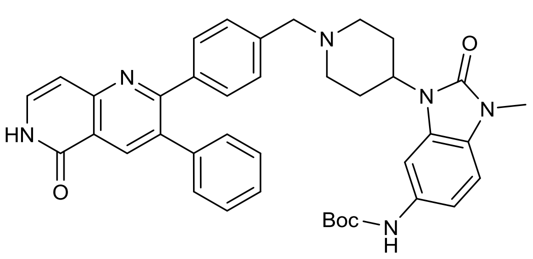

22b

Synthesis of tert-butyl (1-methyl-2-oxo-3-(1-(4-(5-oxo-3-phenyl-5,6-dihydro-1,6-naph-thyridin-2-yl)-benzyl)piperidin-4-yl)-2,3-dihydro-1H-benzo[d]imidazol-5-yl)carba-mate (**22b**). 4-(5-oxo-3-phenyl-5,6-dihydro-1,6-naphthyridin-2-yl)benzaldehyde (**9**, 152 mg, 0.5 mmol) and *tert*-butyl (1-methyl-2-oxo-3-(piperidin-4-yl)-2,3-dihydro-1*H*-benzo[*d*]imidazol-5-yl)carbamate (**13b**, 1.1 eq., 178 mg, 0.5 mmol) were used following Common Procedure E and yielded the title product as a pale yellow solid (223 mg, 0.1 mmol, 73 %). **^{1}H NMR (400 MHz, DMSO-*d*$_6$)** δ 9.21 (s, 1H), 8.38 (s, 1H), 7.54 – 7.46 (m, 2H), 7.37 – 7.30 (m, 6H), 7.29 (d, *J* = 5.3 Hz, 1H), 7.27 – 7.22 (m, 3H), 7.11 (s, 1H), 7.00 (d, *J* = 8.5 Hz, 1H), 6.68 (d, *J* = 7.3 Hz, 1H), 4.21 – 4.08 (m, 1H), 3.54 (s, 2H), 3.26 (s, 3H), 2.92 (d, *J* = 11.0 Hz, 2H), 2.31 (td, *J* = 12.1, 9.4 Hz, 2H), 2.10 (t, *J* = 11.3 Hz, 2H), 1.63 (d, *J* = 9.5 Hz, 2H), 1.47 (s, 9H). **^{13}C NMR (151 MHz, DMSO-*d*$_6$)** δ 161.99, 161.16, 153.22, 152.93, 139.03, 138.85, 138.16, 136.79, 133.70, 133.47, 129.66, 129.36, 128.41, 128.16, 127.76, 127.45, 124.99, 120.24, 107.56, 105.91, 78.75, 61.23, 52.45, 50.56, 28.47, 28.19. **LCMS (*m/z*)** calcd.: for $C_{39}H_{40}N_6O_4$ [M+H]$^+$, 657.31; found, 654.34.

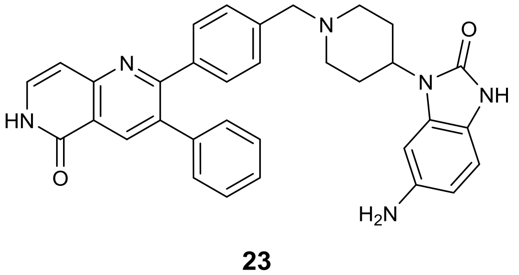

23

Synthesis of 2-(4-((4-(6-amino-2-oxo-2,3-dihydro-1H-benzo[d]imidazol-1-yl)piperidin-1-yl)methyl)-phenyl)-3-phenyl-1,6-naphthyridin-5(6H)-one (**23**). tert-Butyl (2-oxo-3-(1-(4-(5-oxo-3-phenyl-5,6-dihydro-1,6-naphthyridin-2-yl)-benzyl)piperidin-4-yl)-2,3-dihyd-ro-1*H*-benzo[*d*]imidazol-5-yl)carba-mate (**22**, 200 mg, 0.3 mmol) was used following Common Procedure F and yielded the title product as a yellow solid (120 mg, 0.2 mmol, 75 %). **^{1}H NMR (500 MHz, MeOD-*d*$_4$)** δ 8.65 (s, 1H), 7.71 (s, 2H),

7.48 – 7.40 (m, 3H), 7.37 (d, *J* = 8.1 Hz, 2H), 7.32 – 7.27 (m, 3H), 7.26 – 7.19 (m, 2H), 6.89 (d, *J* = 7.4 Hz, 1H), 6.84 (m, 1H), 6.52 (dd, *J* = 8.2, 1.9 Hz, 1H), 4.42 (br, 2H), 4.32 (dd, *J* = 14.5, 10.2 Hz, 1H), 3.74 (s, 2H), 3.15 (d, *J* = 12.0 Hz, 2H), 2.54 (dt, *J* = 21.9, 11.1 Hz, 2H), 2.37 (t, *J* = 11.8 Hz, 2H), 1.81 (d, *J* = 11.5 Hz, 2H). **^{13}C NMR (125 MHz, MeOD-*d*$_4$)** δ 162.8, 161.7, 155.5, 149.8, 145.3, 141.8, 139.3, 139.0, 138.0, 135.5, 132.6, 130.4, 130.3, 129.8, 129.7, 128.6, 127.9, 127.8, 114.2, 112.4, 109.9, 107.4, 66.0, 60.6, 53.1, 28.1. **HRMS (*m/z*)** calcd.: for $C_{33}H_{31}N_6O_2$ [M+H]$^+$, 543.2503; found:543.2495.

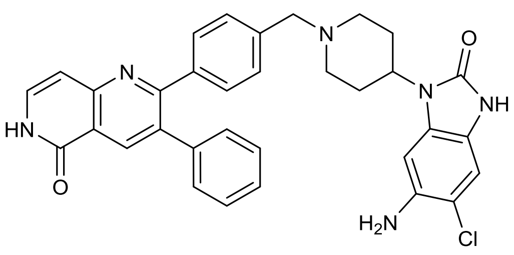

23a

Synthesis of 2-(4-((4-(6-amino-5-chloro-2-oxo-2,3-dihydro-1H-benzo[d]imidazol-1-yl)piperidin-1-yl)-methyl)phenyl)-3-phenyl-1,6-naphthyridin-5(6H)-one (**23a**)**.** *tert*-Butyl (6-chloro-2-oxo-3-(1-(4-(5-oxo-3-phenyl-5,6-dihydro-1,6-naphthyridin-2-yl)benzyl)-piperidin-4-yl)-2,3-dihydro-1*H*-benzo[*d*]imidazol-5-yl)carbamate (**22**, 90 mg, 0.1 mmol) was used following Common Procedure F and yielded the title product as a yellow solid (60 mg, 78 %). **^{1}H NMR (500 MHz, MeOD-*d*$_4$)** δ 8.65 (s, 1H), 7.59 (d, *J* = 1.2 Hz, 1H), 7.40 (d, *J* = 7.8 Hz, 3H), 7.31 (d, *J* = 8.2 Hz, 2H), 7.29 – 7.25 (m, 3H), 7.24 – 7.18 (m, 2H), 6.95 (s, 1H), 6.91 (s, 1H), 6.89 (d, *J* = 7.4 Hz, 1H), 4.33 – 4.22 (m, 1H), 3.61 (s, 2H), 3.06 (d, *J* = 11.7 Hz, 2H), 2.42 (td, *J* = 12.5, 9.1 Hz, 2H), 2.20 (t, *J* = 11.2 Hz, 2H), 1.76 (d, *J* = 10.6 Hz, 2H). **^{13}C NMR (126 MHz, MeOD-*d*$_4$)** δ 163.94, 162.82, 159.25, 153.53, 139.13, 139.00, 138.23, 138.11, 135.59, 132.45, 130.27, 129.90, 129.54, 129.12, 128.63, 127.89, 121.16, 113.37, 110.40, 107.69, 98.55, 53.32, 50.60, 28.60. **HRMS (*m/z*)** calcd. for $C_{33}H_{30}N_6O_3Cl$ [M+H]$^+$, 577.2113; found, 577.2119.

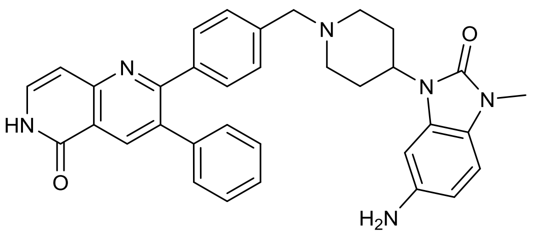

23b

Synthesis of 2-(4-((4-(6-amino-3-methyl-2-oxo-2,3-dihydro-1H-benzo[d]imidazol-1-yl)piperidin-1-yl)methyl)phenyl)-3-phenyl-1,6-naphthyridin-5(6H)-one (**23b**). *tert*-Butyl (1-methyl-2-oxo-3-(1-(4-(5-oxo-3-phenyl-5,6-dihydro-1,6-naphthyridin-2-yl)benzyl)-piperidin-4-yl)2,3-dihydro-1*H*-benzo[*d*]imidazol-5-yl)carbamate (**22b**, 0.22 g, 0.3 mmol) was used following Common Procedure F and yielded the title product as a pale yellow solid (182 mg, 0.3 mmol, 98 %). **^{1}H-NMR (500 MHz, DMSO-*d*$_6$)** δ 8.38 (s, 1H), 7.53 – 7.46 (m, 1H), 7.36 – 7.28 (m, 6H), 7.25 (t, *J* = 7.7 Hz, 4H), 6.78 (d, *J* = 8.2 Hz, 1H), 6.68 (d, *J* = 7.3 Hz, 1H), 6.60 (s, 1H), 6.29 (d, *J* = 8.2 Hz, 1H), 4.87 (b, 2H), 4.09 (t, *J* = 12.2 Hz, 1H), 3.51 (s, 2H), 3.20 (s, 3H), 2.91 (d, *J* = 10.6 Hz, 2H), 2.29 (dd, *J* = 21.5, 11.9 Hz, 2H), 2.06 (t, *J* = 11.3 Hz, 2H), 1.60 (d, *J* = 10.4 Hz, 2H). **^{13}C NMR (126 MHz, DMSO-*d*$_6$)** δ 167.22, 166.39, 158.30, 158.13, 148.87, 144.24, 144.00, 143.39, 141.98, 138.92, 138.77, 134.88, 134.60, 133.83, 133.65, 133.48, 132.69, 126.04, 125.46, 113.34, 111.87, 111.14, 101.06, 66.70, 57.85, 55.59, 33.88, 31.92. **LCMS (*m/z*)** calcd.: for $C_{39}H_{40}N_6O_4$ $[M+H]^+$, 557.26; found, 557.32.

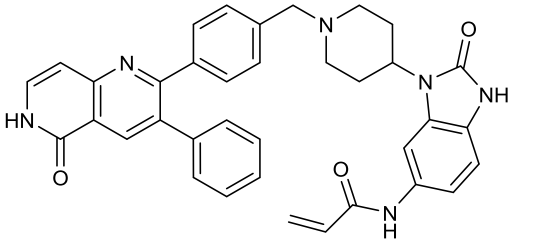

1

Synthesis of N-(2-oxo-3-(1-(4-(5-oxo-3-phenyl-5,6-dihydro-1,6-naphthyridin-2-yl)-benzyl)piperidin-4-yl)-2,3-dihydro-1H-benzo[d]imidazol-5-yl)acrylamide (borussertib, **1**). 2-(4-((4-(6-amino-2-oxo-2,3-dihydro-1*H*-benzo[*d*]imidazol-1-yl)piperidin-1-yl)-methyl)phenyl)-3-phenyl-1,6-naphthyridin-5(6*H*)-one (**23**, 50 mg, 0.1 mmol) was used following Common Procedure G and yielded the title product as an off white solid (45 mg, 0.1 mmol, 82 %). **^{1}H NMR (600 MHz, MeOD-*d*$_4$)** δ 8.60 (s, 1H), 7.77 (s, 1H),

7.48 (d, *J* = 7.4 Hz, 1H), 7.39 (dd, *J* = 26.4, 8.1 Hz, 4H), 7.31 – 7.28 (m, 3H), 7.25 – 7.20 (m, 3H), 7.02 (d, *J* = 8.4 Hz, 1H), 6.87 (d, *J* = 7.4 Hz, 1H), 6.44 (dd, *J* = 16.9, 9.9 Hz, 1H), 6.37 (dd, *J* = 16.9, 1.6 Hz, 1H), 5.78 (dd, *J* = 10.0, 1.6 Hz, 1H), 4.30 (t, *J* = 12.3 Hz, 1H), 3.72 (s, 2H), 3.13 (d, *J* = 10.5 Hz, 2H), 2.56 (dt, *J* = 22.1, 11.0 Hz, 2H), 2.36 (s, 2H), 1.81 (d, *J* = 11.3 Hz, 2H). **^{13}C NMR (151 MHz, MeOD-*d*$_4$)** δ 166.05, 164.74, 163.81, 156.58, 154.52, 140.23, 138.69, 136.55, 133.80, 133.74, 132.48, 131.23, 130.81, 130.50, 129.53, 128.79, 127.60, 126.59, 121.93, 115.44, 110.41, 108.15, 103.93, 62.91, 53.93, 51.91, 29.37. **HRMS (*m/z*)** calcd.: for $C_{36}H_{33}N_6O_3$ [M+H]$^+$, 597.2608; found: 597.2614.

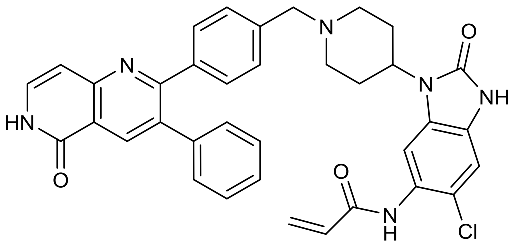

24a

Synthesis of N-(6-chloro-2-oxo-3-(1-(4-(5-oxo-3-phenyl-5,6-dihydro-1,6-naphthyridin-2-yl)-benzyl)piperidin-4-yl)-2,3-dihydro-1H-benzo[d]imidazol-5-yl)acrylamide (**24a**)**.** 2-(4-((4-(6-amino-5-chloro-2-oxo-2,3-dihydro-1*H*-benzo[*d*]imidazol-1-yl)piperidin-1-yl)-methyl)phenyl)-3-phenyl-1,6-naphthyridin-5(6*H*)-one (**23a**, 40 mg, 0.1 mmol) was used following Common Procedure D and yielded the title product as a pale yellow solid (33 mg, 0.05 mmol, 75 %). **^{1}H-NMR (600 MHz, DMSO-*d*$_6$)** δ 11.58 (d, *J* = 4.1 Hz, 1H), 11.02 (s, 1H), 9.68 (s, 1H), 8.38 (s, 1H), 7.55 (s, 1H), 7.49 (t, *J* = 6.4 Hz, 1H), 7.36 – 7.29 (m, 5H), 7.27 – 7.22 (m, 4H), 7.06 (s, 1H), 6.68 (d, *J* = 7.2 Hz, 1H), 6.58 (dd, *J* = 17.0, 10.3 Hz, 1H), 6.27 (d, *J* = 17.0 Hz, 1H), 5.77 (d, *J* = 10.3 Hz, 1H), 4.11 (t, *J* = 12.1 Hz, 1H), 3.51 (s, 2H), 2.90 (d, *J* = 9.6 Hz, 2H), 2.29 (dd, *J* = 21.3, 11.8 Hz, 2H), 2.07 (t, *J* = 11.6 Hz, 2H), 1.64 (d, *J* = 10.2 Hz, 2H). **^{13}C-NMR (151 MHz, DMSO-*d*$_6$)** δ 163.62, 162.00, 161.20, 153.91, 152.92, 139.03, 138.77, 138.15, 136.78, 133.72, 133.47, 131.50, 129.66, 129.38, 129.12, 128.41, 128.27, 127.45, 127.35, 127.01, 126.94, 120.24, 120.02, 119.74, 109.76, 108.91, 107.44, 105.94, 61.46, 52.43, 50.41, 28.57. **HRMS (*m/z*)** calcd. for $C_{36}H_{32}N_6O_3Cl$ [M+H]$^+$, 631.2218; found, 631.2218.

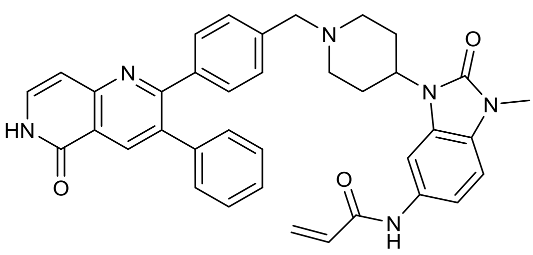

24b

Synthesis of N-(1-methyl-2-oxo-3-(1-(4-(5-oxo-3-phenyl-5,6-dihydro-1,6-naphthyridin-2-yl)benzyl)-piperidin-4-yl)-2,3-dihydro-1H-benzo[d]imidazol-5-yl)acrylamide (**24b**)**.** *2-(4-((4-(6-amino-3-methyl-2-oxo-2,3-dihydro-1H-benzo[d]imidazol-1-yl)piperidin-1-yl)-methyl)phenyl)-3-phenyl-1,6-naphthyridin-5(6H)-one* (**23b**, 40 mg, 0.1 mmol) was used following Common Procedure G and yielded the title product as a white solid (23 mg, 0.04 mmol, 52 %). **^{1}H-NMR (600 MHz, DMSO-*d*$_6$)** δ 11.58 (d, *J* = 5.4 Hz, 1H), 10.12 (s, 1H), 8.39 (s, 1H), 7.73 (s, 1H), 7.49 (t, *J* = 6.6 Hz, 1H), 7.42 – 7.30 (m, 6H), 7.31 – 7.23 (m, 4H), 7.09 (d, *J* = 8.4 Hz, 1H), 6.69 (d, *J* = 7.3 Hz, 1H), 6.41 (dt, *J* = 24.2, 12.1 Hz, 1H), 6.25 (d, *J* = 16.7 Hz, 1H), 5.74 (t, *J* = 10.5 Hz, 1H), 4.14 (q, *J* = 12.3 Hz, 1H), 3.54 (s, 2H), 3.29 (s, 3H), 2.94 (d, *J* = 10.3 Hz, 2H), 2.31 (dd, *J* = 22.0, 11.2 Hz, 2H), 2.10 (t, *J* = 11.3 Hz, 2H), 1.66 (d, *J* = 10.6 Hz, 2H). **^{13}C-NMR (151 MHz, DMSO-*d*$_6$)** δ 162.82, 161.99, 161.19, 153.26, 152.92, 139.02, 138.70, 138.20, 136.79, 133.73, 133.50, 133.03, 132.00, 129.67, 129.38, 128.43, 128.33, 127.71, 127.48, 126.44, 126.00, 120.25, 112.09, 107.69, 105.92, 100.91, 61.45, 52.53, 50.76, 28.70, 26.87. **HRMS (*m/z*)** calcd. for $C_{37}H_{35}N_6O_3$ $[M+H]^+$, 611.2765; found, 611.2759.

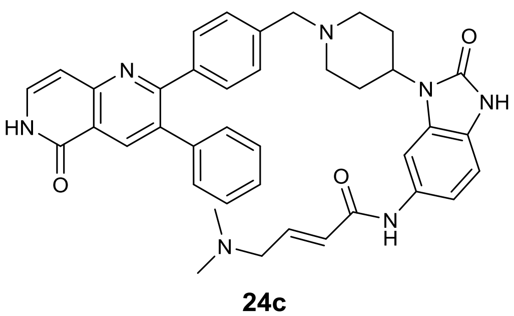

24c

Synthesis of (E)-4-(dimethylamino)-N-(2-oxo-3-(1-(4-(5-oxo-3-phenyl-5,6-dihydro-1,6-naphthyridin-2-yl)benzyl)piperidin-4-yl)-2,3-dihydro-1H-benzo[d]imidazol-5-yl)but-2-en amide (**24c**)**.** (*E*)-4-(dimethyl-amino)but-2-enoic acid (92 mg, 0.6 mmol, 6 eq.) was dissolved in 2 mL DCM and oxalyl chloride (190 µL, 2.2 mmol, 24 eq.) was added slowly. The reaction mixture was allowed to stir ovn at rt.

Subsequently, the solvents were evaporated and the resulting solid was dissolved in 5 mL THF. 2-(4-((4-(6-amino-2-oxo-2,3-dihydro-1*H*-benzo[*d*]imidazol-1-yl)piperidin-1-yl)-methyl)phenyl)-3-phenyl-1,6-naphthyridin-5(6*H*)-one (**23**, 50 mg, 0.1 mmol) and K_2CO_3 (102 mg, 0.7 mmol, 8 eq.) were added an the mixture was allowed to stir ovn at rt. The solvents were evaporated *in vacuo* and silica gel column chromatography (10 % MeOH/DCM + 1 % NH_3) yielded the desired product as a white solid (20 mg, 0.03 mmol, 30 %). **^{1}H-NMR (500 MHz, DMSO-*d*$_6$)** δ 11.61 (d, *J* = 5.3 Hz, 1H), 10.81 (s, 1H), 10.08 (s, 1H), 8.38 (s, 1H), 7.73 (s, 1H), 7.52 – 7.47 (m, 1H), 7.36 – 7.30 (m, 5H), 7.26 (dd, *J* = 15.2, 6.9 Hz, 5H), 6.90 (d, *J* = 8.4 Hz, 1H), 6.74 – 6.68 (m, 2H), 6.27 (d, *J* = 15.4 Hz, 1H), 4.14 – 4.07 (m, 1H), 3.54 (s, 2H), 3.07 (d, *J* = 5.5 Hz, 2H), 2.94 (d, *J* = 10.8 Hz, 2H), 2.30 (td, *J* = 12.2, 9.1 Hz, 2H), 2.19 (s, 6H), 2.08 (t, *J* = 11.4 Hz, 2H), 1.64 (d, *J* = 10.3 Hz, 2H). **^{13}C-NMR (126 MHz, DMSO-*d*$_6$)** δ 162.83, 161.98, 161.20, 153.99, 152.93, 140.52, 139.03, 138.75, 138.19, 136.79, 133.73, 133.50, 132.89, 129.67, 129.39, 128.94, 128.43, 128.31, 127.47, 126.34, 124.33, 120.24, 112.02, 108.61, 105.92, 100.84, 61.47, 59.72, 52.60, 50.10, 45.07, 28.66. **HRMS (*m/z*)** calcd: 654.3187 for $C_{39}H_{39}N_7O_3$ $[M+H]^+$. found: 654.3178.

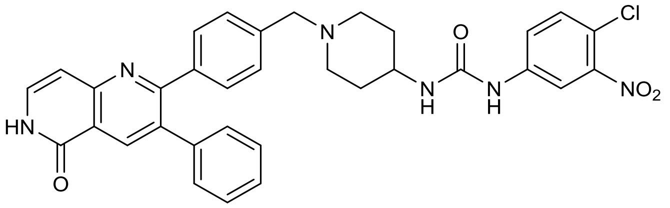

25

Synthesis of 1-(4-chloro-3-nitrophenyl)-3-(1-(4-(5-oxo-3-phenyl-5,6-dihydro-1,6-naphthyridin-2-yl)benzyl)piperidin-4-yl)urea (**25**). 4-(5-oxo-3-phenyl-5,6-dihydro-1,6-naphthyridin-2-yl)benzaldehyde (**9**, 300 mg, 0.9 mmol) and 1-(4-chloro-3-nitrophenyl)-3-(piperidin-4-yl)urea (**17**, 1.3 eq., 348 mg, 1.2 mmol) were used following Common Procedure E and yielded the title product as a yellow solid (95 mg, 0.2 mmol, 17 %). **^{1}H-NMR (500 MHz, DMSO-*d*$_6$)** δ 11.60 (d, 1H, *J* = 4.3 Hz), 8.96 (s, 1H), 8.37 (d, 1H, *J* = 5.4 Hz), 8.25 (d, 1H, *J* = 2.5 Hz), 7.58 (d, 1H, *J* = 8.9 Hz), 7.54-7.47 (m, 2H), 7.36-7.27 (m, 5H), 7.26-7.18 (m, 4H), 6.68 (d, 1H, *J* = 7.3 Hz), 6.39 (d, 1H, *J* = 7.6 Hz), 3.51-3.41 (m, 3H), 2.68 (d, 2H, *J* = 9.9 Hz), 2.04 (t, 2H, *J* = 10.2 Hz), 1.78 (d, 2H, *J* = 10.1 Hz), 1.46-1.35 (m, 2H). **^{13}C-NMR (126 MHz, DMSO-**

***d*₆)** δ 161.66, 154.49, 147.92, 141.06, 139.39, 138.56, 137.27, 133.98, 132.12, 130.10, 129.84, 128.89, 128.67, 127.92, 122.85, 116.16, 113.96, 106.37, 62.18, 52.16, 49.07, 32.28. **HRMS (*m/z*)** calcd. for $C_{33}H_{30}N_6O_4Cl$ [M+H]$^+$, 609.2011; found, 609.2026.

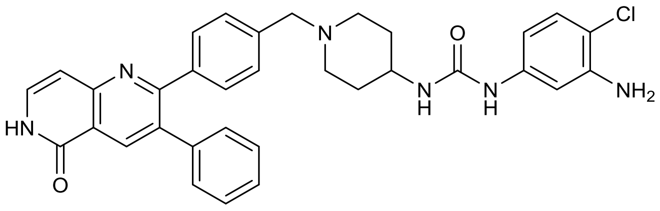

26

Synthesis of 1-(3-amino-4-chlorophenyl)-3-(1-(4-(5-oxo-3-phenyl-5,6-dihydro-1,6-naphthyridin-2-yl)benzyl)piperidin-4-yl)urea (**26**)**.** 1-(4-chloro-3-nitrophenyl)-3-(1-(4-(5-oxo-3-phenyl-5,6-dihydro-1,6-naphthyridin-2-yl)benzyl)piperidin-4-yl)urea (**25**, 93 mg, 0.2 mmol) was used following Common Procedure D and yielded the title product as yellow solid (88 mg, 0.2 mmol, quantitative). **^{1}H-NMR (500 MHz, DMSO-*d*₆)** δ 11.61 (d, *J* = 5.0 Hz, 1H), 8.39 (s, 1H), 7.53-7.47 (m, 1H), 7.36-7.28 (m, 6H), 7.28-7.20 (m, 4H), 6.97 (d, *J* = 8.6 Hz, 1H), 6.9 (d, 1H, *J* = 2.3 Hz), 6.68 (d, 1H, *J* = 7.3 Hz), 6.55 (dd, *J* = 8.7, 2.3 Hz, 1H), 6.15 (s, 1H), 5.21 (s, 2H), 3.56 (s, 1H), 3.46 (m, 2H), 2.75 (s, 2H), 1.81 (d, *J* = 10.1 Hz, 2H), 1.42 (m, 2H), 1.24 (m, 2H). **^{13}C-NMR (126 MHz, DMSO-*d*₆)** δ 162.46, 154.85, 153.42, 145.09, 140.47, 139.44, 138.01, 138.43, 137.35, 134.19, 134.04, 130.17, 129.89, 129.23. 128.9, 128.31, 120.7, 110.09, 107.42, 106.45, 104.82, 74, 70.04, 64.38, 25.67. **HRMS (*m/z*)** calcd.: 579.22698 for $C_{33}H_{32}ClN_6O_2$ [M+H]$^+$, found: 579.22680.

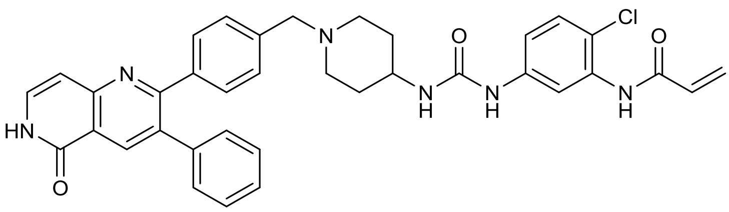

27

Synthesis of N-(2-chloro-5-(3-(1-(4-(5-oxo-3-phenyl-5,6-dihydro-1,6-naphthyridin-2-yl)benzyl)piperidin-4-yl)ureido)phenyl)acrylamide (**27**)**.** 1-(3-amino-4-chlorophenyl)-3-(1-(4-(5-oxo-3-phenyl-5,6-dihydro-

1,6-naphthyridin-2-yl)benzyl)piperidin-4-yl)urea (**26**, 75 mg, 0.1 mmol) was used following Common Procedure G and yielded the title product as a pale yellow solid (48 mg, 0.1 mmol, 58 %). **^{1}H-NMR (700 MHz, DMSO-*d*$_6$)** δ 11.57 (s, 1H), 9.59 (s, 1H), 8.57 (s, 1H), 8.38 (s, 1H), 7.83 (s, 1H), 7.51 – 7.48 (m, 1H), 7.34 – 7.29 (m, 8H), 7.26 – 7.20 (m, 4H), 6.68 (d, *J* = 6.0 Hz, 1H), 6.59 (dt, *J* = 17.2, 8.7 Hz, 1H), 6.26 (dd, *J* = 17.0, 1.9 Hz, 1H), 3.45 (b, 3H), 2.65 (dd, *J* = 17.1, 9.9 Hz, 2H), 2.09 – 2.00 (b, 2H), 1.77 (d, *J* = 20.7 Hz, 2H), 1.42 – 1.35 (b, 2H). **^{13}C-NMR (176 MHz, DMSO-*d*$_6$)** δ 163.37, 161.99, 161.20, 154.27, 152.92, 139.69, 139.02, 138.12, 136.79, 134.62, 133.72, 133.51, 131.45, 129.65, 129.38, 129.26, 128.42, 128.21, 127.46, 127.31, 120.26, 117.98, 115.54, 114.90, 105.90, 61.72, 51.67, 46.16, 32.06. **HRMS (*m/z*)** calcd.: 633.2375 for $C_{36}H_{34}ClN_6O_3$ [M+H]$^+$, found: 633.2375.

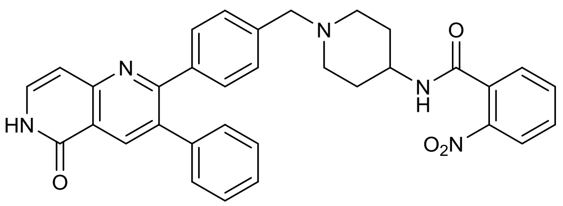

28a

Synthesis of 2-nitro-N-(1-(4-(5-oxo-3-phenyl-5,6-dihydro-1,6-naphthyridin-2-yl)benzyl)piperidin-4-yl)-benzamide (**28a**). 4-(5-oxo-3-phenyl-5,6-dihydro-1,6-naphthyridin-2-yl)benzaldehyde (**9**, 350 mg, 1.1 mmol) and 2-nitro-*N*-(piperidin-4-yl)benzamide (**21a**, 1.3 eq., 348 mg, 1.4 mmol) were used following Common Procedure E and yielded the title product as a pale yellow solid (209 mg, 0.4 mmol, 35 %). **^{1}H NMR (500 MHz, DMSO-*d*$_6$)** δ 11.59 (d, *J* = 5.4 Hz, 1H), 8.56 (d, *J* = 7.7 Hz, 1H), 8.39 (s, 1H), 8.05 (dd, *J* = 8.1, 0.9 Hz, 1H), 7.78 (td, *J* = 7.5, 1.1 Hz, 1H), 7.68 (td, *J* = 8.1, 1.4 Hz, 1H), 7.57 (dd, *J* = 7.6, 1.2 Hz, 1H), 7.50 (dd, *J* = 7.3, 6.0 Hz, 1H), 7.35 – 7.30 (m, 5H), 7.26 – 7.21 (m, 4H), 6.69 (d, *J* = 7.3 Hz, 1H), 3.74 – 3.66 (m, 1H), 3.46 (s, 2H), 2.76 (d, *J* = 11.3 Hz, 2H), 2.04 (t, *J* = 10.8 Hz, 2H), 1.82 (d, *J* = 9.8 Hz, 2H), 1.52 – 1.44 (m, 2H).**^{13}C NMR (126 MHz, DMSO-*d*$_6$)** δ 164.82, 161.99, 161.20, 152.91, 146.81, 139.09, 139.03, 138.08, 136.78, 133.71, 133.66, 133.50, 132.93, 130.47, 129.63, 129.37, 129.11, 128.41, 128.14, 127.45, 124.00, 120.23, 105.90, 61.68, 51.85, 46.65, 31.13. **LCMS (*m/z*)** calcd.: for $C_{33}H_{29}N_5O_4$ [M+H]$^+$, 560.22; found, 560.31.

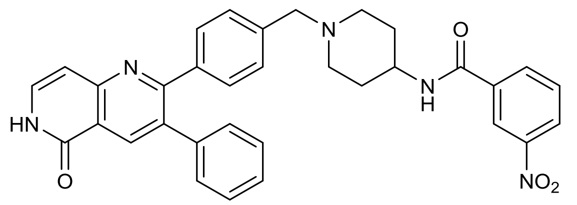

28b

Synthesis of 3-nitro-N-(1-(4-(5-oxo-3-phenyl-5,6-dihydro-1,6-naphthyridin-2-yl)benzyl)piperidin-4-yl)benzamide (**28b**). 4-(5-oxo-3-phenyl-5,6-dihydro-1,6-naphthyridin-2-yl)benzaldehyde (**9**, 400 mg, 1.2 mmol) and 3-nitro-*N*-(piperidin-4-yl)benzamide (**21b**, 1.3 eq., 397 mg, 1.6 mmol) were used following Common Procedure E and yielded the title product as a pale yellow solid (476 mg, 0.9 mmol, 69 %). **^{1}H NMR (500 MHz, $CDCl_3$)** δ 10.37 (s, 1H), 8.60 (s, 1H), 8.50 (t, *J* = 1.8 Hz, 1H), 8.26 (dd, *J* = 8.2, 1.2 Hz, 1H), 8.07 (d, *J* = 7.8 Hz, 1H), 7.55 (t, *J* = 8.0 Hz, 1H), 7.33 (d, *J* = 8.2 Hz, 2H), 7.27 (dd, *J* = 7.1, 4.5 Hz, 1H), 7.23 – 7.20 (m, 3H), 7.19 (s, 2H), 7.18 – 7.14 (m, 2H), 6.84 (d, *J* = 7.4 Hz, 1H), 3.96 (ddd, *J* = 14.6, 11.2, 5.8 Hz, 1H), 3.46 (s, 2H), 2.80 (d, *J* = 11.3 Hz, 2H), 2.12 (t, *J* = 11.0 Hz, 2H), 1.97 (d, *J* = 9.5 Hz, 2H), 1.55 (d, *J* = 10.6 Hz, 2H). **^{13}C NMR (126 MHz, $CDCl_3$)** δ 164.39, 163.80, 162.51, 153.22, 148.21, 139.07, 137.61, 136.41, 135.06, 133.16, 131.13, 129.95, 129.81, 129.65, 128.80, 128.34, 127.54, 125.97, 121.70, 120.76, 108.49, 62.53, 52.18, 47.61, 32.13. **HRMS (m/z)** calcd. for $C_{33}H_{30}N_5O_4$ $[M+H]^+$, 560.2286; found, 560.2292.

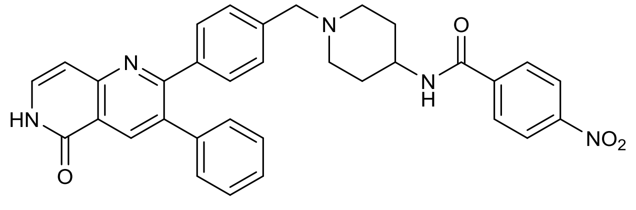

28c

Synthesis of 4-nitro-N-(1-(4-(5-oxo-3-phenyl-5,6-dihydro-1,6-naphthyridin-2-yl)ben-zyl)piperidin-4-yl)benzamide (**28c**). 4-(5-oxo-3-phenyl-5,6-dihydro-1,6-naphthyridin-2-yl)benzaldehyde (**9**, 350 mg, 1.1 mmol) and 4-nitro-*N*-(piperidin-4-yl)benzamide (**21c**, 1.3 eq., 348 mg, 1.4 mmol) were used following Common Procedure E and yielded the title product as a pale yellow solid (261 mg, 0.5 mmol, 44 %). **^{1}H NMR (500 MHz, DMSO-d_6)** δ 11.62 (d, *J* = 5.4 Hz, 1H), 8.63 (d, *J* = 7.6 Hz, 1H), 8.38 (s, 1H),

8.31 (d, *J* = 8.8 Hz, 2H), 8.06 (d, *J* = 8.8 Hz, 2H), 7.53 – 7.48 (m, 1H), 7.35 – 7.28 (m, 5H), 7.23 (dd, *J* = 12.2, 5.6 Hz, 4H), 6.68 (d, *J* = 7.3 Hz, 1H), 3.77 (qd, *J* = 11.3, 5.7 Hz, 1H), 3.47 (s, 2H), 2.80 (d, *J* = 11.4 Hz, 2H), 2.02 (t, *J* = 11.1 Hz, 2H), 1.79 (d, *J* = 10.1 Hz, 2H), 1.57 (qd, *J* = 12.1, 3.3 Hz, 2H). **^{13}C NMR (126 MHz, DMSO-*d*$_6$)** δ 164.03, 161.99, 161.15, 152.92, 148.92, 140.33, 138.99, 136.80, 133.72, 133.53, 129.68, 129.39, 128.81, 128.41, 128.24, 127.46, 123.43, 120.27, 105.88, 61.57, 52.06, 47.20, 31.36. **LCMS (*m/z*)** calcd.: for $C_{33}H_{29}N_5O_4$ $[M+H]^+$, 560.22; found, 560.29.

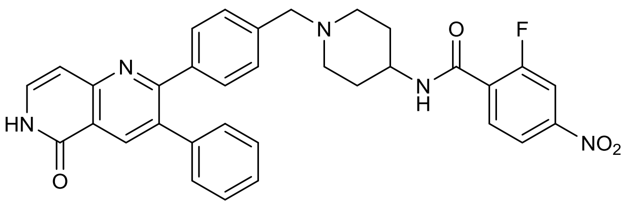

28d

Synthesis of 2-fluoro-4-nitro-N-(1-(4-(5-oxo-3-phenyl-5,6-dihydro-1,6-naphthyridin-2-yl)ben-zyl)piperidin-4-yl)benzamide (**28d**). 4-(5-oxo-3-phenyl-5,6-dihydro-1,6-naphthyridin-2-yl)benzaldehyde (**9**, 350 mg, 1.1 mmol) and 2-fluoro-4-nitro-*N*-(piperidin-4-yl)benzamide (**21d**, 1.3 eq., 373 mg, 1.4 mmol) were used following Common Procedure E and yielded the title product as a pale yellow solid (293 mg, 0.5 mmol, 47 %). **^{1}H NMR (500 MHz, DMSO-*d*$_6$)** δ 11.61 (d, *J* = 5.3 Hz, 1H), 8.62 (d, *J* = 7.6 Hz, 1H), 8.38 (s, 1H), 8.20 (dd, *J* = 9.5, 1.9 Hz, 1H), 8.12 (dd, *J* = 8.5 Hz, 1.8 Hz, 1H), 7.80 – 7.75 (m, 1H), 7.52 – 7.47 (m, 1H), 7.31 (dd, *J* = 4.9, 3.3 Hz, 5H), 7.27 – 7.19 (m, 4H), 6.68 (d, *J* = 7.3 Hz, 1H), 3.80 – 3.70 (m, 1H), 3.48 – 3.41 (m, 2H), 2.75 (d, *J* = 11.1 Hz, 2H), 2.03 (t, *J* = 10.9 Hz, 2H), 1.80 (d, *J* = 10.3 Hz, 2H), 1.50 (dd, *J* = 20.4 Hz, 11.0 Hz, 2H). **^{13}C NMR (126 MHz, DMSO-*d*$_6$)** δ 162.02, 161.75, 161.22, 159.18, 157.51, 152.94, 148.94, 148.88, 139.05, 138.12, 136.82, 133.73, 133.54, 131.15, 131.04, 130.97, 129.67, 129.40, 128.44, 128.20, 127.48, 120.26, 119.60, 112.08, 111.90, 105.92, 61.68, 56.04, 51.84, 46.94, 31.28, 18.59. **LCMS (*m/z*)** calcd.: for $C_{33}H_{28}FN_5O_4$ $[M+H]^+$, 578.21; found, 578.41.

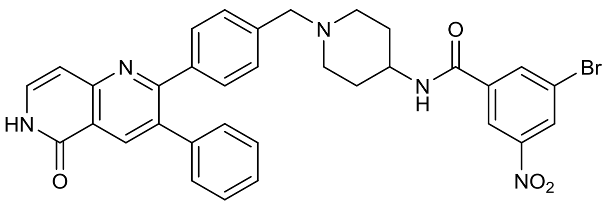

28e

Synthesis of 3-bromo-5-nitro-N-(1-(4-(5-oxo-3-phenyl-5,6-dihydro-1,6-naphthyridin-2-yl)benzyl)-piperidin-4-yl)benzamide (**28e**). 4-(5-oxo-3-phenyl-5,6-dihydro-1,6-naph-thyridin-2-yl)benzaldehyde (**9**, 544 mg, 1.7 mmol) and 3-bromo-5-nitro-*N*-(piperidin-4-yl)benzamide (**21e**, 1.3 eq., 711 mg, 2.2 mmol) were used following Common Procedure E and yielded the title product as a pale yellow solid (418 mg, 0.7 mmol, 39 %). **^{1}H NMR (500 MHz, DMSO-*d*$_6$)** δ 11.61 (d, *J* = 5.7 Hz, 1H), 8.78 (d, *J* = 7.4 Hz, 1H), 8.66 – 8.63 (m, 1H), 8.54 (t, *J* = 1.9 Hz, 1H), 8.47 (t, *J* = 1.6 Hz, 1H), 8.39 (s, 1H), 7.50 (dd, *J* = 7.3 Hz, 6.0 Hz, 1H), 7.36 – 7.22 (m, 8H), 6.68 (d, *J* = 7.2 Hz, 2H), 4.05 – 3.99 (m, 1H), 3.83 (d, *J* = 6.7 Hz, 1H), 3.65 (d, *J* = 24.1 Hz, 1H), 2.90 (d, *J* = 7.6 Hz, 1H), 2.22 (s, 1H), 1.88 – 1.81 (m, 1H), 1.75 (s, 4H), 1.68 – 1.59 (m, 1H). **^{13}C NMR (126 MHz, DMSO-*d*$_6$)** δ 171.44, 162.00, 161.22, 152.95, 148.56, 139.05, 138.11, 137.44, 136.78, 136.10, 133.67, 129.63, 129.37, 128.39, 128.13, 127.42, 122.09, 121.38, 120.25, 105.80, 61.60, 52.08, 47.49, 31.30, 22.50. **LCMS (*m/z*)** calcd.: for $C_{33}H_{28}BrN_5O_4$ [M+H]$^+$, 638.13; found, 638.29.

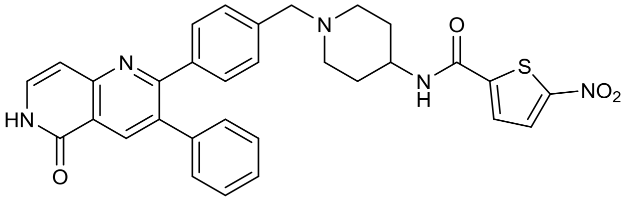

28f

Synthesis of 5-nitro-N-(1-(4-(5-oxo-3-phenyl-5,6-dihydro-1,6-naphthyridin-2-yl)benzyl)piperidin-4-yl)-thiophene-2-carboxamide (**28f**). 4-(5-oxo-3-phenyl-5,6-dihydro-1,6-naphthyridin-2-yl)benzaldehyde (**9**, 350 mg, 1.1 mmol) and 5-nitro-*N*-(piperidin-4-yl)-thiophene-2-carboxamide (**21f**, 1.3 eq., 356 mg, 1.4 mmol) were used following Common Procedure E and yielded the title product as a pale yellow solid (317 mg, 0.5 mmol, 52 %).**^{1}H NMR (500 MHz, DMSO-*d*$_6$)** δ 11.60 (d, *J* = 5.4 Hz, 1H), 8.76 (d, *J* = 7.7

Hz, 1H), 8.38 (s, 1H), 8.14 – 8.12 (m, 1H), 7.84 (d, *J* = 4.4 Hz, 1H), 7.50 (dd, *J* = 7.2, 6.0 Hz, 1H), 7.33 – 7.30 (m, 5H), 7.25 – 7.20 (m, 4H), 6.68 (d, *J* = 7.3 Hz, 1H), 3.76 – 3.69 (m, 1H), 3.47 (s, 2H), 2.80 (d, *J* = 11.5 Hz, 2H), 2.01 (t, *J* = 11.3 Hz, 2H), 1.79 (d, *J* = 10.0 Hz, 2H), 1.56 (qd, *J* = 12.2, 3.6 Hz, 2H).**^{13}C NMR (126 MHz, DMSO-*d*$_6$)** δ 163.31, 162.16, 161.35, 158.89, 153.08, 152.96, 146.86, 139.18, 139.07, 138.29, 136.96, 133.87, 133.69, 130.33, 129.81, 129.65, 129.55, 128.75, 128.58, 128.35, 127.62, 127.43, 120.40, 106.06, 61.68, 52.14, 47.59, 31.44. **LCMS (*m/z*)** calcd.: for $C_{31}H_{27}N_5O_4S$ [M+H]$^+$, 566.18; found, 566.39.

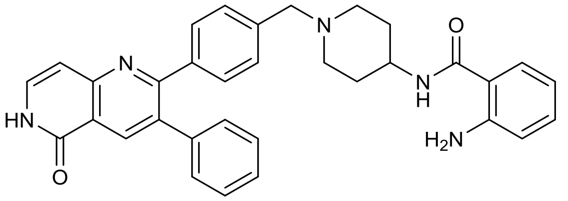

29a

Synthesis of 2-amino-N-(1-(4-(5-oxo-3-phenyl-5,6-dihydro-1,6-naphthyridin-2-yl)benzyl)-piperidin-4-yl)benzamide (**29a**). 2-nitro-*N*-(1-(4-(5-oxo-3-phenyl-5,6-dihydro-1,6-naphthyridin-2-yl)ben-zyl)-piperidin-4-yl)benzamide (**28a**, 590 mg, 1.1 mmol) was used following Common Procedure D and yielded the title product as a pale yellow solid (374 mg, 0.7 mmol, 67 %). **^{1}H NMR (500 MHz, DMSO-*d*$_6$)** δ 11.61 (s, 1H), 8.38 (s, 1H), 7.73 (d, *J* = 7.7 Hz, 1H), 7.66 – 7.46 (m, 4H), 7.31 (dd, *J* = 4.5 Hz, 3.0 Hz, 5H), 7.27 – 7.17 (m, 4H), 6.68 (t, *J* = 9.6 Hz, 1H), 6.51 (d, *J* = 8.4 Hz, 1H), 5.58 (s, 2H), 3.75 – 3.63 (m, 1H), 3.44 (s, 2H), 2.77 (d, *J* = 10.6 Hz, 2H), 1.98 (t, *J* = 11.0 Hz, 2H), 1.71 (d, *J* = 10.2 Hz, 2H), 1.53 (dd, *J* = 21.2 Hz, 10.4 Hz, 2H). **^{13}C NMR (126 MHz, DMSO-*d*$_6$)** δ 168.27, 162.04, 161.24, 152.95, 149.51, 139.16, 139.06, 138.10, 136.83, 133.74, 133.56, 131.53, 129.67, 129.42, 128.46, 128.28, 128.23, 127.50, 120.26, 116.20, 115.15, 114.49, 105.94, 61.75, 52.34, 46.39, 31.50. **LCMS (*m/z*)** calcd.: for $C_{33}H_{31}N_5O_2$ [M+H]$^+$, 530.25; found, 530.34.

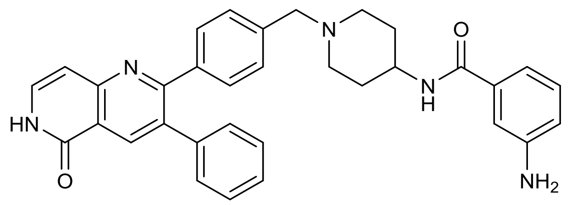

29b

Synthesis of 3-amino-N-(1-(4-(5-oxo-3-phenyl-5,6-dihydro-1,6-naphthyridin-2-yl)benzyl)-piperidin-4-yl)benzamide (**29b**). 3-nitro-*N*-(1-(4-(5-oxo-3-phenyl-5,6-dihydro-1,6-naphthyridin-2-yl)benzyl)-piperidin-4-yl)benzamide (**28b**, 357 mg, 0.6 mmol) was used following Common Procedure D and yielded the title product as a pale yellow solid (266 mg, 0.5 mmol, 79 %). **^{1}H NMR (700 MHz, DMSO-*d*$_6$)** δ 11.57 (d, *J* = 5.2 Hz, 1H), 8.38 (s, 1H), 8.15 (d, *J* = 7.7 Hz, 1H), 7.49 (dd, *J* = 7.2, 6.0 Hz, 1H), 7.34 – 7.30 (m, 5H), 7.26 – 7.19 (m, 5H), 7.07 (t, *J* = 1.5 Hz, 1H), 6.97 (dd, *J* = 5.8, 4.0 Hz, 1H), 6.83 (t, *J* = 1.9 Hz, 1H), 6.68 (d, *J* = 7.3 Hz, 1H), 5.55 (s, 2H), 3.73 – 3.67 (m, 1H), 3.44 (s, 2H), 2.77 (d, *J* = 11.4 Hz, 2H), 1.99 (t, *J* = 11.1 Hz, 2H), 1.72 (d, *J* = 10.2 Hz, 2H), 1.54 (ddd, *J* = 15.7, 12.6, 3.8 Hz, 2H). **^{13}C NMR (176 MHz, DMSO-*d*$_6$)** δ 165.36, 162.47, 161.68, 153.39, 150.84, 139.58, 139.51, 138.56, 138.02, 137.25, 134.19, 133.97, 130.09, 129.85, 128.88, 128.64, 127.92, 122.23, 120.71, 118.32, 116.79, 112.56, 106.38, 62.14, 52.70, 47.37, 31.86. **HRMS (*m/z*)** calcd. for $C_{33}H_{31}N_5O_2$ [M+H]$^+$, 530.2544; found, 530.2550.

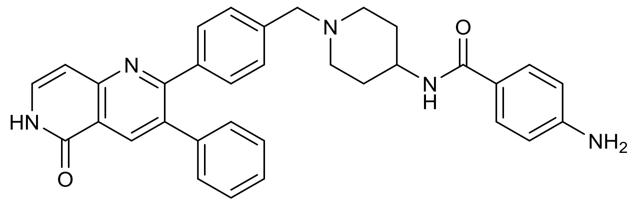

29c

Synthesis of 4-amino-N-(1-(4-(5-oxo-3-phenyl-5,6-dihydro-1,6-naphthyridin-2-yl)ben-zyl)-piperidin-4-yl)benzamide (**29c**). 4-nitro-*N*-(1-(4-(5-oxo-3-phenyl-5,6-dihydro-1,6-naphthyridin-2-yl)ben-zyl)pi-peridin-4-yl)benzamide (**28c**, 201 mg, 0.4 mmol) was used following Common Procedure D and yielded the title product as a yellow solid (132 mg, 0.3 mmol, 69 %). **^{1}H NMR (500 MHz, DMSO-*d*$_6$)** δ 11.91 (s, 1H), 8.38 (s, 1H), 7.96 (t, *J* = 13.7 Hz, 1H), 7.49 (dd, *J* = 40.6 Hz, 6.6 Hz, 2H), 7.32 (d, *J* = 4.9 Hz, 5H), 7.23 (dd, *J* = 13.8 Hz, 6.0 Hz, 4H), 7.11 (t, *J* = 7.2 Hz, 1H), 6.67 (t, *J* = 8.3 Hz, 2H), 6.49 (t, *J* = 7.2 Hz, 1H), 6.31

(s, 2H), 3.71 (s, 1H), 3.45 (s, 2H), 3.16 (s, 4H), 2.78 (d, *J* = 9.9 Hz, 2H), 1.99 (t, *J* = 10.7 Hz, 2H). **^{13}C NMR (126 MHz, DMSO-*d*$_6$)** δ 168.24, 162.07, 161.15, 152.95, 149.47, 139.09, 138.10, 136.79, 133.70, 131.46, 129.62, 129.38, 128.40, 128.20, 127.43, 120.23, 116.17, 115.15, 114.45, 105.77, 61.71, 52.30, 48.58, 31.46. **LCMS (*m/z*)** calcd.: for $C_{33}H_{31}N_5O_2$ [M+H]$^+$, 530.25; found, 530.29.

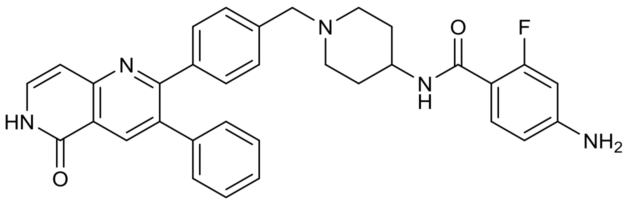

29d

Synthesis of 4-amino-2-fluoro-N-(1-(4-(5-oxo-3-phenyl-5,6-dihydro-1,6-naphthyridin-2-yl)benzyl)-piperidin-4-yl)benzamide (**29d**). 2-fluoro-4-nitro-*N*-(1-(4-(5-oxo-3-phenyl-5,6-dihydro-1,6-naph-thyridin-2-yl)benzyl)piperidin-4-yl)benzamide (**28d**, 250 mg, 0.4 mmol) was used following Common Procedure D. The crude product was used without further purification. **^{1}H NMR (500 MHz, DMSO-*d*$_6$)** δ 11.58 (d, *J* = 4.9 Hz, 1H), 8.37 (d, *J* = 7.4 Hz, 1H), 7.49 (dd, *J* = 7.1, 5.9 Hz, 1H), 7.37 (dd, *J* = 11.6 Hz, 5.7 Hz, 2H), 7.33 – 7.28 (m, 5H), 7.23 (dt, *J* =5.2, 3.6 Hz, 2H), 7.20 (d, *J* = 8.1 Hz, 2H), 6.68 (d, *J* = 7.3 Hz, 1H), 6.38 (dd, *J* = 8.5, 2.1 Hz, 1H), 6.28 (dd, *J* = 14.1, 2.0 Hz, 1H), 5.87 (s, 2H), 3.76 – 3.67 (m, 1H), 3.44 (s, 2H), 2.72 (d, *J* = 8.8 Hz, 2H), 2.01 (s, 2H), 1.75 (d, *J* = 10.1 Hz, 2H), 1.55 – 1.45 (m, 2H). **^{13}C NMR (126 MHz, DMSO-*d*$_6$)** δ 162.98 (d, J = 2.1 Hz), 162.00, 161.92, 161.19, 160.53, 153.21, 153.14, 152.91, 139.02, 138.08, 136.78, 133.70, 133.46, 131.52 (d, *J* = 4.8 Hz), 129.61, 129.36, 128.39, 128.18, 127.44, 120.24, 109.55, 109.48, 109.38, 105.92, 99.30, 99.15, 61.70, 51.77 (d, *J* = 86.2 Hz), 46.18 (d, *J* = 76.4 Hz), 31.18 (d, *J* = 97.7 Hz). **LCMS (*m/z*)** calcd.: for $C_{33}H_{30}FN_5O_2$ [M+H]$^+$, 548.24; found, 548.47.

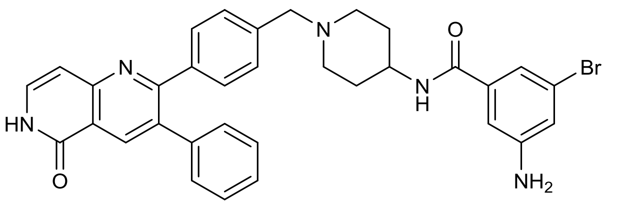

29e

Synthesis of 3-amino-5-bromo-N-(1-(4-(5-oxo-3-phenyl-5,6-dihydro-1,6-naphthyridin-2-yl)benzyl)-piperidin-4-yl)benzamide (**29e**). 3-nitro-5-bromo-*N*-(1-(4-(5-oxo-3-phenyl-5,6-dihydro-1,6-naphthyridin-2-yl)benzyl)piperidin-4-yl)benzamide (**28e**, 95 mg, 0.2 mmol) was used following Common Procedure D and yielded the title product as a yellow solid (70 mg, 0.1 mmol, 77 %). **^{1}H NMR (700 MHz, DMSO-*d*$_6$)** δ 11.57 (d, *J* = 5.2 Hz, 1H), 8.38 (s, 1H), 8.15 (d, *J* = 7.7 Hz, 1H), 7.49 (dd, *J* = 7.2 Hz, 6.0 Hz, 1H), 7.33 – 7.30 (m, 5H), 7.24 (dt, *J* = 4.9 Hz, 3.9 Hz, 2H), 7.21 (d, *J* = 8.1 Hz, 2H), 7.07 (t, *J* = 1.5 Hz, 1H), 6.98 (t, 1H), 6.83 (t, *J* = 1.9 Hz, 1H), 6.68 (d, *J* = 7.3 Hz, 1H), 5.55 (s, 2H), 3.73 – 3.58 (m, 1H), 3.44 (s, 2H), 2.77 (d, *J* = 11.4 Hz, 2H), 1.99 (t, *J* = 11.1 Hz, 2H), 1.72 (d, *J* = 10.2 Hz, 2H), 1.57 – 1.50 (m, 2H). **^{13}C NMR (176 MHz, DMSO-*d*$_6$)** δ 164.88, 161.98, 161.20, 152.91, 150.35, 139.10, 139.02, 138.07, 137.53, 136.77, 133.71, 133.48, 129.61, 129.36, 128.40, 128.16, 127.44, 121.75, 120.23, 117.83, 116.31, 112.07, 105.90, 61.66, 52.21, 46.88, 31.37. **LCMS (*m/z*)** calcd.: for $C_{33}H_{30}BrN_5O_2$ [M+H]$^+$, 608.16; found, 608.30.

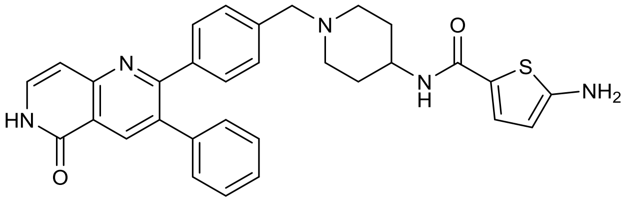

29f

Synthesis of 5-amino-N-(1-(4-(5-oxo-3-phenyl-5,6-dihydro-1,6-naphthyridin-2-yl)benzyl)piperidin-4-yl)thiophene-2-carboxamide (**29f**). 5-nitro-*N*-(1-(4-(5-oxo-3-phenyl-5,6-dihydro-1,6-naphthyridin-2-yl)benzyl)piperidin-4-yl)thiophene-2-carboxamide (**28f**, 250 mg, 0.4 mmol) was used following Common Procedure D. The crude product was used without further purification. **LCMS (*m/z*)** calcd.: for $C_{31}H_{29}N_5O$ [M+H]$^+$, 536.20; found, 536.31.

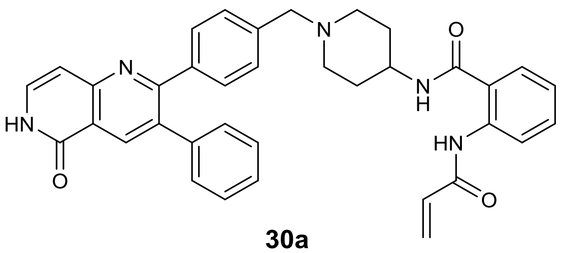

30a

Synthesis of 2-acrylamido-N-(1-(4-(5-oxo-3-phenyl-5,6-dihydro-1,6-naphthyridin-2-yl)-benzyl)-piperidin-4-yl)benzamide (**30a**). 2-amino-N-(1-(4-(5-oxo-3-phenyl-5,6-dihydro-1,6-naphthyridin-2-yl)-benzyl)-piperidin-4-yl)benzamide (**29a**, 150 mg, 0.3 mmol) was used following Common Procedure G and yielded the title product as a pale yellow solid (68 mg, 0.1 mmol, 41 %). **^{1}H NMR (500 MHz, DMSO-*d*$_6$)** δ 11.61 (d, *J* = 5.3 Hz, 1H), 11.47 (s, 1H), 8.57 (d, *J* = 7.3 Hz, 1H), 8.44 – 8.36 (m, 2H), 7.77 – 7.71 (m, 1H), 7.50 (t, *J* = 6.8 Hz, 2H), 7.32 (d, *J* = 6.3 Hz, 5H), 7.26 – 7.20 (m, 4H), 7.17 (t, *J* = 7.6 Hz, 1H), 6.68 (d, *J* = 7.3 Hz, 1H), 6.36 (dd, *J* = 17.0, 10.2 Hz, 1H), 6.22 (d, *J* = 17.0 Hz, 1H), 5.79 (d, *J* = 10.2 Hz, 1H), 3.78 (b, 1H), 3.46 (s, 2H), 2.78 (b, 2H), 2.01 (b, 2H), 1.79 (d, *J* = 10.6 Hz, 2H), 1.58 (d, *J* = 10.7 Hz, 2H). **^{13}C NMR (126 MHz, DMSO-*d*$_6$)** δ 167.55, 163.15, 162.03, 161.21, 152.95, 146.83, 139.05, 138.47, 138.13, 136.84, 133.73, 133.56, 132.60, 131.79, 129.70, 129.42, 128.46, 128.41, 128.24, 127.49, 127.14, 124.06, 123.02, 121.77, 120.83, 120.28, 105.94, 61.67, 52.16, 46.98, 31.19. **HRMS (*m/z*)** calcd.: 584.2656 for $C_{36}H_{33}N_5O_3$ $[M+H]^+$, found: 584.2647.

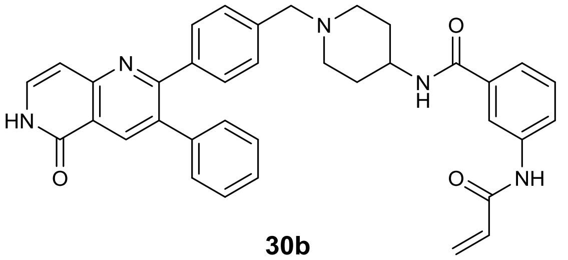

30b

Synthesis of 3-acrylamido-N-(1-(4-(5-oxo-3-phenyl-5,6-dihydro-1,6-naphthyridin-2-yl)-benzyl)-piperidin-4-yl)benzamide (**30b**). 3-amino-*N*-(1-(4-(5-oxo-3-phenyl-5,6-dihydro-1,6-naphthyridin-2-yl)-benzyl)-piperidin-4-yl)benzamide (**29b**, 150 mg, 0.3 mmol) was used following Common Procedure G and yielded the title product as a pale yellow solid (25 mg, 0.04 mmol, 15 %). **^{1}H NMR (600 MHz, DMSO-*d*$_6$)** δ 11.60 (d, *J* = 4.9 Hz, 1H), 10.33 (s, 1H), 8.38 (s, 1H), 8.25 (d, *J* = 4.5 Hz, 1H), 8.02 (s, 1H),

7.90 (t, *J* = 15.2 Hz, 1H), 7.57 – 7.46 (m, 2H), 7.39 (t, *J* = 7.9 Hz, 1H), 7.37 – 7.27 (m, 5H), 7.29 – 7.16 (m, 4H), 6.69 (d, *J* = 7.2 Hz, 1H), 6.45 (dt, *J* = 30.0, 15.0 Hz, 1H), 6.27 (dd, *J* = 17.0, 1.6 Hz, 1H), 5.75 (dt, *J* = 30.4, 15.2 Hz, 1H), 3.76 (b, 1H), 3.46 (s, *J* = 26.6 Hz, 2H), 2.78 (b, 2H), 2.05 – 1.98 (m, 2H), 1.76 (b, 2H), 1.57 (d, *J* = 8.5 Hz, 2H). **^{13}C NMR (151 MHz, DMSO-*d*$_6$)** δ 166.08, 163.73, 162.46, 161.66, 153.39, 139.47, 138.57, 137.26, 136.06, 134.19, 133.99, 132.23, 130.11, 129.85, 129.04, 128.88, 128.66, 127.93, 127.56, 122.51, 122.24, 120.72, 119.19, 106.37, 60.22, 52.71, 47.35, 31.92. HRMS (*m/z*): calcd. for $C_{36}H_{33}N_5O_3$ [M+H]$^+$: 584.2656; found: 584.2656.

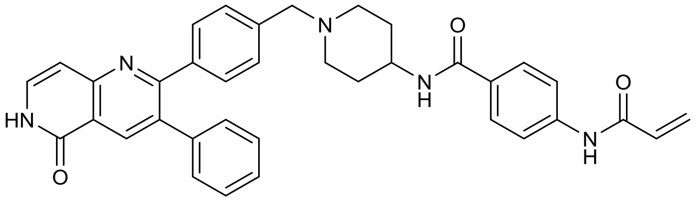

30c

Synthesis of 4-acrylamido-N-(1-(4-(5-oxo-3-phenyl-5,6-dihydro-1,6-naphthyridin-2-yl)-benzyl)-piperidin-4-yl)benzamide (**30c**). 4-amino-*N*-(1-(4-(5-oxo-3-phenyl-5,6-dihydro-1,6-naphthyridin-2-yl)-benzyl)-piperidin-4-yl)benzamide (**29c**, 347 mg, 0.3 mmol) was used following Common Procedure G and yielded the title product as a pale yellow solid (10 mg, 0.02 mmol, 6 %). **^{1}H NMR (600 MHz, DMSO-*d*$_6$)** δ 11.57 (d, *J* = 5.1 Hz, 1H), 11.46 (s, 1H), 8.54 (d, *J* = 7.6 Hz, 1H), 8.41 (d, *J* = 8.3 Hz, 1H), 8.38 (s, 1H), 7.74 (d, *J* = 7.7 Hz, 1H), 7.52 – 7.48 (m, 2H), 7.34 – 7.29 (m, 5H), 7.26 – 7.21 (m, 4H), 7.17 (t, *J* = 7.6 Hz, 1H), 6.68 (d, *J* = 7.3 Hz, 1H), 6.35 (dd, *J* = 17.0, 10.2 Hz, 1H), 6.22 (d, *J* = 17.0 Hz, 1H), 5.79 (d, *J* = 10.4 Hz, 1H), 3.82 – 3.75 (m, 1H), 3.46 (s, 2H), 2.79 (d, *J* = 11.0 Hz, 2H), 2.02 (t, *J* = 11.5 Hz, 2H), 1.79 (d, *J* = 11.1 Hz, 2H), 1.58 (dd, *J* = 21.0, 11.4 Hz, 2H). **^{13}C NMR (176 MHz, DMSO-*d*$_6$)** δ 167.99, 163.60, 162.46, 161.67, 153.39, 139.51, 138.93, 138.58, 137.26, 134.19, 133.98, 133.07, 132.21, 130.11, 129.85, 128.88, 128.83, 128.64, 127.92, 127.51, 123.44, 122.22, 121.26, 120.72, 106.37, 62.12, 52.60, 47.44, 31.65. **HRMS (*m/z*)** calcd.: 583.2583 for $C_{36}H_{33}N_5O_3$ [M+H]$^+$, found: 584.2655.

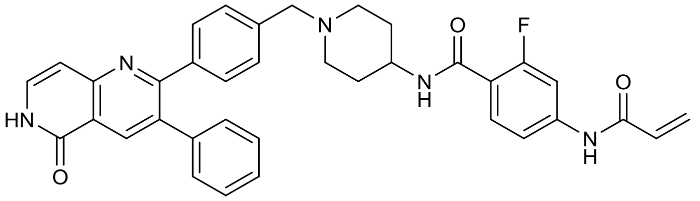

30d

Synthesis of 4-acrylamido-2-fluoro-N-(1-(4-(5-oxo-3-phenyl-5,6-dihydro-1,6-naphthyridin-2-yl)-benzyl)piperidin-4-yl)benzamide (**30d**). 4-amino-2-fluoro-*N*-(1-(4-(5-oxo-3-phenyl-5,6-dihydro-1,6-naphthyridin-2-yl)benzyl)piperidin-4-yl)benzamide (**29d**, 150 mg, 0.3 mmol) was used following Common Procedure G and yielded the title product as a pale yellow solid (40 mg, 0.1 mmol, 23 %). **^{1}H NMR (500 MHz, DMSO-*d*$_6$)** δ 11.63 – 11.58 (m, 1H), 10.54 (s, 1H), 8.38 (s, 1H), 8.05 (s, 1H), 7.74 (dd, *J* = 13.1, 1.6 Hz, 1H), 7.56 (t, *J* = 8.4 Hz, 1H), 7.52 – 7.46 (m, 1H), 7.37 (dd, *J* = 8.5, 1.6 Hz, 1H), 7.35 – 7.28 (m, 5H), 7.26 – 7.18 (m, 4H), 6.68 (d, *J* = 7.2 Hz, 1H), 6.43 (dd, *J* = 17.0, 10.1 Hz, 1H), 6.30 (dd, *J* = 17.0, 1.8 Hz, 1H), 5.82 (dd, *J* = 10.1, 1.8 Hz, 1H), 3.72 (b, 1H), 3.45 (s, 2H), 2.75 (d, *J* = 4.3 Hz, 2H), 2.00 (b, 2H), 1.77 (d, *J* = 6.0 Hz, 2H), 1.52 (d, *J* = 9.2 Hz, 2H). **^{13}C NMR (151 MHz, DMSO-*d*$_6$)** δ 163.61, 162.65, 161.99, 161.18, 160.06, 158.43, 152.92, 142.26 – 142.18, 139.01, 138.09, 136.79, 133.71, 133.51, 131.35, 130.61, 130.58, 129.64, 129.38, 128.41, 128.18, 128.01, 127.46, 120.25, 118.92 – 118.83, 114.65, 106.26 – 105.90, 61.68, 59.75, 51.97, 46.73, 31.35. **HRMS (*m/z*)** calcd.: 601.2489 for $C_{36}H_{32}FN_5O_3$ $[M+H]^+$, found: 602.2563.

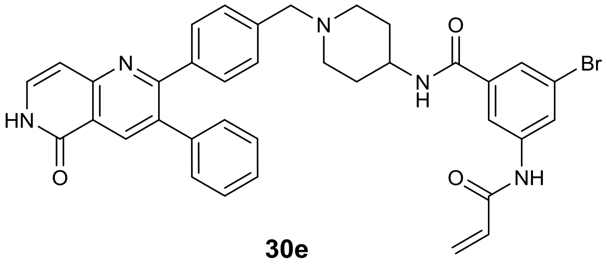

30e

Synthesis of 3-acrylamido-5-bromo-N-(1-(4-(5-oxo-3-phenyl-5,6-dihydro-1,6-naphthyridin-2-yl)-benzyl)piperidin-4-yl)benzamide (**30e**). 3-amino-5-bromo-*N*-(1-(4-(5-oxo-3-phenyl-5,6-dihydro-1,6-naphthyridin-2-yl)benzyl)piperidin-4-yl)benzamide (**29e**, 70 mg, 0.1 mmol) was used following Common Procedure G and yielded the title product as a pale yellow solid (42 mg, 0.06 mmol, 55 %). ^{1}H

NMR (500 MHz, DMSO-d_6) δ 11.62 (d, *J* = 5.5 Hz, 1H), 10.55 (s, 1H), 8.43 (d, *J* = 6.7 Hz, 1H), 8.39 (s, 1H), 8.24 (d, *J* = 1.6 Hz, 1H), 7.96 (s, 1H), 7.74 (s, 1H), 7.50 (dd, *J* = 7.1, 6.1 Hz, 1H), 7.36 – 7.30 (m, 5H), 7.24 (dd, *J* = 6.5, 2.9 Hz, 4H), 6.69 (d, *J* = 7.3 Hz, 1H), 6.44 (dd, *J* = 17.0, 10.1 Hz, 1H), 6.30 (dd, *J* = 17.0, 1.8 Hz, 1H), 5.81 (dd, *J* = 10.2, 1.7 Hz, 1H), 3.75 (b, 1H), 3.50 (s, 2H), 2.81 (b, 2H), 2.02 (b, 2H), 1.78 (d, *J* = 8.9 Hz, 2H), 1.58 (d, *J* = 7.9 Hz, 2H). **^{13}C NMR (126 MHz, DMSO-*d*$_6$)** δ 164.00, 163.57, 162.03, 161.20, 152.95, 140.53, 139.03, 137.26, 136.84, 133.74, 133.57, 131.39, 129.71, 129.42, 128.46, 128.27, 127.91, 127.51, 124.35, 123.79, 121.46, 120.28, 117.75, 105.94, 61.63, 52.15, 47.09, 31.34. **HRMS (*m/z*)** calcd.: 662.1689 for $C_{36}H_{32}BrN_5O_3$ [M+H]$^+$, found: 662.1763.

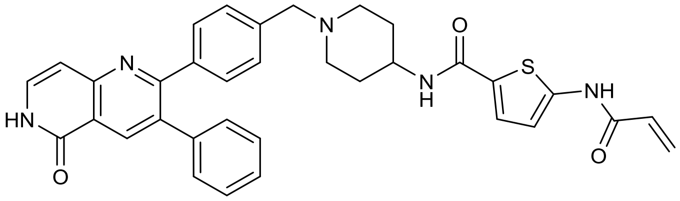

30f

Synthesis of 5-acrylamido-N-(1-(4-(5-oxo-3-phenyl-5,6-dihydro-1,6-naphthyridin-2-yl)-benzyl)-piperidin-4-yl)thiophene-2-carboxamide (**30f**). 5-amino-*N*-(1-(4-(5-oxo-3-phenyl-5,6-dihydro-1,6-naphthyridin-2-yl)ben-zyl)piperidin-4-yl)thiophene-2-carboxa-mide (**29f**, 150 mg, 0.3 mmol) was used following Common Procedure G and yielded the title product as a white solid (54 mg, 0.1 mmol, 31 %). **^{1}H NMR (500 MHz, DMSO-*d*$_6$)** δ 11.67 – 11.59 (m, 2H), 8.39 (s, 1H), 8.09 (d, *J* = 7.0 Hz, 1H), 7.58 (d, *J* = 4.1 Hz, 1H), 7.53 – 7.49 (m, 1H), 7.37 – 7.30 (m, 5H), 7.28 – 7.19 (m, 4H), 6.72 – 6.66 (m, 2H), 6.41 (dd, *J* = 17.0, 10.0 Hz, 1H), 6.32 (dd, *J* = 17.0, 1.8 Hz, 1H), 5.85 (dd, *J* = 10.0, 1.7 Hz, 1H), 3.70 (b, 1H), 3.46 (s, 2H), 2.78 (b, 2H), 2.03 – 1.95 (m, 2H), 1.75 (b, 2H), 1.55 (d, *J* = 8.7 Hz, 2H). **^{13}C NMR (151 MHz, DMSO-*d*$_6$)** δ 161.99, 161.65, 161.16, 152.92, 143.51, 139.12, 139.02, 138.08, 136.79, 133.90, 133.72, 133.51, 130.15, 129.86, 129.64, 129.38, 128.41, 128.37, 128.16, 127.46, 125.88, 120.25, 111.76, 105.90, 69.78, 59.75, 52.24, 46.68, 31.67. **HRMS (*m/z*)** calcd.: 589.2148 for $C_{34}H_{31}N_5O_3S$ [M+H]$^+$, found: 590.2224.

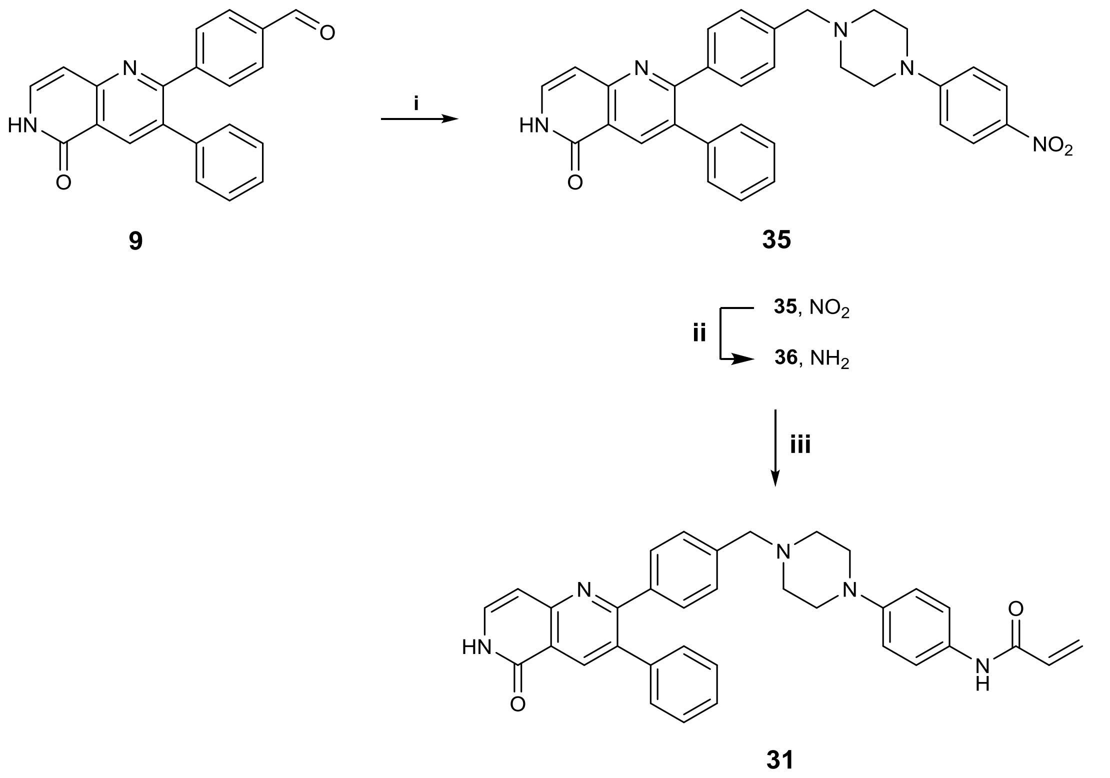

Fig S2 Synthetic scheme for the synthesis of **31**. i 1-(4-nitrophenyl)piperazine, formic acid, MeCN, 80 °C, ovn. ii Fe, NH_4Cl, MeOH/H_2O 9:1, 2 h. iii acryloyl chloride, DIPEA, THF, rt, 2 h.

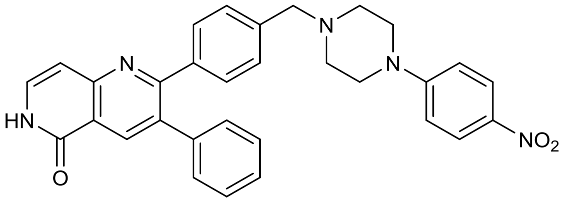

35

Synthesis of 2-(4-((4-(4-nitrophenyl)piperazin-1-yl)methyl)phenyl)-3-phenyl-1,6-naphthyridin-5(6H)-one (**35**)**.** 4-(5-oxo-3-phenyl-5,6-dihydro-1,6-naphthyridin-2-yl)-benzaldehyde (**9**, 100 mg, 0.3 mmol) and 1-(4-nitrophenyl)piperazine (1.5 eq., 96 mg, 0.4 mmol) were used following Common Procedure E and the crude product were used without further purification. **LCMS (*m/z*)** calcd.: 518.21 for $C_{32}H_{29}N_4O_3$ $[M+H]^+$, found: 518.12.

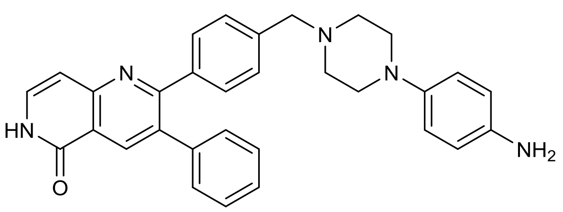

36

Synthesis of 2-(4-((4-(4-aminophenyl)piperazin-1-yl)methyl)phenyl)-3-phenyl-1,6-naphthyridin-5(6H)-one (**36**). 2-(4-((4-(4-nitrophenyl)piperazin-1-yl)methyl)phenyl)-3-phenyl-1,6-naphthyridin-5(6*H*)-one (**35**, 147 mg, 0.3 mmol) was used following Common Procedure D and yielded the title product as a yellow solid (60 mg, 0.1 mmol, 40 % over two steps). ^{1}H NMR (500 MHz, MeOD-d_4): ▯ 8.64 (s, 1H), 7.39 (dd, *J* = 9.6, 7.8 Hz, 4H), 7.28 (dt, *J* = 12.8, 8.5 Hz, 5H), 7.21 (dd, *J* = 6.4, 2.8 Hz, 2H), 6.89 (d, *J* = 7.4 Hz, 1H), 6.84 (d, *J* = 7.9 Hz, 2H), 6.72 (d, *J* = 7.9 Hz, 2H), 4.64 (s, 2H), 3.60 (s, 2H), 3.06 (s, 4H), 2.64 (s, 4H). **^{13}C NMR (126 MHz, MeOD-d_4)** δ 163.90, 162.85, 153.52, 139.12, 138.94, 138.07, 137.96, 135.58, 132.45, 130.24, 129.89, 129.56, 128.63, 127.87, 121.15, 119.16, 117.12, 107.66, 62.75, 53.23, 51.12. **HRMS (*m/z*)** calcd. for $C_{31}H_{30}N_5O$ $[M+H]^+$, 488.2444; found: 488.2446.

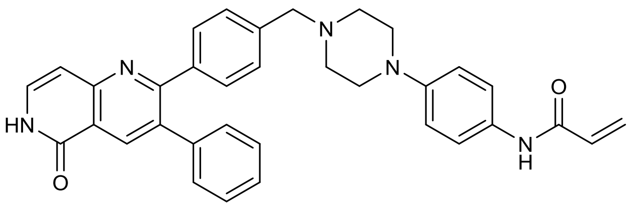

31

Synthesis of N-(4-(4-(4-(5-oxo-3-phenyl-5,6-dihydro-1,6-naphthyridin-2-yl)benzyl)-piperazin-1-yl)phenyl)acrylamide (**31**). 2-(4-((4-(4-aminophenyl)piperazin-1-yl)methyl)phenyl)-3-phenyl-1,6-naphthyridin-5(6H)-one (**36**, 45 mg, 0.1 mmol) was used following Common Procedure G and yielded the title product as a white solid (21 mg, 0.04 mmol, 42 %). **^{1}H NMR (500 MHz, MeOD-d_4)** δ 8.62 (s, 1H), 7.72 (s, 1H), 7.49 (d, *J* = 9.0 Hz, 2H), 7.42 (d, *J* = 7.4 Hz, 1H), 7.37 (d, *J* = 8.1 Hz, 2H), 7.32 – 7.24 (m, 5H), 7.23 – 7.18 (m, 2H), 6.92 (d, *J* = 9.1 Hz, 2H), 6.86 (d, *J* = 7.2 Hz, 1H), 6.37 (dd, *J* = 17.0, 9.7 Hz, 1H), 6.31 (dd, *J* = 16.9, 2.1 Hz, 1H), 5.70 (dd, *J* = 9.7, 2.1 Hz, 1H), 3.59 (s, 2H), 3.16 (s, 4H), 2.62 (s, 4H). **^{13}C NMR (126 MHz, MeOD-d_4, $CDCl_3$)** δ 164.95, 163.92, 153.54, 140.32, 139.17, 138.94, 137.96, 135.60,

132.64, 132.59, 131.56, 130.19, 129.86, 129.47, 128.59, 127.84, 127.09, 126.49, 121.67, 121.10, 116.89, 107.45, 62.66, 53.09, 49.58. **HRMS (*m/z*)** calcd. for $C_{34}H_{32}N_5O_2$ $[M+H]^+$, 542.2550; found: 542.2551.

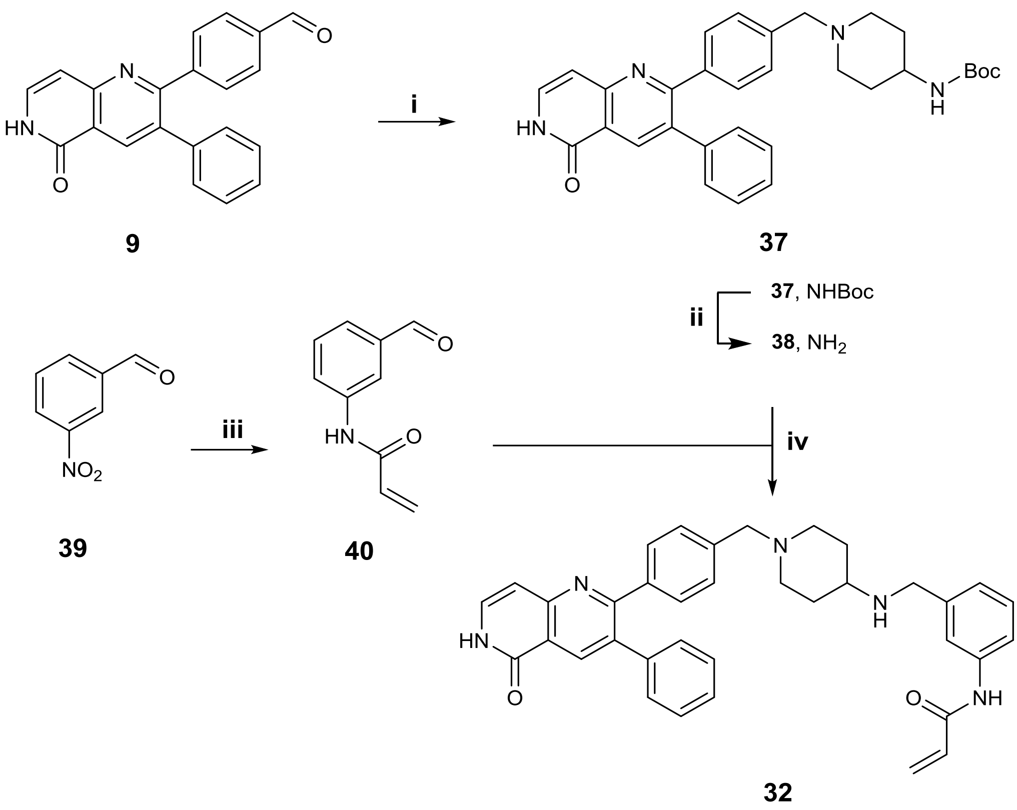

Fig S3 Synthetic scheme for the synthesis of **32**. i 4-N-Boc-aminopiperidine, formic acid, MeCN, 80 °C, ovn. ii 4 N HCl/1,4-dioxane, rt, 1 h. iii $SnCl_2$, EtOH, reflux, 1 h, then acryloyl chloride, DIPEA, DCM, rt, 2 h. iv formic acid, MeCN, 80 °C, ovn.

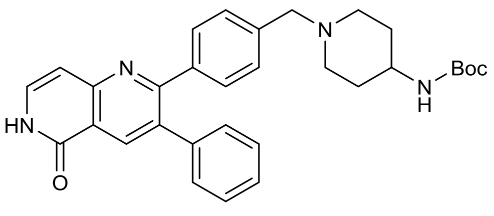

37

Synthesis of tert-butyl (1-(4-(5-oxo-3-phenyl-5,6-dihydro-1,6-naphthyridin-2-yl)benzyl)piperidin-4-yl)carbamate (**37**). 4-(5-oxo-3-phenyl-5,6-dihydro-1,6-naphthyridin-2-yl)benzaldehyde (**9**, 413 mg, 1.3 mmol) and 4-*N*-Boc-aminopiperidine (1.3 eq., 329 mg, 1.6 mmol) were used following Common Procedure E and yielded the title product as a pale yellow solid (277 mg, 0.5 mmol, 43 %). **^{1}H NMR (500 MHz, DMSO-*d*$_6$)** δ 11.62 (d, *J* = 5.6 Hz, 1H), 8.39 (s, 1H), 7.50 (dt, *J* = 14.3, 7.2 Hz, 1H), 7.37 – 7.29 (m, 5H), 7.29 – 7.20 (m, 4H), 6.68 (d, *J* = 7.3 Hz, 2H), 3.65 (b, 1H), 3.39 – 3.26 (m, 4H), 2.85 (b, 2H), 1.73 – 1.69 (m, 2H), 1.44 (d, *J* = 9.9 Hz, 2H), 1.37 (s, 9H). **^{13}C NMR (151 MHz, DMSO-*d*$_6$)** δ 163.67, 162.46, 161.64, 155.29, 153.37, 139.47, 138.62, 137.26, 134.16, 133.98, 130.10, 129.84, 128.88, 128.72, 127.92, 120.70, 106.37, 77.90, 62.04, 52.51, 47.84, 32.18, 28.73. **LCMS (*m/z*)** calcd. for $C_{31}H_{34}N_4O_3$ $[M+H]^+$, 511.26; found, 511.17.

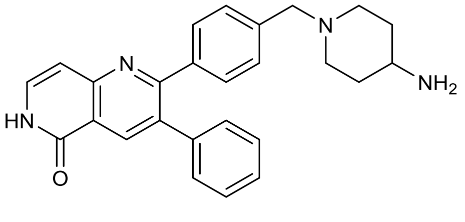

38

Synthesis of 2-(4-((4-aminopiperidin-1-yl)methyl)phenyl)-3-phenyl-1,6-naphthyridin-5(6H)-one (**38**). *tert*-butyl (1-(4-(5-oxo-3-phenyl-5,6-dihydro-1,6-naphthyridin-2-yl)benzyl)piperidin-4-yl)carbamate (**37**, 313 mg, 0.6 mmol) was used following Common Procedure F and yielded the title product as a yellow solid. **^{1}H NMR (700 MHz, DMSO-*d*$_6$)** δ 11.75 (s, 1H), 8.44 (s, 1H), 7.57 (d, *J* = 8.1 Hz, 2H), 7.55 – 7.53 (m, 1H), 7.42 (t, *J* = 7.6 Hz, 2H), 7.35 – 7.32 (m, 3H), 7.26 – 7.23 (m, 2H), 6.75 – 6.71 (m, 1H), 6.13 (b, 2H), 4.25 (d, *J* = 4.8 Hz, 2H), 3.31 (d, *J* = 11.7 Hz, 2H), 3.26 – 3.20 (m, 1H), 3.00 (dd, *J* = 22.8, 10.7 Hz, 2H), 2.12 (d, *J* = 11.9 Hz, 2H), 2.05 – 1.97 (m, 2H).**^{13}C NMR (176 MHz, DMSO-*d*$_6$)** δ 161.85, 160.32,

152.58, 140.06, 138.54, 137.59, 134.30, 134.00, 131.12, 130.62, 130.36, 130.17, 130.13, 129.59, 128.62, 127.79, 120.76, 105.28, 66.47, 49.55, 45.38, 26.83. **LCMS (*m/z*)** calcd. for $C_{26}H_{26}N_4O$ [M+H]$^+$, 511.21; found, 411.20.

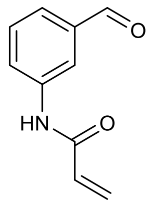

40

Synthesis of N-(3-formylphenyl)acrylamide (**40**)**.** 3-nitrobenzaldehyde (**39**, 300 mg, 2.0 mmol) was dissolved in 20 mL MeOH and tin chloride (4 eq., 1.5 g, 7.9 mmol) was added. The reaction mixture was allowed to stir for 1 h at reflux temperature. The suspension was basified with sat. $NaHCO_3$ solution, the precipitate was filtered off and the product was extracted from the filtrate with dichloromethane. The combined organic fractions were concentrated *in vacuo* to a volume of 30 mL, then DIPEA (3 eq., 1.0 mL, 6.0 mmol) and acryloyl chloride (1.2 eq., 178 µL, 2.3 mmol) were added and the solution was allowed to stir for 2 h at rt. The reaction mixture was washed with saturated NH_4Cl solution and extracted with DCM. The combined organic fractions were evaporated and silica column chromatography (1-10 % MeOH/DCM + 1 % NH_3) yielded the desired product as a yellow oil (302 mg, 1.7 mmol, 87 %). **^{1}H NMR (500 MHz, DMSO-*d*$_6$)** δ 10.44 (s, 1H), 9.98 (s, 1H), 8.26 (s, 1H), 7.92 (d, *J* = 8.0 Hz, 1H), 7.64 (d, *J* = 7.6 Hz, 1H), 7.57 (t, *J* = 7.8 Hz, 1H), 6.45 (dd, *J* = 17.0, 10.1 Hz, 1H), 6.30 (dd, *J* = 17.0, 1.7 Hz, 1H), 5.80 (dd, *J* = 10.1, 1.7 Hz, 1H). **^{13}C NMR (126 MHz, DMSO-*d*$_6$)** δ 193.11, 163.50, 139.83, 136.79, 131.58, 129.80, 127.60, 125.27, 125.07, 119.11. **LC-MS (ESI-MS)** calcd. for $C_{10}H_9NO_2$ [M+H]$^+$, 176.06; found, 176.02.

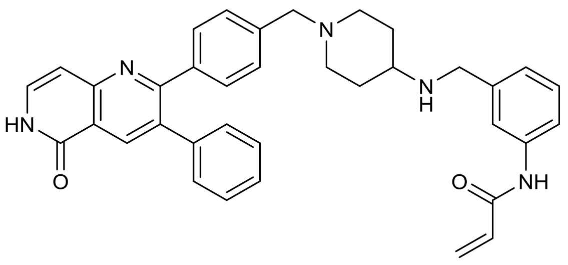

32

Synthesis of N-(3-(((1-(4-(5-oxo-3-phenyl-5,6-dihydro-1,6-naphthyridin-2-yl)benzyl)-piperidin-4-yl)-amino)methyl)phenyl)acrylamide (**32**). 2-(4-((4-aminopiperidin-1-yl)methyl)phenyl)-3-phenyl-1,6-naphthyridin-5(6*H*)-one (38, 200 mg, 0.5 mmol) and *N*-(3-formylphenyl)acrylamide (**39**, 1.1 eq., 97 mg, 0.5 mmol) were used following Common Procedure E and yielded the title product as a white solid (37 mg, 0.06 mmol, 13 %). **^{1}H NMR (400 MHz, DMSO-*d*$_6$)** δ 11.60 (b, 1H), 10.10 (s, 1H), 8.38 (s, 1H), 7.61 – 7.56 (m, 2H), 7.49 (d, *J* = 7.3 Hz, 1H), 7.33 – 7.27 (m, 5H), 7.26 – 7.16 (m, 6H), 7.02 (d, *J* = 7.5 Hz, 1H), 6.68 (d, *J* = 7.3 Hz, 1H), 6.44 (dd, *J* = 17.0, 10.1 Hz, 1H), 6.24 (dd, *J* = 17.0, 1.8 Hz, 1H), 5.73 (dd, *J* = 10.1, 1.8 Hz, 1H), 3.70 (s, 2H), 3.29 (b, 2H), 2.71 (d, *J* = 11.1 Hz, 2H), 2.38 (t, *J* = 9.9 Hz, 1H), 1.90 (t, *J* = 10.7 Hz, 2H), 1.79 (d, *J* = 10.6 Hz, 2H), 1.28 (dd, *J* = 20.5, 10.2 Hz, 2H). **^{13}C NMR (101 MHz, DMSO-*d*$_6$)** δ 163.04, 161.99, 161.20, 152.91, 141.85, 139.11, 139.03, 138.87, 138.00, 136.77, 133.69, 133.48, 131.96, 129.57, 129.36, 128.39, 128.14, 127.43, 126.68, 123.08, 120.22, 118.82, 117.58, 105.90, 61.77, 53.75, 51.85, 49.82, 32.02. **HRMS (*m/z*)** calcd. for $C_{36}H_{35}N_5O_2$ [M+H]$^+$, 570.2864; found: 570.2861.

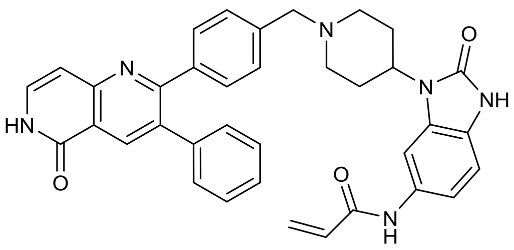

1

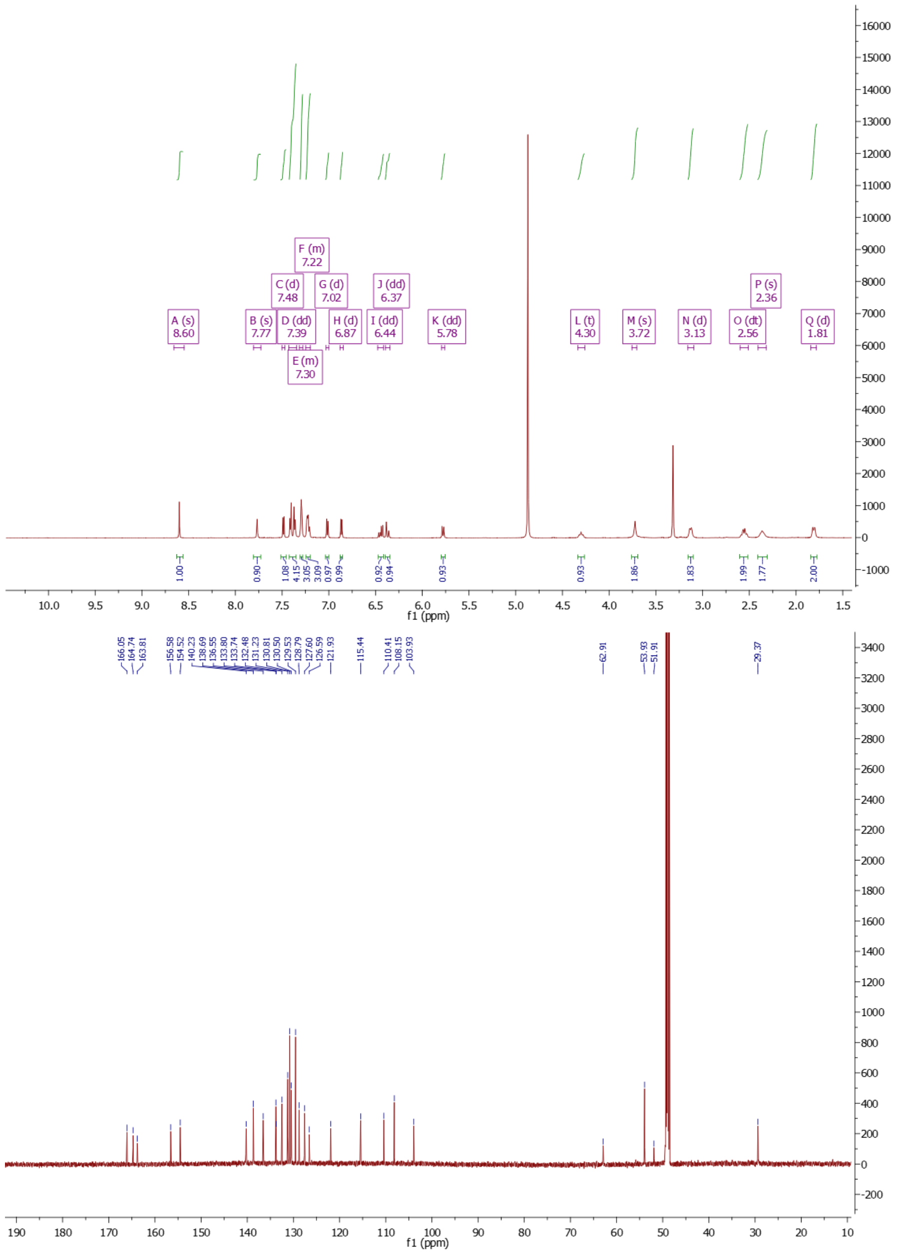

Fig S4. ^{1}H and ^{13}C-NMR spectra of borussertib (**1**).

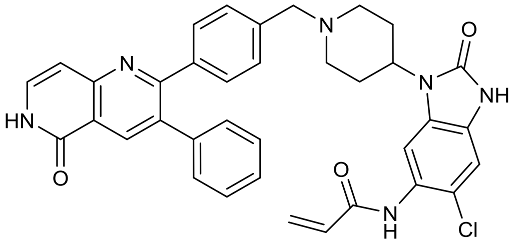

24a

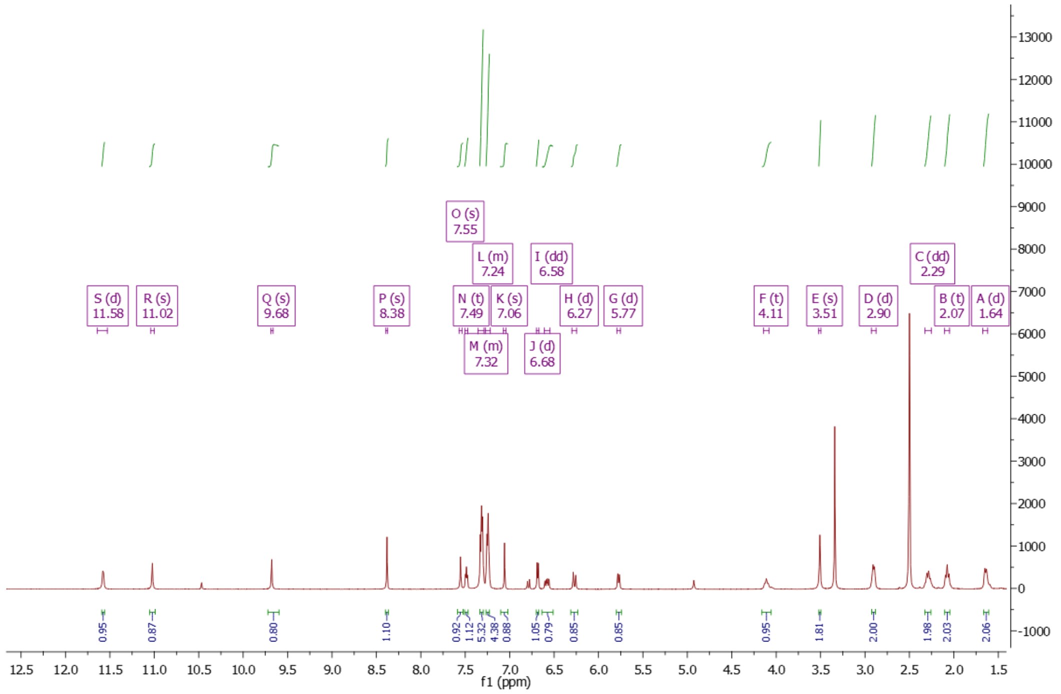

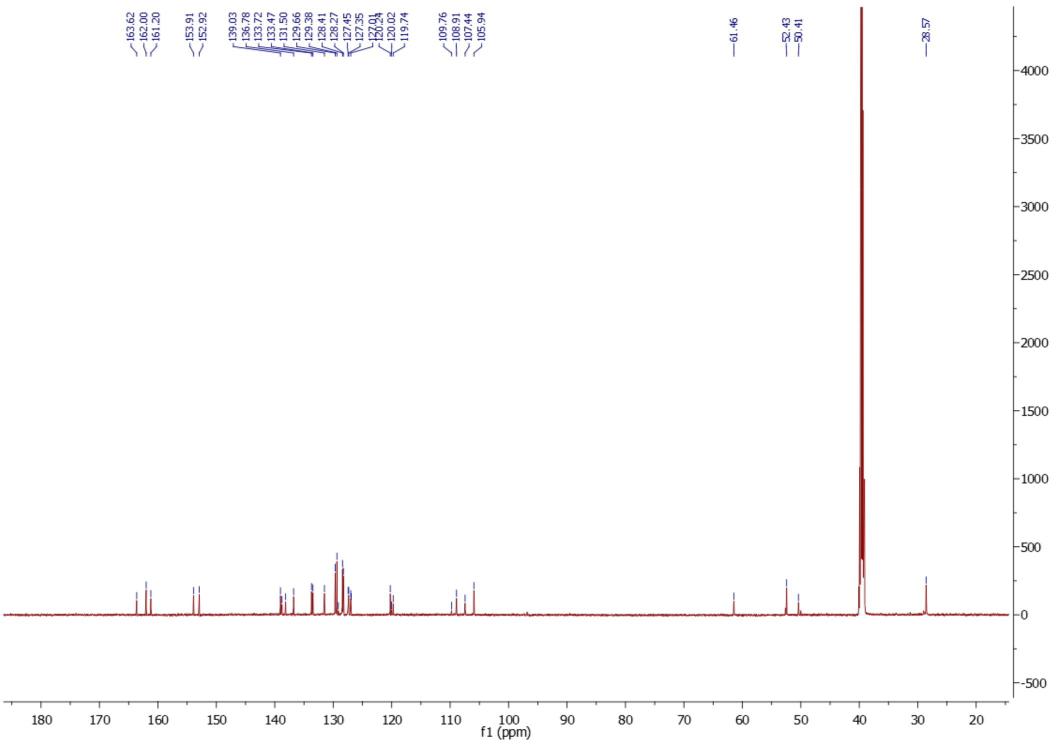

Fig S5. ^{1}H- and ^{13}C-NMR spectra of **24a**.

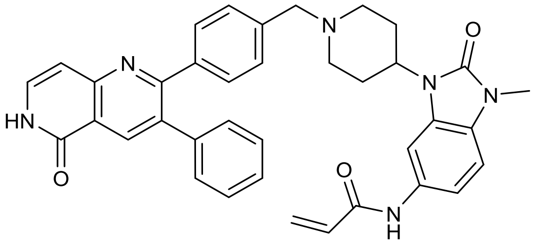

24b

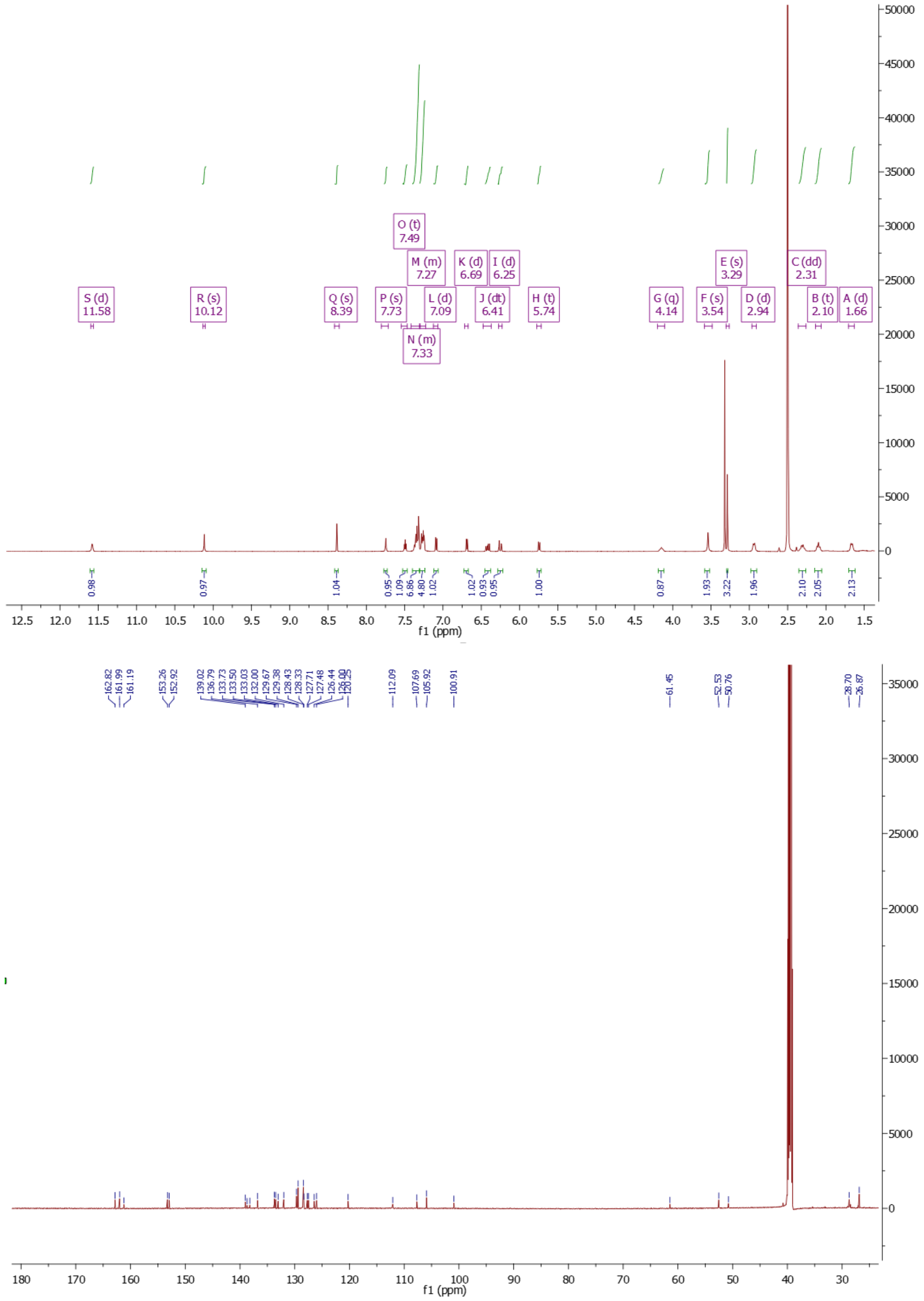

Fig S6. ^{1}H- and ^{13}C-NMR spectra of **24b**.

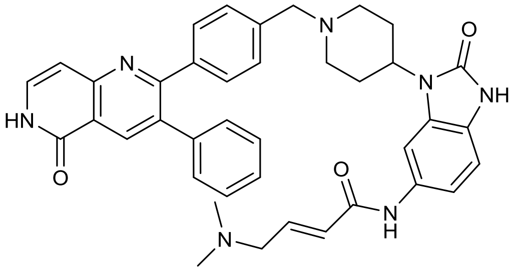

24c

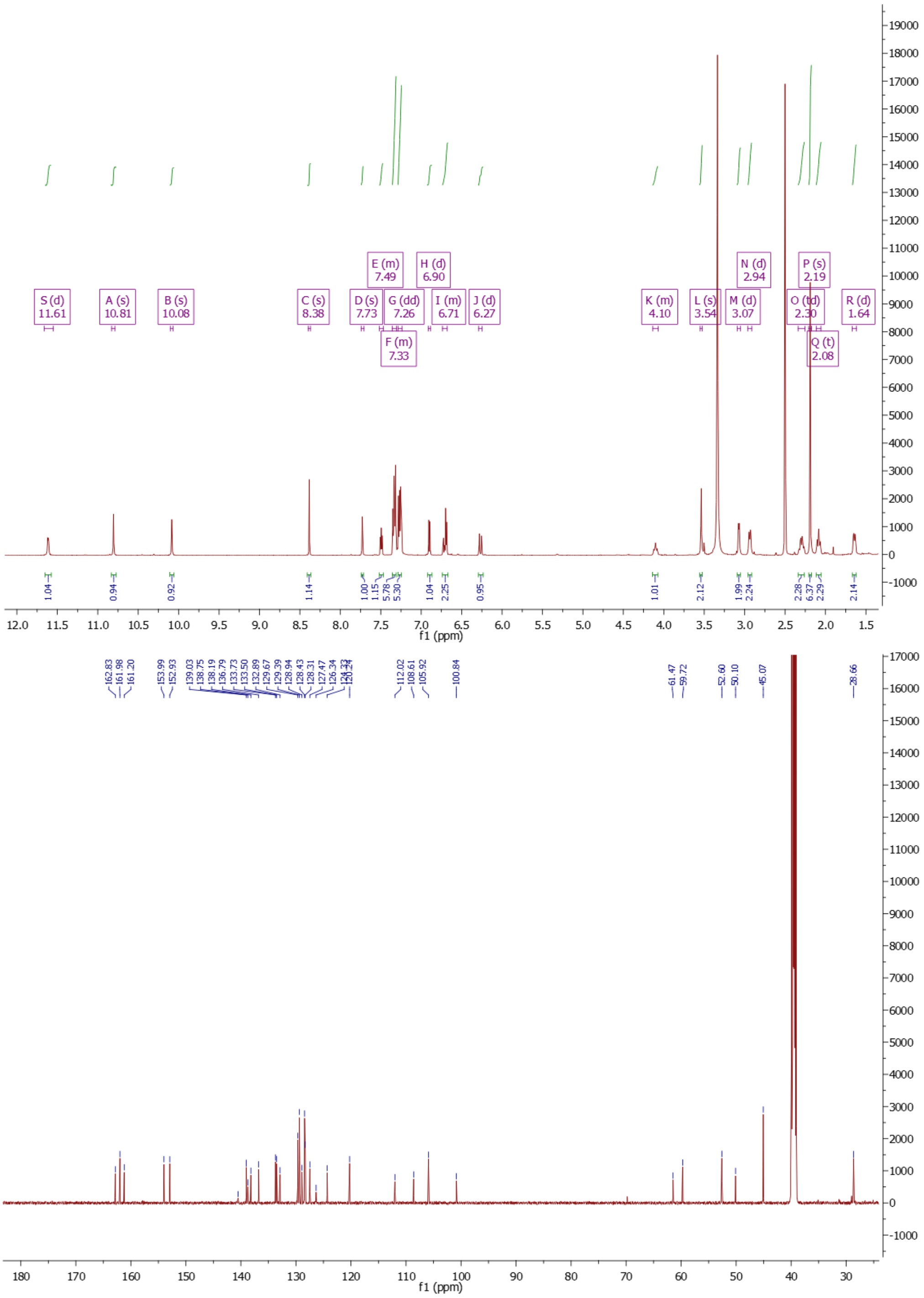

Fig S7 ^{1}H- and ^{13}C-NMR spectra of **24c**.

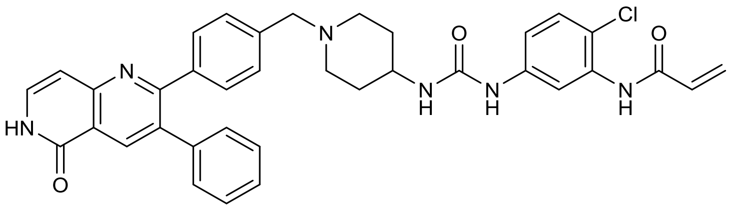

27

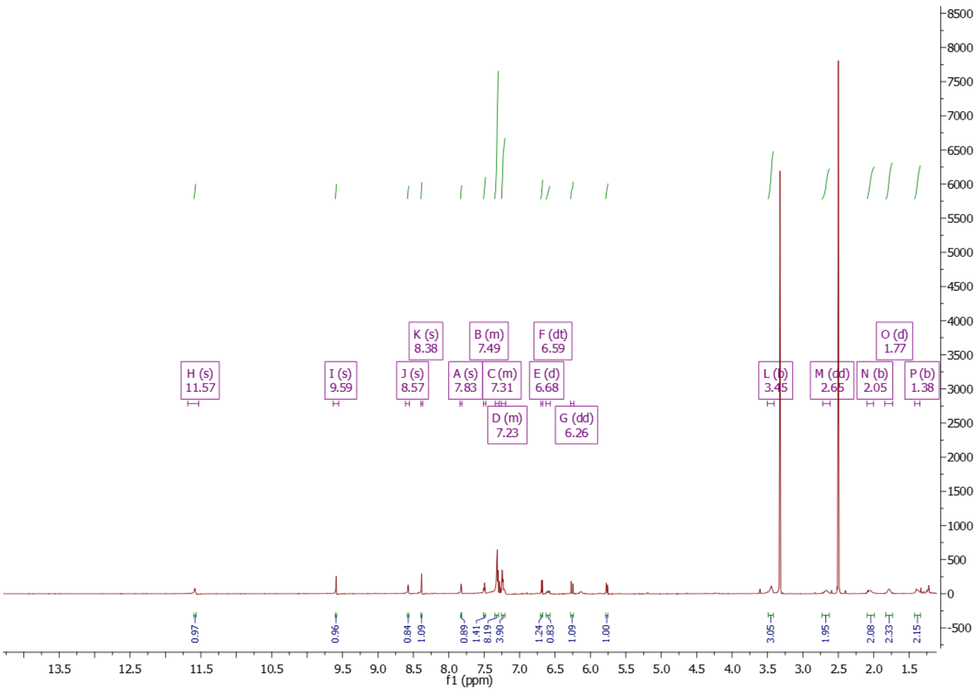

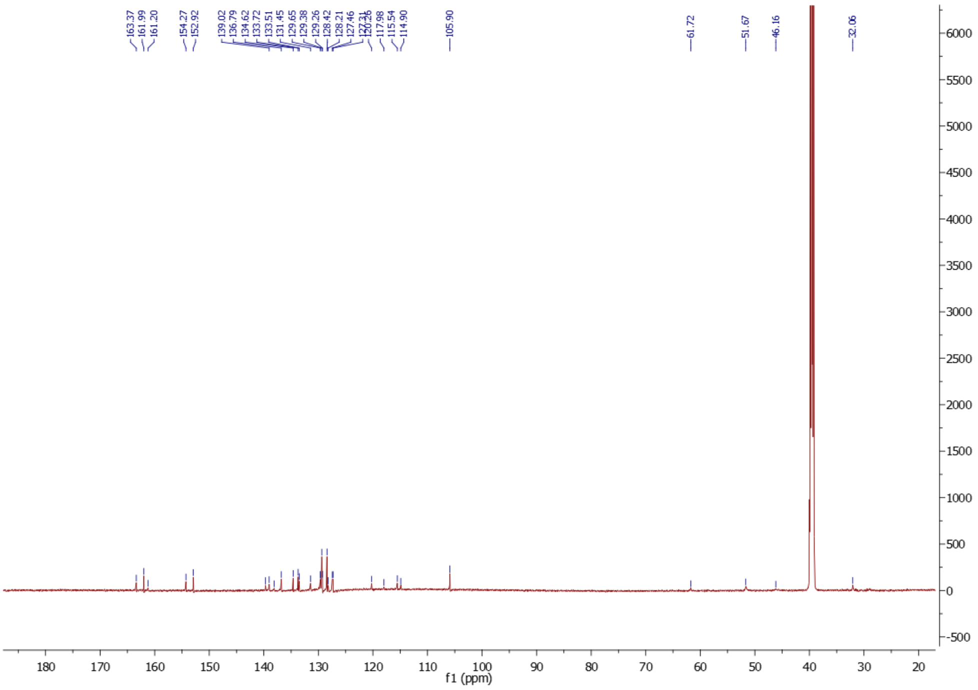

Fig S8 ^{1}H- and ^{13}C-NMR spectra of **27**.

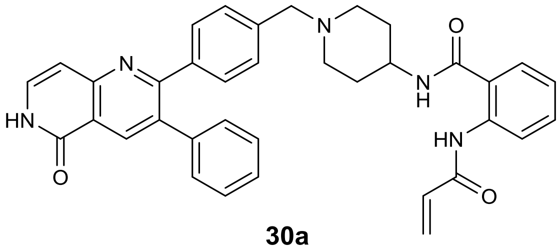

30a

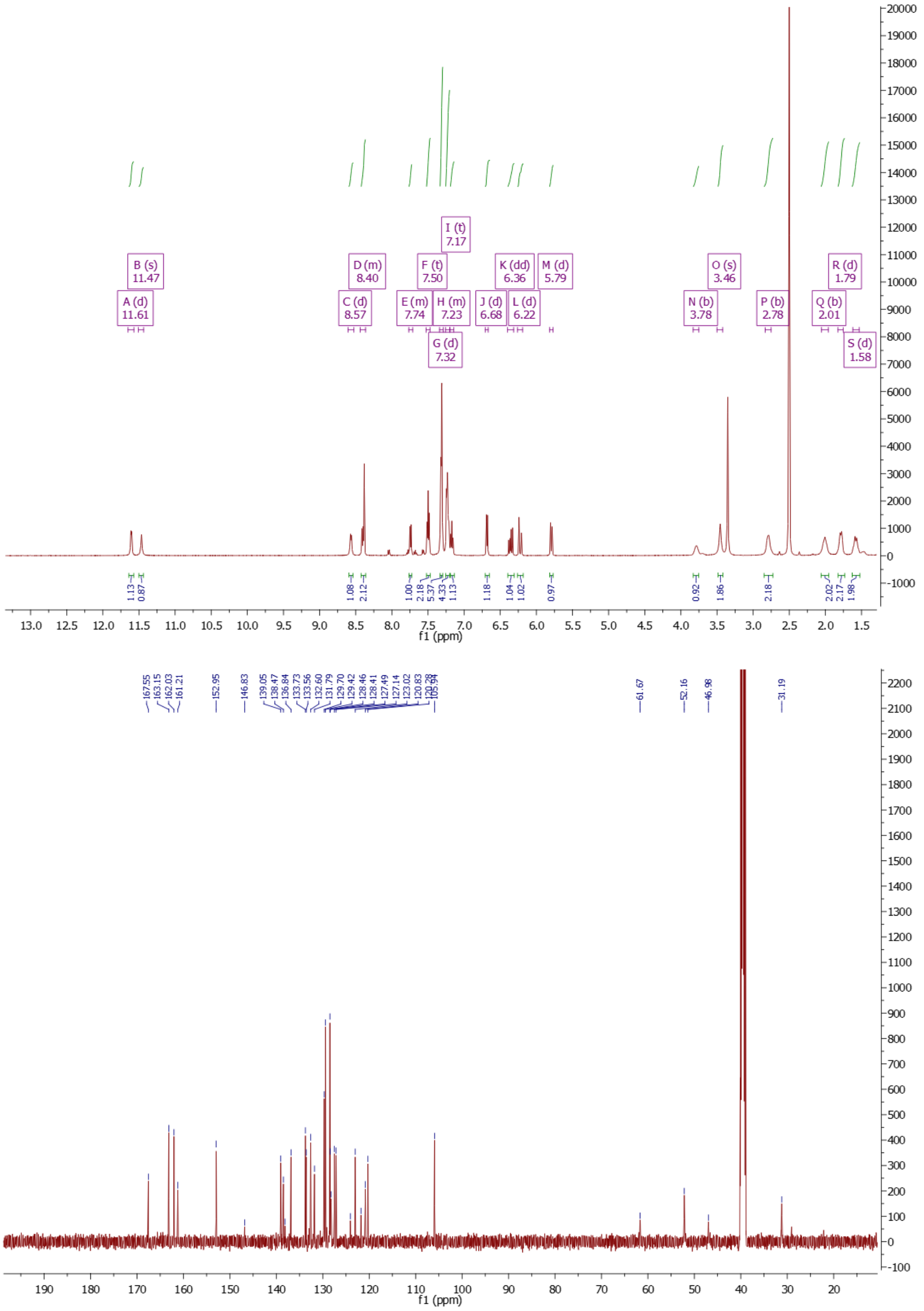

Fig S9 ^{1}H- and ^{13}C-NMR spectra of **30a**.

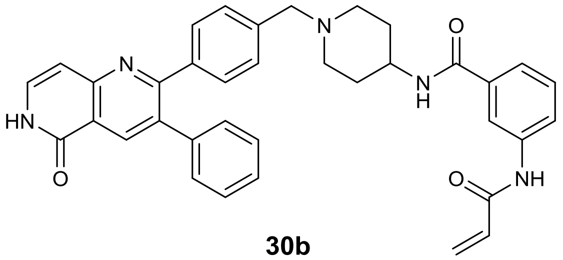

30b

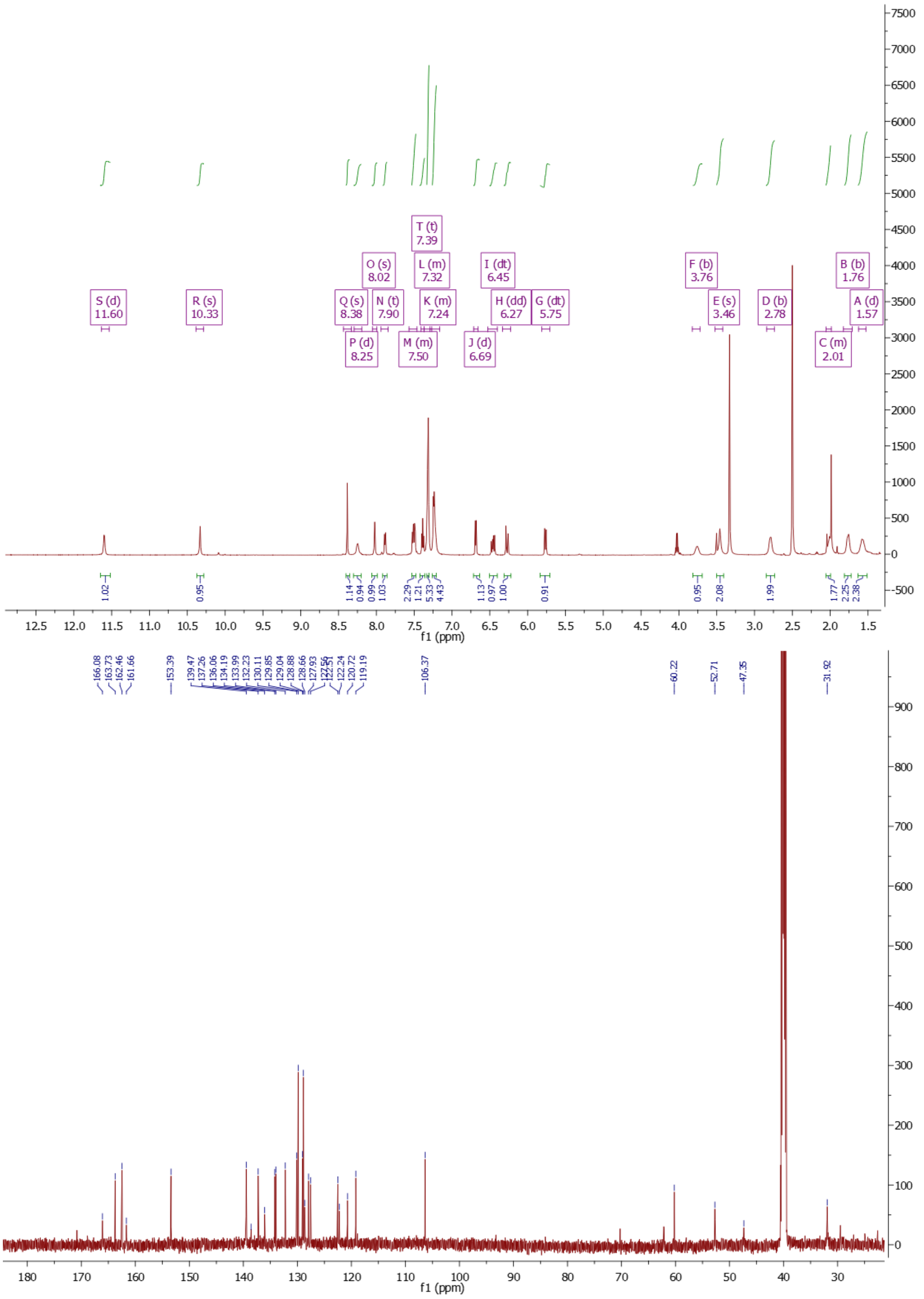

Fig S10 ^{1}H- and ^{13}C-NMR spectra of **30b**.

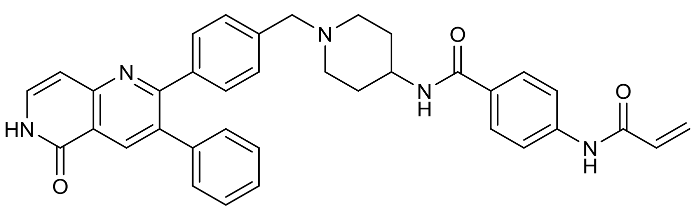

30c

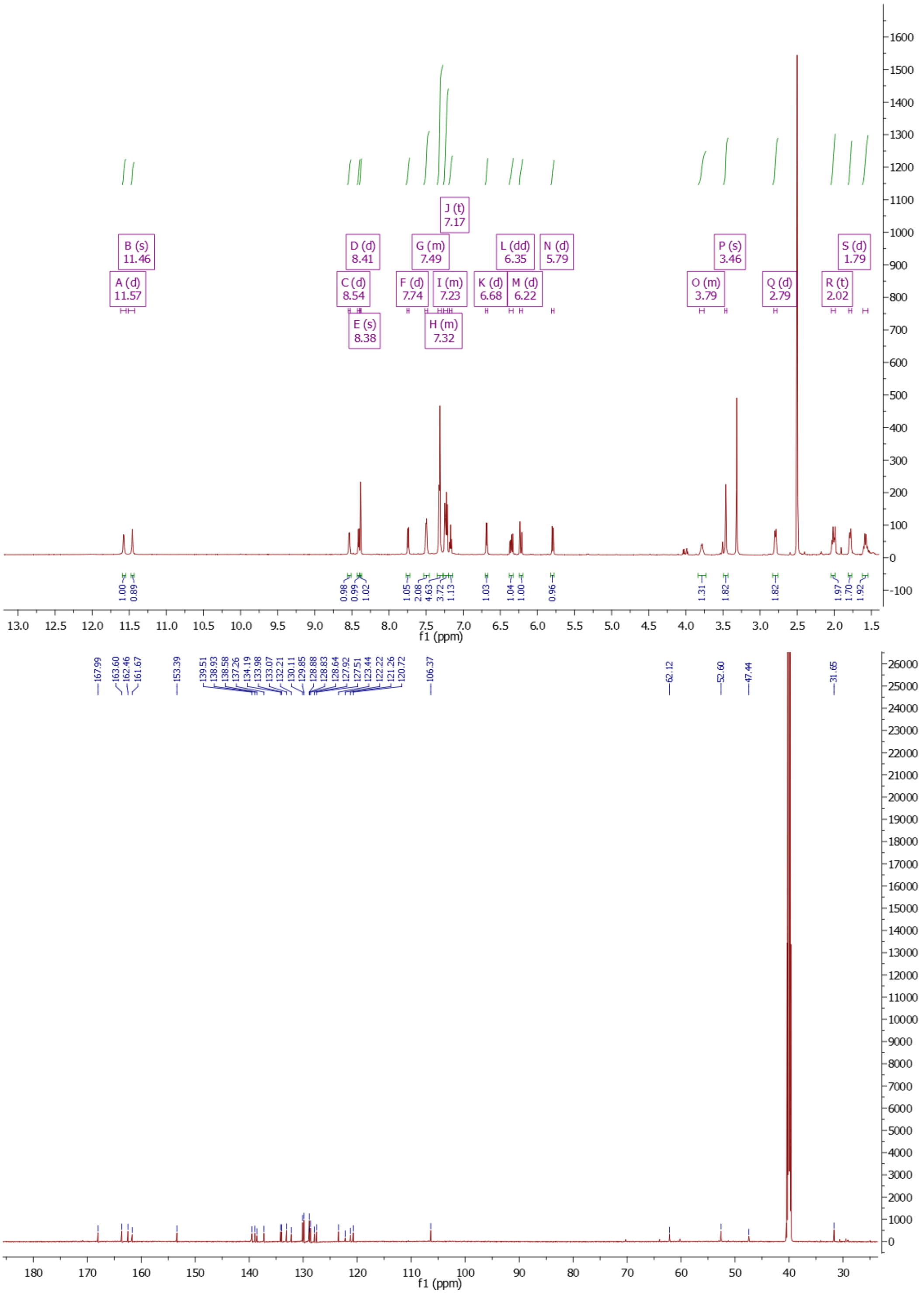

Fig S11 ^{1}H- and ^{13}C-NMR spectra of **30c**.

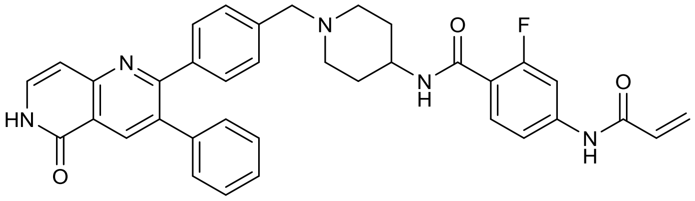

30d

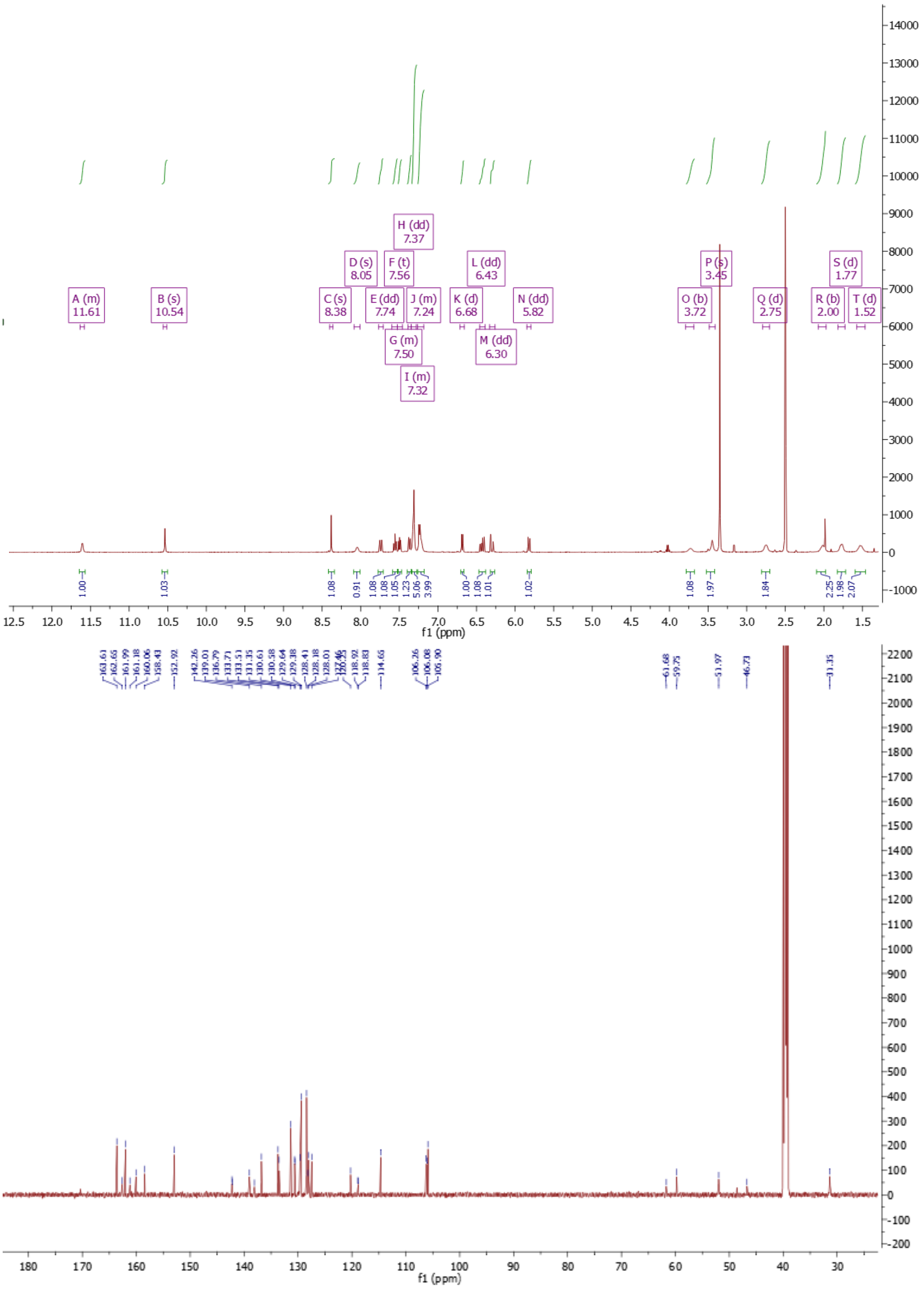

Fig S12 ^{1}H- and ^{13}C-NMR spectra of **30d**.

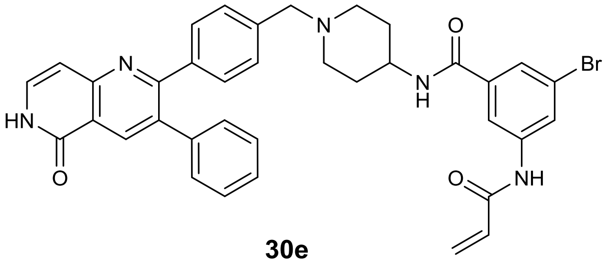

30e

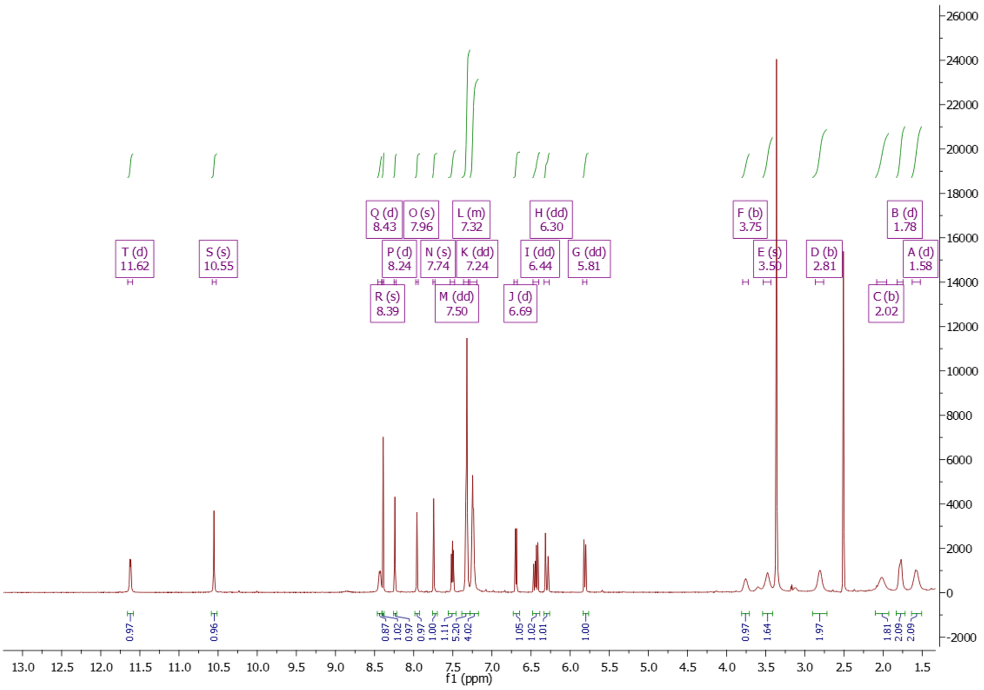

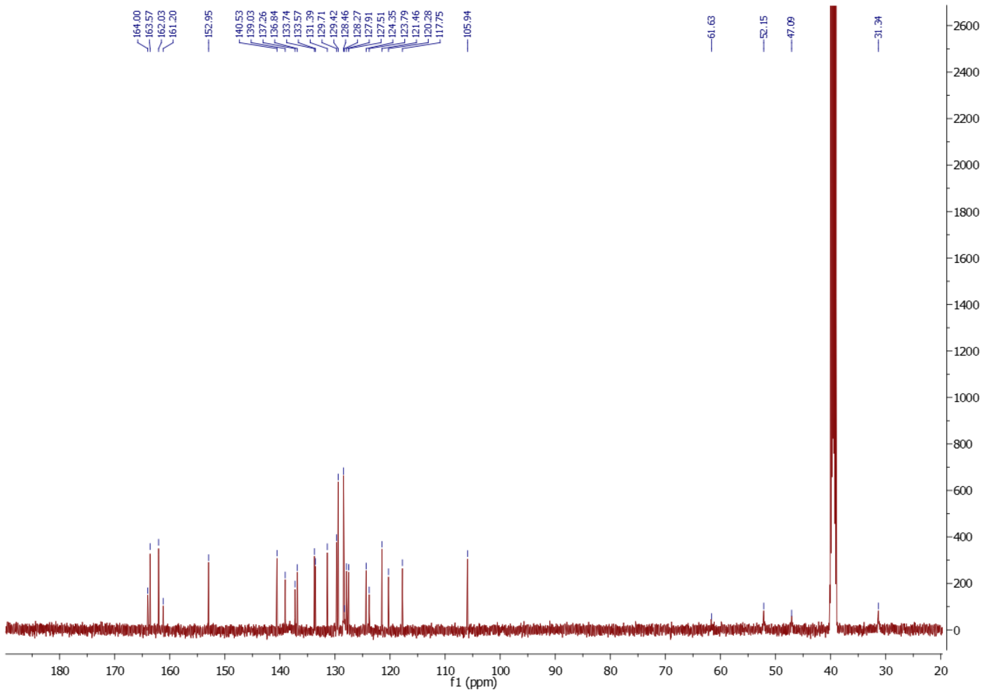

Fig S13 ^{1}H- and ^{13}C-NMR spectra of **30e**.

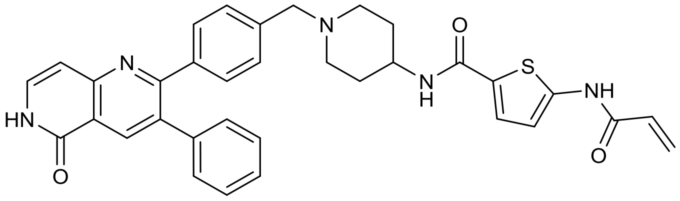

30f

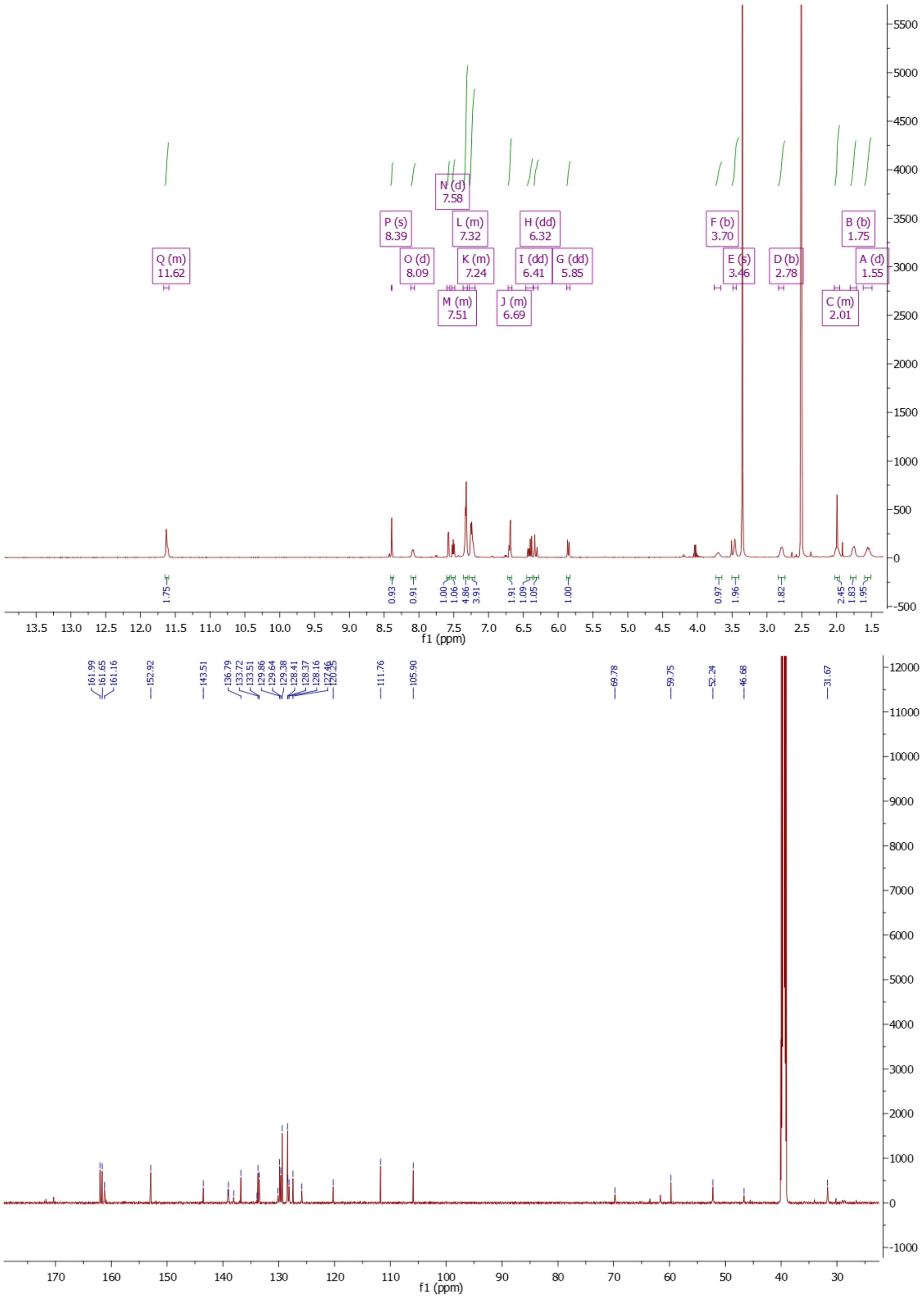

Fig S14 ^{1}H- and ^{13}C-NMR spectra of **30f**.

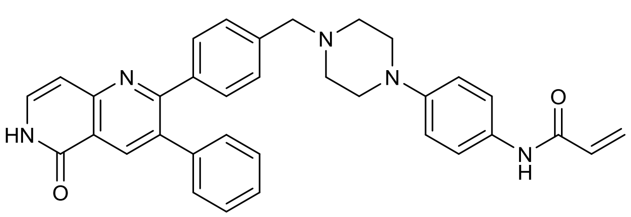

31

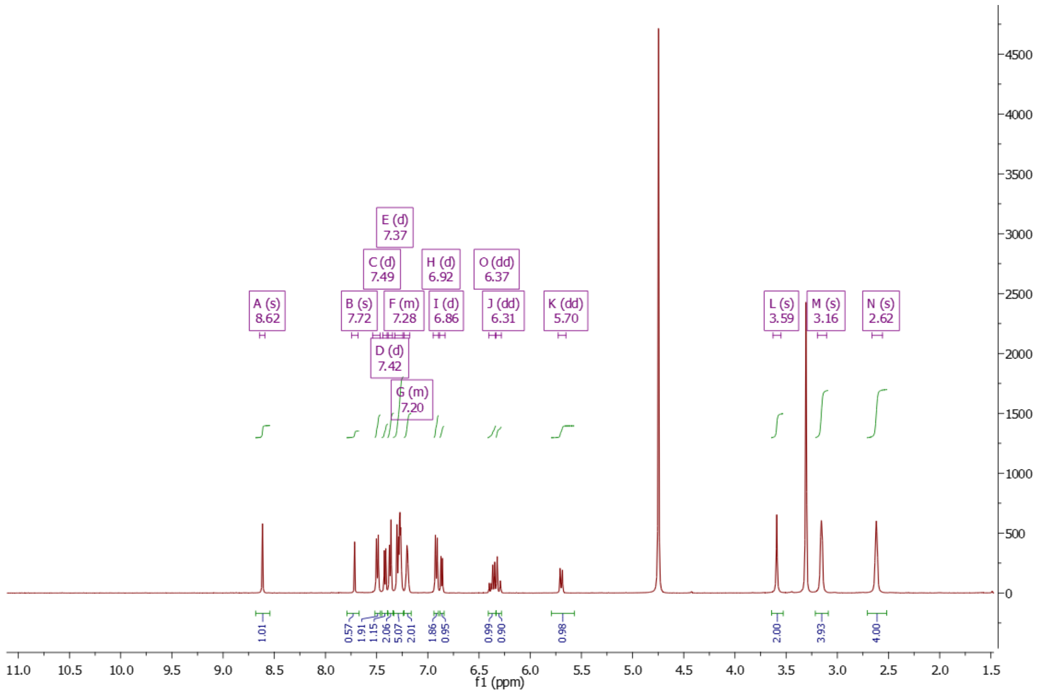

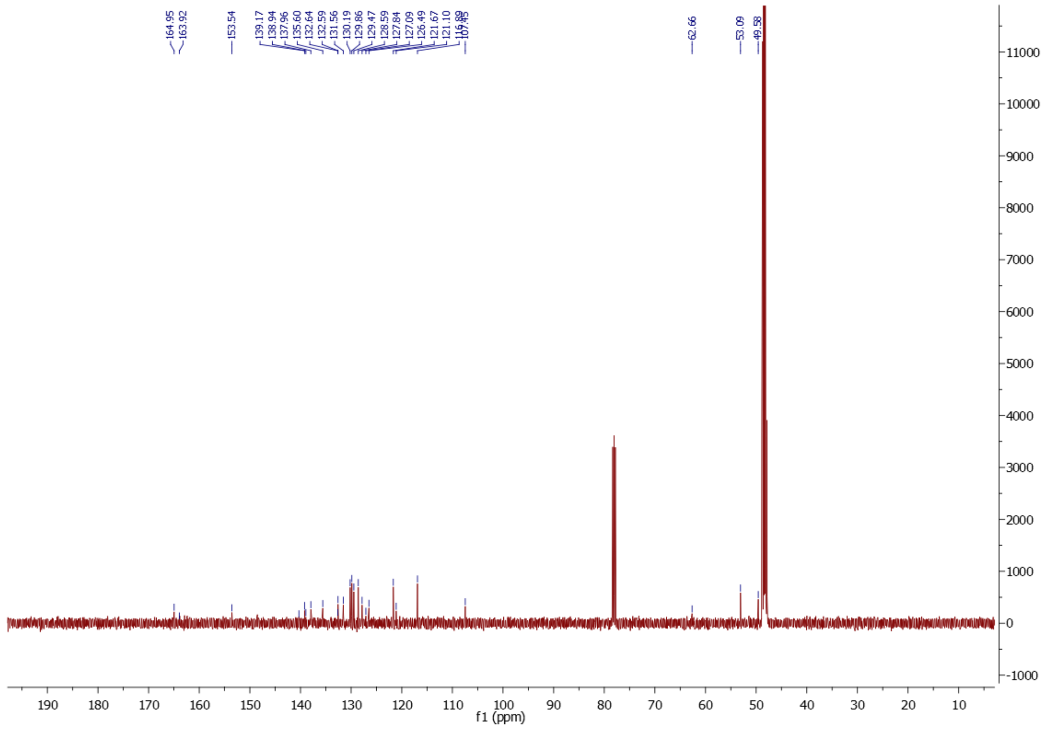

Fig S15 ^{1}H- and ^{13}C-NMR spectra of **31**.

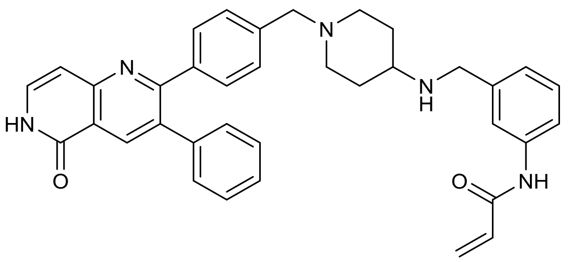

32

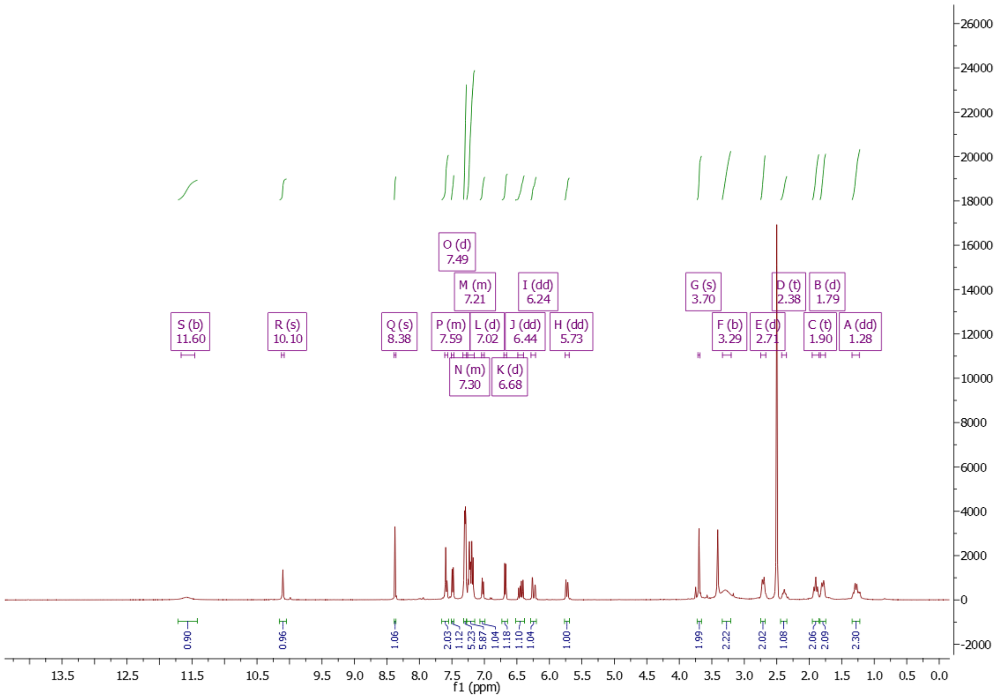

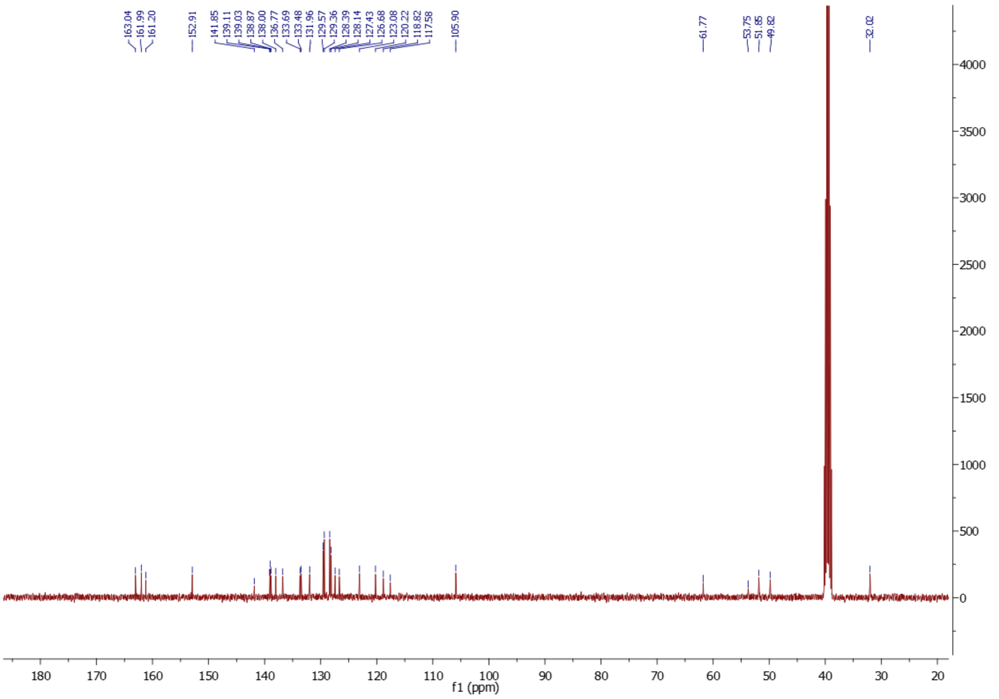

Fig S16 ^{1}H- and ^{13}C-NMR spectra of **32**.

Table S1. Data collection and refinement statistics for full-length Akt1 in complex with different covalent-allosteric Akt inhibitors.

Data collection	Akt1with 24b<br>PDB: 6HHJ	Akt1with 27<br>PDB: 6HHG	Akt1with 30b<br>PDB: 6HHI	Akt1with 31<br>PDB: 6HHH
Space group	$P2_12_12_1$	$P2_12_12_1$	$P2_12_12_1$	$P2_12_12_1$
Cell dimensions				
a, b, c (Å)	70.05, 71.37, 90.96	70.82, 71.23, 91.24	70.17, 70.96, 91.11	70.32, 70.89, 91.08
α, β, γ (°)	90, 90, 90	90, 90, 90	90, 90, 90	90, 90, 90
Resolution (Å)	43.81 - 2.30<br>(2.40 - 2.30)	45.62 - 2.30<br>(2.40 - 2.30)	45.56 - 2.70<br>(2.80 - 2.70)	45.54 - 2.70<br>(2.80 - 2.70)
R_{meas} (%)	20.1 (110.3)	12.8 (145.8)	8.5 (67.6)	7.5 (99.7)
R_{merge} (%)	18.5 (107.5)	12.6 (137.7)	8.1 (69.0)	7.4 (99.7)
I/σI	7.81 (1.37)	12.86 (1.37)	21.39 (3.71)	24.48 (2.77)
$CC_{1/2}$	99.4 (70.8)	99.8 (59.9)	99.9 (95.0)	100.0 (92.1)
Completeness (%)	99.9 (100)	99.9 (99.5)	100 (100)	100 (99.8)
Redundancy	7.2 (7.1)	12.0 (7.7)	13.1 (13.7)	13.0 (13.8)
**Refinement**				
Resolution (Å)	43.81 – 2.30	45.62 – 2.30	45.55 – 2.70	45.54 – 2.70
No. Reflections	20854	21088	12991	13003
R_{work}/R_{free}	22.20/24.35<br>(30.57.33/31.17)	21.04/24.61<br>(30.08/36.32)	22.76/25.80<br>(30.38/32.42)	21.44/26.57<br>(26.92/33.80)
No. Atoms				
Protein	3378	3190	3218	3132
Ligand/ion	46	46	44	41
Water	120	78	18	21
B-factors				
Protein	50.85	65.27	84.17	92.08
Ligand/ion	39.43	56.48	77.14	53.46
Water	41.40	57.84	64.94	69.98
rms deviations				
Bond lengths (Å)	0.002	0.002	0.003	0.005
Bond angles (°)	0.592	0.548	0.630	0.787
Wavelength (Å)	1.00000	0.99991	0.97796	0.99992
Temperature (K)	100	100	100	100
X-ray source	PX II at SLS,<br>Villingen, CH	PX II at SLS,<br>Villingen, CH	PX II at SLS,<br>Villingen, CH	PX II at SLS,<br>Villingen, CH
Detector	Pilatus 6MF	Pilatus 6MF	Pilatus 6MF	Pilatus 6MF
**Ramachandran Plot**				
Outliers (%)	0.00	0.00	0.00	0.00
Allowed (%)	2.23	2.66	2.90	3.25
Favored (%)	97.77	97.34	97.10	96.75

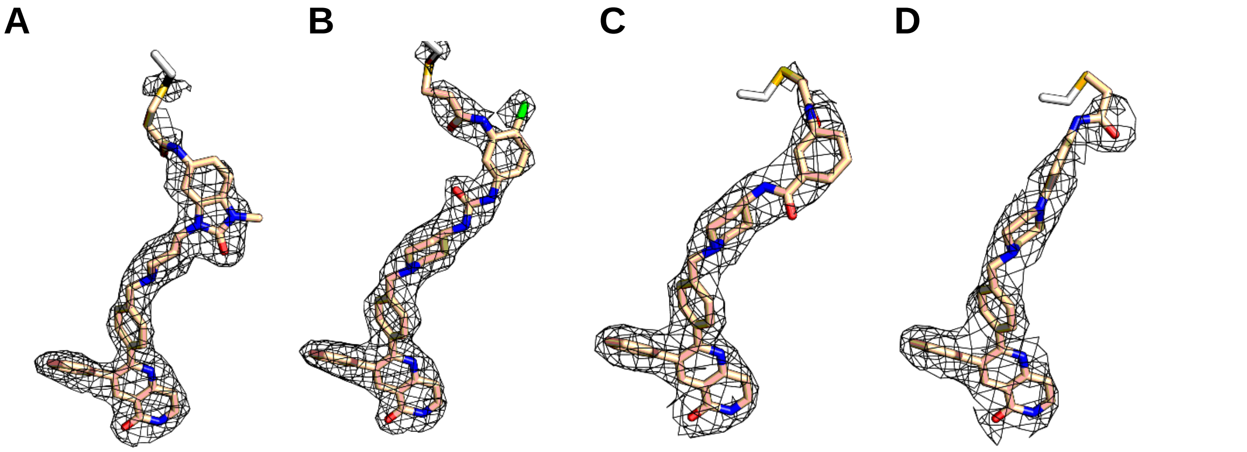

Fig. S17 Performing a simulated annealing refinement, mFo-DFc maps (2.5 *σ*) were calculated for **24b** (**A**) **27** (**B**). **30b** (**C**) and **31** (**D**).

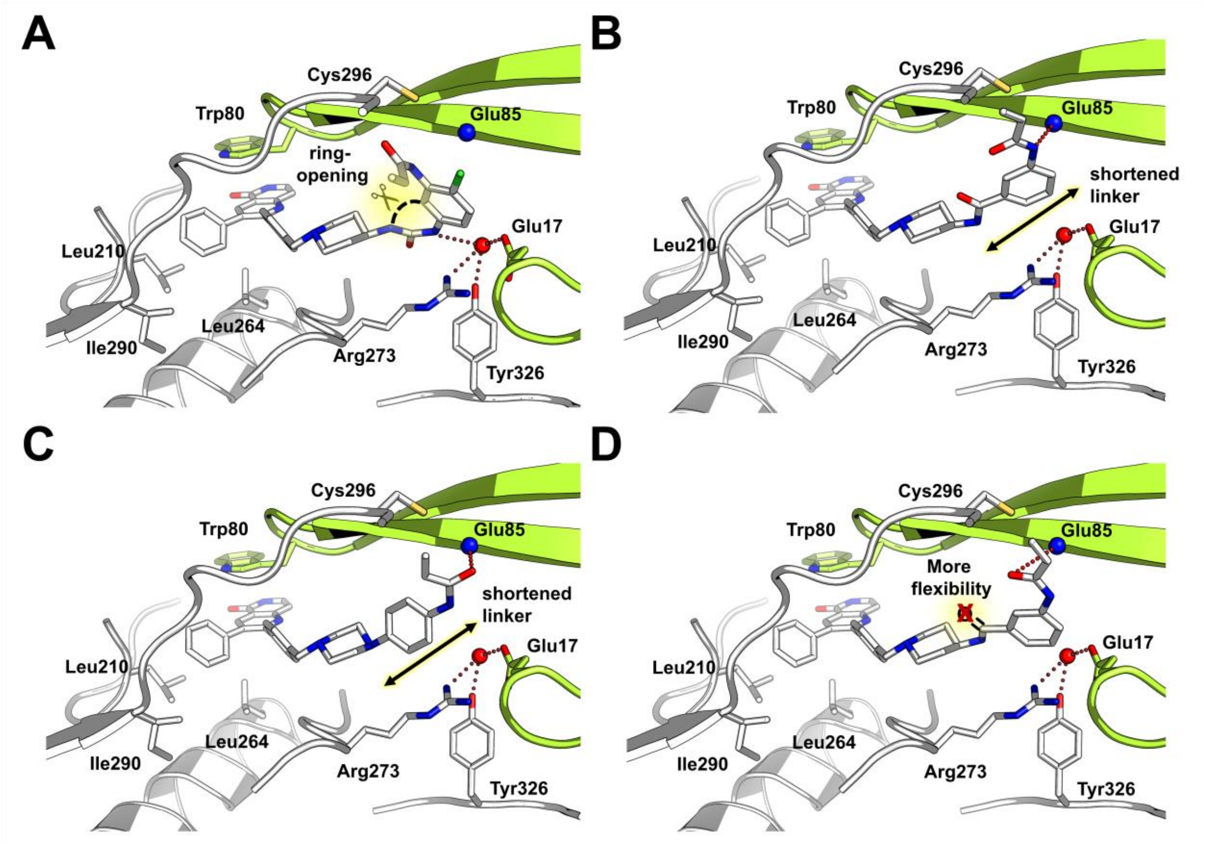

Fig S18 Novel covalent-allosteric Akt inhibitors were designed based on the insights of the structural analysis of the crystal structure of borussertib in complex with Akt (PDB: 6HHF). The rational design approach was illustrated by modeling different novel scaffolds using Accelrys Discovery Studio 3.1 **A** Ring-opening of the benzo[*d*]imidazolone moiety of borussertib results in a more flexible, elongated phenylurea-based molecule structure. The influence of molecule length and flexibility on the inhibitory properties were further investigated by shortening the linker to phenylamide (**B**) and piperazine-based (**C**) candidates and by increasing flexibility with benzylamine-based molecules (**D**).

Table S2. The covalent-allosteric Akt inhibitor **24b** was screened against a panel of 100 different protein kinases at a compound concentration of 1 µM. Measurements were performed in duplicates and at the particular apparent ATP K_M of each individual kinase employing the activity based Z'LYTE® kinase assay of the SelectScreen® Kinase Profiling Services (ThermoFisher Scientific). The data shows average % inhibition of two independent measurements of assessed kinases.

Kinase	% inhibition **24b**	Kinase	% inhibition **24b**
AKT1 (PKB alpha)	98	CDK1/cyclin B	3
AKT2 (PKB beta)	97	CDC42 BPA (MRCKA)	3
AKT3 (PKB gamma)	75	PRKD2 (PKD2)	3
MELK	48	PRKCI (PKC iota)	3
RPS6KB1 (p70S6K)	32	CDC42 BPB (MRCKB)	3
MAPKAPK2	29	MAP4K5 (KHS1)	3
BMX	28	TEK (Tie2)	2
RPS6KA2 (RSK3)	25	MARK1 (MARK)	2
PRKCA (PKC alpha)	23	JAK2	2
JAK3	23	ABL1	2
MAPKAPK3	23	MAPK1 (ERK2)	2
GSK3B (GSK3 beta)	22	BRSK1 (SAD1)	2
AMPK A2/B1/G1	20	PRKACA (PKA)	2
SRC	19	MAPK14 (p38 alpha) Direct	2
SGK (SGK1)	19	MET (cMet)	1
AMPK A1/B1/G1	17	MAPK3 (ERK1)	0
ROCK1	16	PRKCD (PKC delta)	0
RPS6KA3 (RSK2)	14	NTRK2 (TRKB)	0
RPS6KA1 (RSK1)	14	GRK6	0
PRKD1 (PKC mu)	13	AURKB (Aurora B)	0
PRKCE (PKC epsilon)	12	ERBB2 (HER2)	-1
PAK1	12	PKN1 (PRK1)	-1
PAK4	12	DCAMKL2 (DCK2)	-1
BTK	12	ACVR1B (ALK4)	-1
RET	10	SGK2	-1
PRKCN (PKD3)	10	PRKX	-1
RPS6KA4 (MSK2)	9	MARK3	-1
CAMK1D (CaMKI delta)	9	GRK4	-1
ROCK2	9	CAMK4 (CaMKIV)	-1
RPS6KA5 (MSK1)	8	CHEK1 (CHK1)	-3
PDK1 Direct	8	PRKCG (PKC gamma)	-3
FRAP1 (mTOR)	8	ALK	-3
PRKCH (PKC eta)	7	LCK	-3
FLT3	7	PRKCZ (PKC zeta)	-3
CDK2/cyclin A	7	GRK7	-4
DNA-PK	7	PRKG2 (PKG2)	-4
PRKCB1 (PKC beta I)	7	PDGFRA (PDGFR alpha)	-5
PRKCQ (PKC theta)	7	PRKCB2 (PKC beta II)	-5
AURKA (Aurora A)	7	SGKL (SGK3)	-5
FGFR1	7	TXK	-5
KIT	6	EPHA2	-6
STK4 (MST1)	6	NEK1	-7
PRKG1	6	EGFR (ErbB1)	-8
IGF1R	6	ITK	-9
PLK1	5	GRK5	-10
TBK1	4	CHEK2 (CHK2)	-13
MARK2	4	STK3 (MST2)	-13
PASK	4	PTK2 (FAK)	-20
SYK	4	PIM1	-21
EPHB4	4	MKNK1 (MNK1)	-22

Table S3. The covalent-allosteric Akt inhibitor **27** was screened against a panel of 100 different protein kinases at a compound concentration of 1 µM. Measurements were performed in duplicates and at the particular apparent ATP K_M of each individual kinase employing the activity based Z'LYTE® kinase assay of the SelectScreen® Kinase Profiling Services (ThermoFisher Scientific). The data shows average % inhibition of two independent measurements of assessed kinases.

Kinase	% inhibition **27**	Kinase	% inhibition **27**
AKT1 (PKB alpha)	98	PRKCB1 (PKC beta I)	6
MELK	83	PKN1 (PRK1)	6
AKT2 (PKB beta)	80	MARK2	6
AMPK A2/B1/G1	77	PRKCD (PKC delta)	6
AMPK A1/B1/G1	76	CDK2/cyclin A	6
PASK	61	GRK7	5
AKT3 (PKB gamma)	58	PRKD2 (PKD2)	5
ROCK1	54	MAP4K5 (KHS1)	5
SRC	36	TEK (Tie2)	5
MAPKAPK2	33	PRKCI (PKC iota)	5
ROCK2	31	SYK	5
RPS6KA2 (RSK3)	31	DNA-PK	5
RPS6KB1 (p70S6K)	29	LCK	4
MKNK1 (MNK1)	29	MET (cMet)	4
RET	26	SGK2	4
FGFR1	24	EPHB4	4
MAPKAPK3	23	PDK1 Direct	3
RPS6KA3 (RSK2)	23	MARK3	3
RPS6KA1 (RSK1)	22	TBK1	3
PRKCH (PKC eta)	21	CDK1/cyclin B	3
SGK (SGK1)	21	PRKX	3
GRK6	20	ABL1	3
GSK3B (GSK3 beta)	18	BMX	3
JAK3	17	MAPK3 (ERK1)	3
PRKD1 (PKC mu)	17	ALK	2
PRKCN (PKD3)	16	AURKB (Aurora B)	2
RPS6KA4 (MSK2)	16	EGFR (ErbB1)	2
BRSK1 (SAD1)	16	PLK1	1
PAK4	16	NTRK2 (TRKB)	1
PRKCA (PKC alpha)	16	CHEK2 (CHK2)	1
FLT3	14	ERBB2 (HER2)	0
STK4 (MST1)	14	PDGFRA (PDGFR alpha)	0
RPS6KA5 (MSK1)	13	JAK2	0
CDC42 BPB (MRCKB)	12	NEK1	-1
CAMK1D (CaMKI delta)	12	DCAMKL2 (DCK2)	-1
PRKCQ (PKC theta)	11	SGKL (SGK3)	-1
MAPK1 (ERK2)	11	PTK2 (FAK)	-1
PRKG1	11	GRK5	-2
PRKCE (PKC epsilon)	11	PAK1	-2
GRK4	11	FRAP1 (mTOR)	-2
AURKA (Aurora A)	10	STK3 (MST2)	-3
BTK	10	EPHA2	-3
MAPK14 (p38 alpha) Direct	10	PRKACA (PKA)	-4
KIT	9	PRKG2 (PKG2)	-4
CAMK4 (CaMKIV)	8	PRKCG (PKC gamma)	-6
CDC42 BPA (MRCKA)	8	ACVR1B (ALK4)	-8
ITK	8	TXK	-9
CHEK1 (CHK1)	8	PRKCB2 (PKC beta II)	-12
IGF1R	7	PRKCZ (PKC zeta)	-14
MARK1 (MARK)	7	PIM1	-18

Table S4. The covalent-allosteric Akt inhibitor **30b** was screened against a panel of 100 different protein kinases at a compound concentration of 1 µM. Measurements were performed in duplicates and at the particular apparent ATP K_M of each individual kinase employing the activity based Z'LYTE® kinase assay of the SelectScreen® Kinase Profiling Services (ThermoFisher Scientific). The data shows average % inhibition of two independent measurements of assessed kinases.

Kinase	% inhibition **30b**	Kinase	% inhibition **30b**
AKT1 (PKB alpha)	90	PRKD1 (PKC mu)	6
AKT2 (PKB beta)	76	PRKCB1 (PKC beta I)	6
MELK	52	PLK1	6
AMPK A1/B1/G1	46	GRK4	6
BMX	44	MAPK14 (p38 alpha) Direct	6
AKT3 (PKB gamma)	43	TEK (Tie2)	6
AMPK A2/B1/G1	40	TBK1	5
ROCK1	34	MARK1 (MARK)	5
RPS6KA2 (RSK3)	33	GRK7	5
MAPKAPK2	31	PRKG2 (PKG2)	5
RPS6KA3 (RSK2)	30	MARK2	4
PASK	29	PAK1	4
SGK (SGK1)	29	PRKD2 (PKD2)	4
GSK3B (GSK3 beta)	29	EPHA2	4
RPS6KA1 (RSK1)	27	CAMK1D (CaMKI delta)	3
PRKCA (PKC alpha)	27	DCAMKL2 (DCK2)	3
PRKCE (PKC epsilon)	27	ABL1	3
JAK3	26	MARK3	3
MAPKAPK3	23	CDK1/cyclin B	3
AURKA (Aurora A)	22	MAPK3 (ERK1)	3
BTK	22	GRK6	3
ROCK2	21	SGK2	3
RET	19	AURKB (Aurora B)	2
MAP4K5 (KHS1)	18	TXK	2
SRC	17	CDC42 BPA (MRCKA)	2
JAK2	16	EGFR (ErbB1)	1
SYK	15	ALK	1
FLT3	14	EPHB4	1
PRKCN (PKD3)	14	FRAP1 (mTOR)	1
PRKCH (PKC eta)	14	IGF1R	1
PRKCQ (PKC theta)	13	PDK1 Direct	1
RPS6KA5 (MSK1)	13	PKN1 (PRK1)	1
MAPK1 (ERK2)	13	ITK	1
CDK2/cyclin A	11	ACVR1B (ALK4)	0
RPS6KA4 (MSK2)	11	PRKCG (PKC gamma)	0
MKNK1 (MNK1)	10	MET (cMet)	0
CAMK4 (CaMKIV)	10	PRKCB2 (PKC beta II)	-1
CHEK1 (CHK1)	9	GRK5	-1
KIT	9	ERBB2 (HER2)	-2
DNA-PK	8	NEK1	-2
PRKG1	10	SGKL (SGK3)	-4
FGFR1	10	PRKCZ (PKC zeta)	-4
PRKACA (PKA)	10	PTK2 (FAK)	-4
PRKCD (PKC delta)	9	PRKX	-6
PAK4	8	PDGFRA (PDGFR alpha)	-7
LCK	8	PRKCI (PKC iota)	-9
NTRK2 (TRKB)	8	CHEK2 (CHK2)	-12
RPS6KB1 (p70S6K)	8	PIM1	-15
STK4 (MST1)	7	STK3 (MST2)	-19
BRSK1 (SAD1)	7	CDC42 BPB (MRCKB)	-27

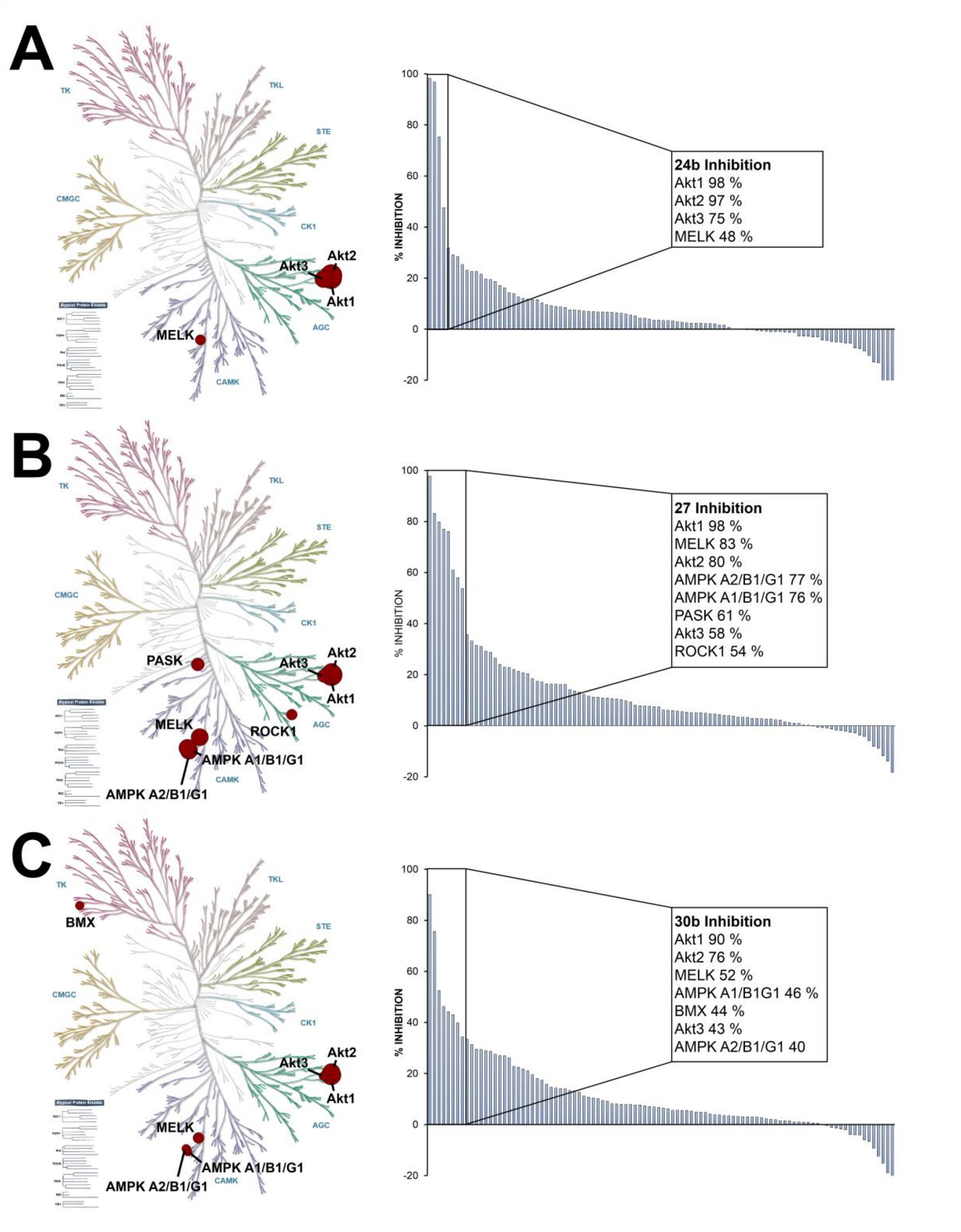

Fig S19 The selectivity profile of the covalent-allosteric Akt inhibitors **24b** (**A**), **27** (**B**) and **30b** (**C**) at 1 µM visualized in a kinome dendrogram adapted from KinMap.[24] The illustration is reproduced with courtesy of Cell Signaling Technology, Inc. (http:/www.cellsignal.com).

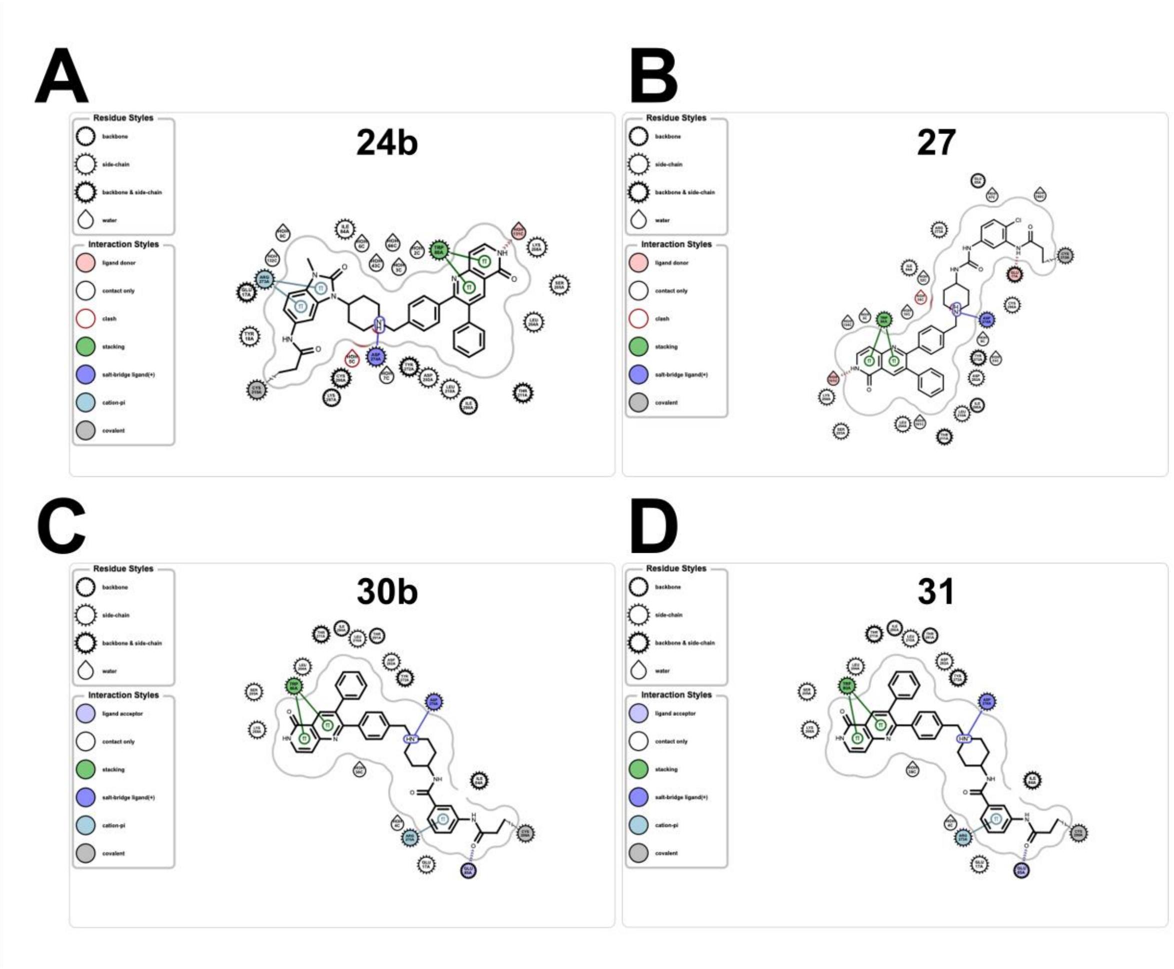

Fig S20 Close-up on the protein-ligand interactions of **24b** (**A**), **27** (**B**), 30b (**C**) and 31 (**D**) based on the complex crystal structures illustrated the differences in the interaction profile of each inhibitor to the protein. The figures were generated by the OEChem/OEDepict toolkits from OpenEye Scientific Software (2018, Santa Fe, NM, www.eyesopen.com).

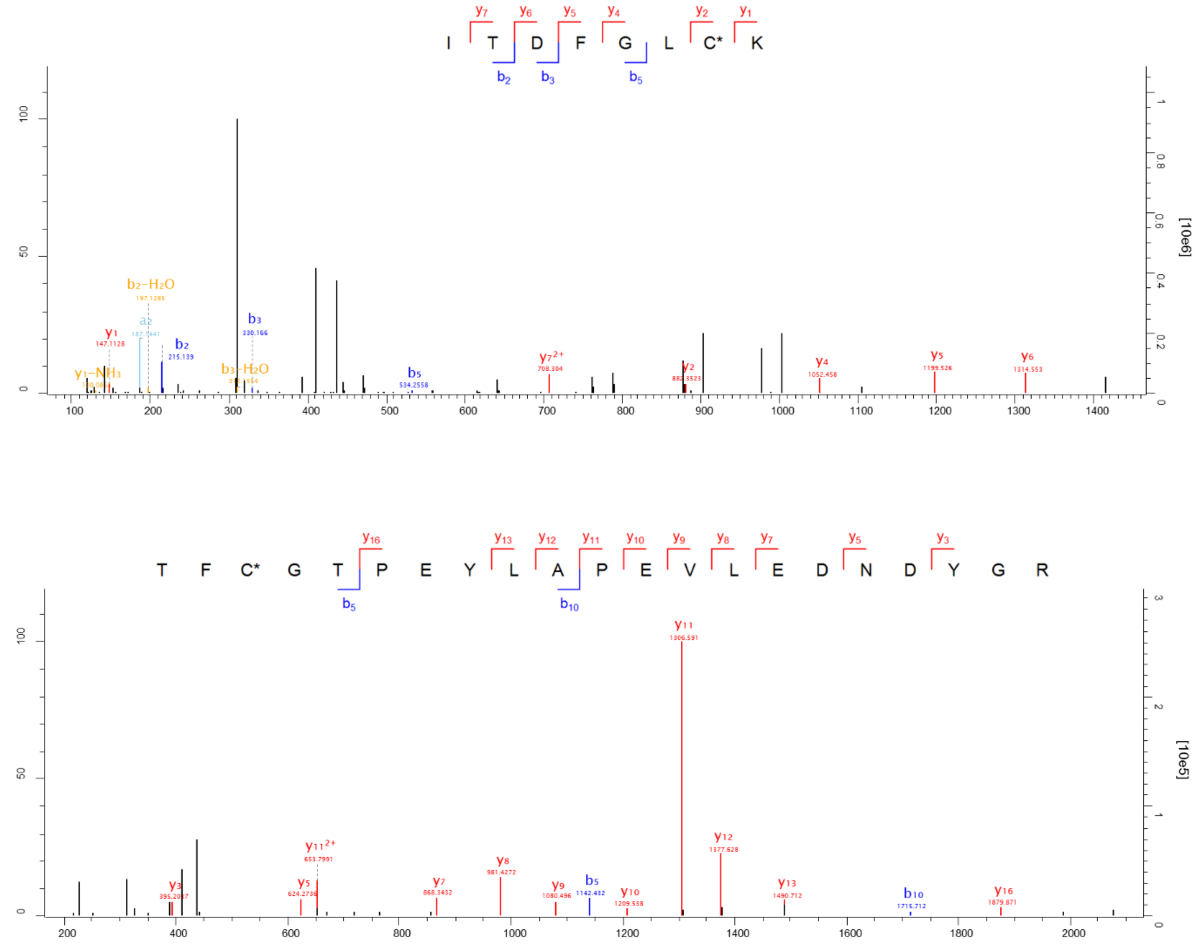

Fig S21 MS-MS analysis of covalent-allosteric Akt inhibitor **27** showed covalent bond formation to Akt1 at Cys296 and Cys310. Purified full-length wtAkt1 was incubated with a 2-fold molar excess of the inhibitor and digested with trypsin after SDS-PAGE. Peptide fragments containing Cys296 and Cys310 labeled with **27** were identified.

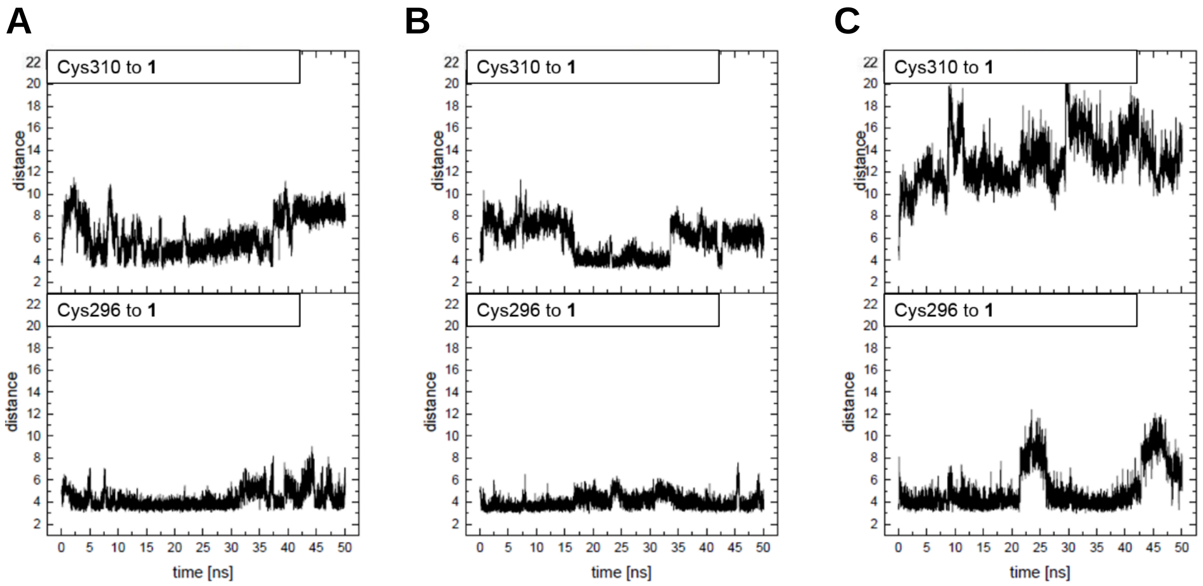

Fig S22 The results from the MD calculations of three completed structures (**A-C**) of Akt1 in complex with borussertib (**1**) were visualized by plotting the distance of the acrylamide Michael acceptor to Cys296 and Cys310 against the simulation time.

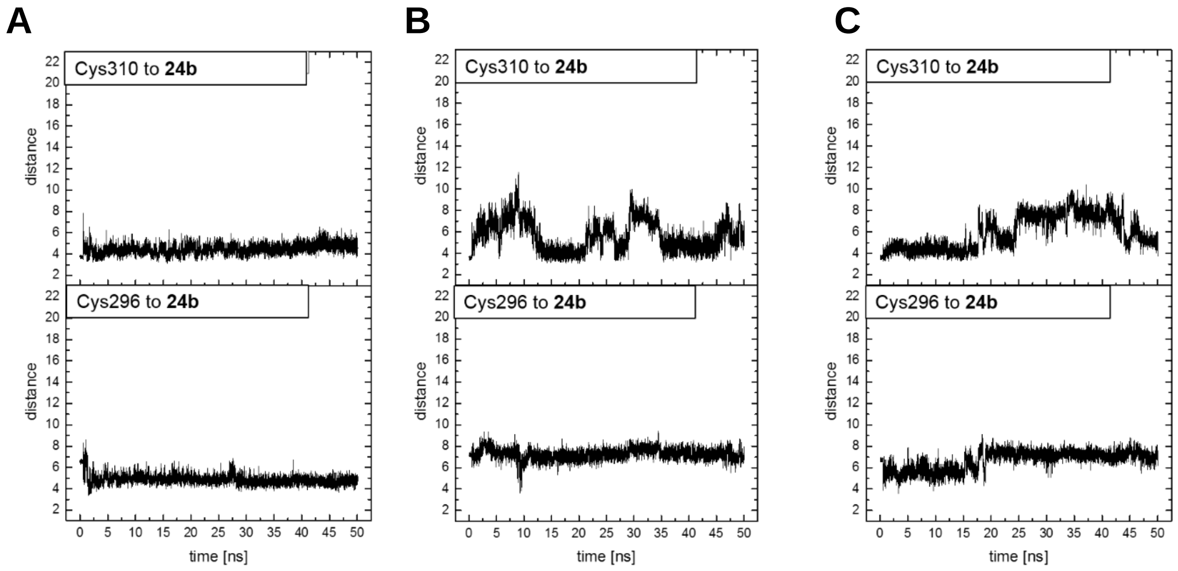

Fig S23 The results from the MD calculations of three completed structures (**A-C**) of Akt1 in complex with **24b** were visualized by plotting the distance of the acrylamide Michael acceptor to Cys296 and Cys310 against the simulation time.

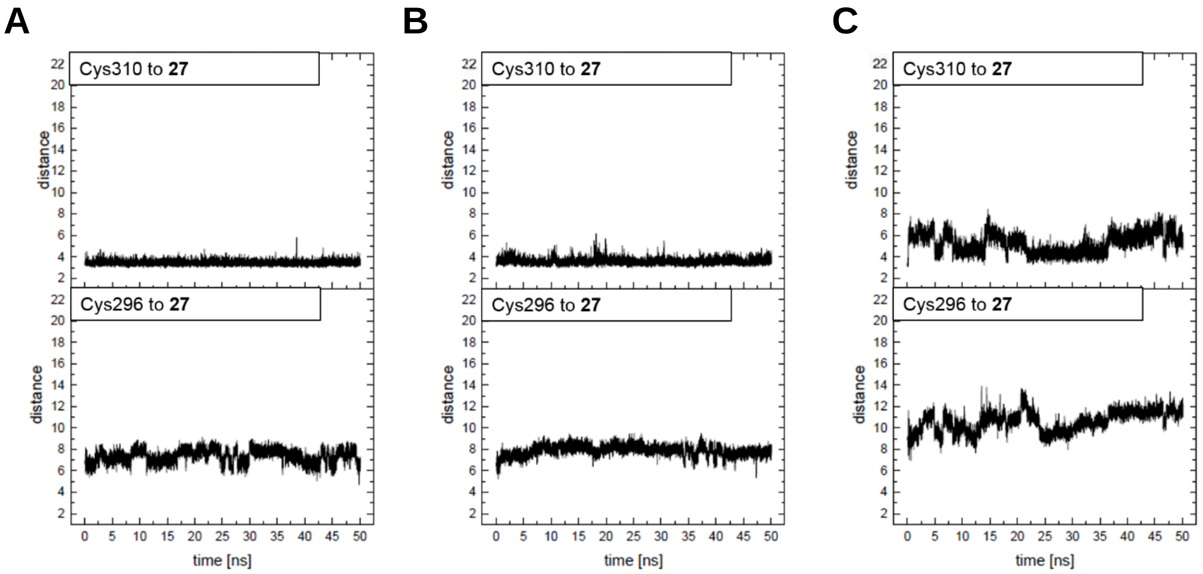

Fig S24 The results from the MD calculations of three completed structures (**A-C**) of Akt1 in complex with **27** were visualized by plotting the distance of the acrylamide Michael acceptor to Cys296 and Cys310 against the simulation time.

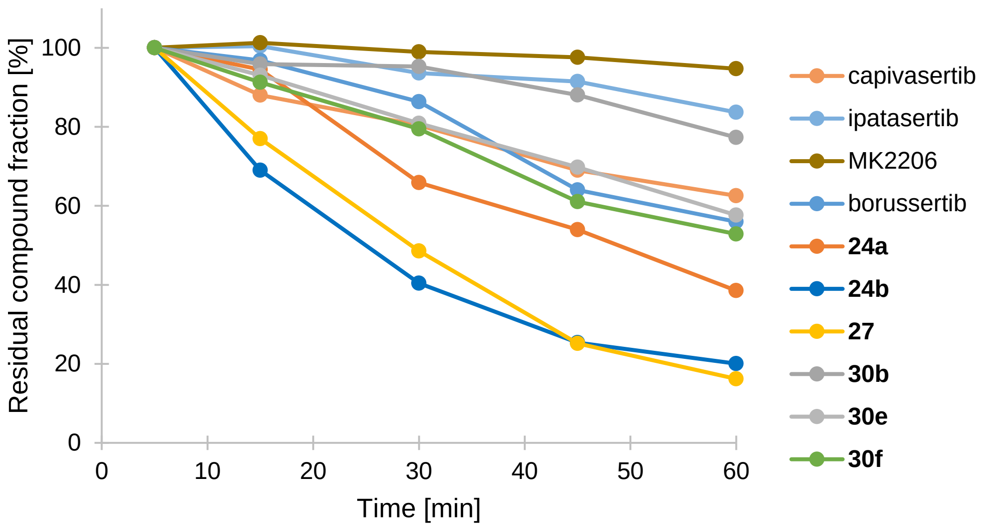

Fig S25 Metabolic stability of a set of inhibitors in murine liver microsomes (MLM). The residual compound fraction is plotted against the time.

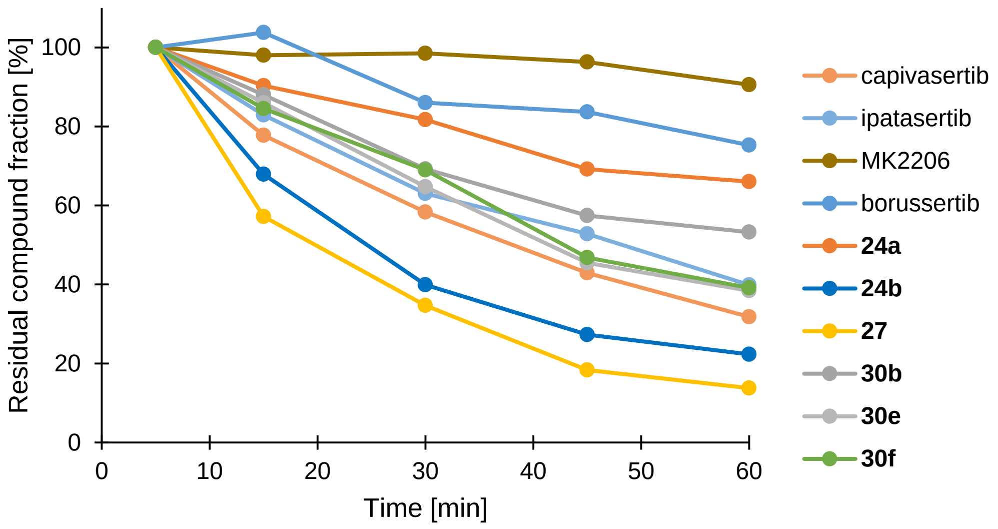

Fig S26 Metabolic stability of a set of inhibitors in human liver microsomes (HLM). The residual compound fraction is plotted against the time.

References

1. Fang, Z.; Simard, J. R.; Plenker, D.; Nguyen, H. D.; Phan, T.; Wolle, P.; Baumeister, S.; Rauh, D., Discovery of inter-domain stabilizers-a novel assay system for allosteric akt inhibitors. *ACS Chem Biol* **2015,** *10* (1), 279-88.

2. Krippendorff, B. F.; Neuhaus, R.; Lienau, P.; Reichel, A.; Huisinga, W., Mechanism-based inhibition: deriving K(I) and k(inact) directly from time-dependent IC(50) values. *J Biomol Screen* **2009,** *14* (8), 913-23.

3. Strohalm, M.; Hassman, M.; Kosata, B.; Kodicek, M., mMass data miner: an open source alternative for mass spectrometric data analysis. *Rapid Commun Mass Spectrom* **2008,** *22* (6), 905-8.

4. Cox, J.; Mann, M., MaxQuant enables high peptide identification rates, individualized p.p.b.-range mass accuracies and proteome-wide protein quantification. *Nat Biotechnol* **2008,** *26* (12), 1367-72.

5. Kabsch, W., Automatic processing of rotation diffraction data from crystals of initially unknown symmetry and cell constants. *Journal of Applied Crystallography* **1993,** *26* (6), 795-800.

6. Read, R., Pushing the boundaries of molecular replacement with maximum likelihood. *Acta Crystallographica Section D* **2001,** *57* (10), 1373-1382.

7. Emsley, P.; Cowtan, K., Coot: model-building tools for molecular graphics. *Acta Crystallographica Section D* **2004,** *60* (12 Part 1), 2126-2132.

8. Schüttelkopf, A. W.; van Aalten, D. M. F., PRODRG: a tool for high-throughput crystallography of protein-ligand complexes. *Acta Crystallographica Section D* **2004,** *60* (8), 1355-1363.

9. Adams, P. D.; Afonine, P. V.; Bunkoczi, G.; Chen, V. B.; Davis, I. W.; Echols, N.; Headd, J. J.; Hung, L.-W.; Kapral, G. J.; Grosse-Kunstleve, R. W.; McCoy, A. J.; Moriarty, N. W.; Oeffner, R.; Read, R. J.; Richardson, D. C.; Richardson, J. S.; Terwilliger, T. C.; Zwart, P. H., PHENIX: a comprehensive Python-based system for macromolecular structure solution. *Acta Crystallographica Section D* **2010,** *66* (2), 213-221.

10. Chen, V. B.; Arendall, W. B., 3rd; Headd, J. J.; Keedy, D. A.; Immormino, R. M.; Kapral, G. J.; Murray, L. W.; Richardson, J. S.; Richardson, D. C., MolProbity: all-atom structure validation for macromolecular crystallography. *Acta Crystallogr D Biol Crystallogr* **2010,** *66* (Pt 1), 12-21.

11. Joosten, R. P.; Long, F.; Murshudov, G. N.; Perrakis, A., The PDB_REDO server for macromolecular structure model optimization. *IUCrJ* **2014,** *1* (Pt 4), 213-20.

12. Schrödinger, L. *The PyMOL Molecular Graphics System, Version 2.0*, 2018.

13. Case DA, Babin V, Berryman JT, Betz RM, Cai Q, Cerutti DS, Cheatham TE, III, Darden TA, Duke RE,Gohlke H, Goetz AW, Gusarov S, Homeyer N, Janowski P, Kaus J, Kolossváry I, Kovalenko A, Lee TS, LeGrand S, Luchko T, Luo R, Madej B, Merz KM, Paesani F, Roe DR, Roitberg A, Sagui C, Salomon-

Ferrer R, Seabra G, Simmerling CL, Smith W, Swails J, Walker RC, Wang J, Wolf RM, Wu X and Kollman PA, AMBER 14, University of California, San Francisco. **2014**.

14. Maier, J. A.; Martinez, C.; Kasavajhala, K.; Wickstrom, L.; Hauser, K. E.; Simmerling, C., ff14SB: Improving the Accuracy of Protein Side Chain and Backbone Parameters from ff99SB. *J Chem Theory Comput* **2015,** *11* (8), 3696-713.

15. Wang, J.; Wolf, R. M.; Caldwell, J. W.; Kollman, P. A.; Case, D. A., Development and testing of a general amber force field. *J Comput Chem* **2004,** *25* (9), 1157-74.

16. Gaussian 03, Revision C.02, Frisch, M. J.; Trucks, G. W.; Schlegel, H. B.; Scuseria, G. E.; Robb, M. A.; Cheeseman, J. R.; Montgomery, Jr., J. A.; Vreven, T.; Kudin, K. N.; Burant, J. C.; Millam, J. M.; Iyengar, S. S.; Tomasi, J.; Barone, V.; Mennucci, B.; Cossi, M.; Scalmani, G.; Rega, N.; Petersson, G. A.; Nakatsuji, H.; Hada, M.; Ehara, M.; Toyota, K.; Fukuda, R.; Hasegawa, J.; Ishida, M.; Nakajima, T.; Honda, Y.; Kitao, O.; Nakai, H.; Klene, M.; Li, X.; Knox, J. E.; Hratchian, H. P.; Cross, J. B.; Bakken, V.; Adamo, C.; Jaramillo, J.; Gomperts, R.; Stratmann, R. E.; Yazyev, O.; Austin, A. J.; Cammi, R.; Pomelli, C.; Ochterski, J. W.; Ayala, P. Y.; Morokuma, K.; Voth, G. A.; Salvador, P.; Dannenberg, J. J.; Zakrzewski, V. G.; Dapprich, S.; Daniels, A. D.; Strain, M. C.; Farkas, O.; Malick, D. K.; Rabuck, A. D.; Raghavachari, K.; Foresman, J. B.; Ortiz, J. V.; Cui, Q.; Baboul, A. G.; Clifford, S.; Cioslowski, J.; Stefanov, B. B.; Liu, G.; Liashenko, A.; Piskorz, P.; Komaromi, I.; Martin, R. L.; Fox, D. J.; Keith, T.; Al-Laham, M. A.; Peng, C. Y.; Nanayakkara, A.; Challacombe, M.; Gill, P. M. W.; Johnson, B.; Chen, W.; Wong, M. W.; Gonzalez, C.; and Pople, J. A.; Gaussian, Inc., Wallingford CT. **2004**.

17. Ditchfield, R.; Hehre, W. J.; Pople, J. A., Self-Consistent Molecular Orbital Methods. 9. Extended Gaussian-type basis for molecular-orbital studies of organic molecules. *J Chem Phys* **1971,** *54* (724), 724.

18. Roothaan, C. C. J., New Developments in Molecular Orbital Theory. *Reviews of Modern Physics* **1951,** *23* (2), 69-89.

19. Jorgensen, W. L.; Chandrasekhar, J.; Madura, J. D., Comparison of simple potential functions for simulating liquid water. *J Chem Phys* **1983,** *79* (2), 926-935.

20. Ryckaert, J.-P.; Ciccotti, G.; Berendsen, H. J. C., Numerical integration of the cartesian equations of motion of a system with constraints: molecular dynamics of n-alkanes. *Journal of Computational Physics* **1977,** *23* (3), 327-341.

21. Miyamoto, S.; Kollman, P. A., SETTLE: an analytical version of the SHAKE and RATTLE algorithm for rigid water models. *J. Comput. Chem.* **1992,** *13* (8), 952-962.

22. Weisner, J.; Landel, I.; Reintjes, C.; Uhlenbrock, N.; Trajkovic-Arsic, M.; Dienstbier, N.; Ladigan, S.; Lindemann, M.; Smith, S.; Quambusch, L.; Scheinpflug, R.; Depta, L.; Gontla, R.; Unger, A.; Müller, H.; Baumann, M.; Schultz-Fademrecht, C.; Guenther, G.; Maghnouj, A.; Müller, M. P.; Pohl, M.; Teschendorf, C.; Wolters, H.; Viebahn, R.; Tannapfel, A.; Uhl, W.; Hengstler, J.; Hahn, S.; Siveke, J.;

Rauh, D., Preclinical Efficacy of Covalent-Allosteric AKT Inhibitor Borussertib in Combination with Trametinib in KRAS-mutant Pancreatic and Colorectal Cancer *submitted* **2018**.

23. Wolle, P.; Müller, M. P.; Rauh, D., Augmented Reality in Scientific Publications-Taking the Visualization of 3D Structures to the Next Level. *ACS Chem Biol* **2018,** *13* (3), 496-499.

24. Eid, S.; Turk, S.; Volkamer, A.; Rippmann, F.; Fulle, S., KinMap: a web-based tool for interactive navigation through human kinome data. *BMC Bioinformatics* **2017,** *18* (1), 16.
